# Supplementary material for: Liver function tests and fibrosis scores in a rural population in Africa: a cross-sectional study to estimate the burden of disease and associated risk factors
Source: BMJ Open. 2020 Mar 31;10(3):e032890. doi: 10.1136/bmjopen-2019-032890 (PMC7170602; doi:10.1136/bmjopen-2019-032890)

SUPPLEMENTARY MATERIAL

Liver function tests and fibrosis scores in a rural population in Africa: a cross-sectional study to estimate the burden of disease and associated risk factors

Geraldine A O'Hara, Jolynne Mokaya, Jeffrey P Hau, Louise O Downs,
Anna L McNaughton[2], Alex Karabarinde, Gershim Asiki,
Janet Seeley, Philippa C Matthews, Robert Newton

CONTENTS

Suppl Table 1: Origin, reference ranges and clinical significance of liver function tests (LFTs)

Suppl Table 2: Scores to estimate liver fibrosis, calculated from liver function tests

Suppl Table 3: Description of characteristics of study participants with liver function test (LFT) results from the Ugandan General Population Cohort (N=8,099)

Suppl Table 4: Median and inter-quartile range for each liver function test with the population divided by risk factors

Suppl Fig 1: Distribution of liver function tests in Uganda General Population Cohort.

Suppl Fig 2: Odds ratio for deranged ALT, AST, APRI, GPR and AST/ALT among participants grouped by sex and age.

Suppl Fig 3: Proportion of Uganda General Population cohort reporting alcohol consumption among individuals with and without AST/ALT ratio >2

Suppl Fig 4: Proportion of Uganda General Population Cohort with elevated GGT, according to AST/ALT ratio. (A) males, with upper limit of normal GGT=61 (B) females, with upper limit of normal GGT=36. P-values by Fisher's Exact Test

Suppl Fig 5: Proportion of Uganda General Population Cohort with blood borne virus (BBV) infection, according to GPR score. P-value by Fisher's Exact Test, showing significant enrichment of BBV infection among individuals with elevated GPR score >0.32.

O'Hara G, *et al. BMJ Open* 2020; 10:e032890. doi: 10.1136/bmjopen-2019-032890

Suppl data Table 1: Origin, reference ranges and clinical significance of liver function tests (LFTs) identified from published literature (7,10,54). LRR: local reference range (derived from populations in Africa); ARR: American reference range.

Biomarker	Origin	LRR	ARR	Common causes of derangement (Abnormal elevation for all markers other than albumin)
Alanine transferase (ALT)	Highest concentration in hepatocytes (small amounts in other tissues: muscles, adipose tissues, intestines, colon, prostate, and brain)	8 – 61*U*/L	Male: 10 - 55 *U*/L<br>Female: 7 - 30 *U*/L	• Acute / chronic viral hepatitis (EBV/CMV/HBV/HCV/HEV)<br>• Alcoholism<br>• Non-alcoholic fatty liver disease (NAFLD)<br>• Drugs: antipsychotics, antibiotics, statins.<br>• Autoimmune hepatitis<br>• Ischaemic liver damage<br>• Haemochromatosis<br>• Wilson's disease<br>• Coeliac disease
Aspartate transferase (AST)	Hepatocytes<br>Cardiac muscle<br>Skeletal muscle	14 - 60 *U*/L	Male: 10 - 40 *U*/L<br>Female: 9 - 32 *U*/L	• The causes listed for raised ALT.<br>• As AST is abundant in skeletal, cardiac and smooth muscle it may also be elevated in patients with cardiac disease, myositis or muscular dystrophy.
Alkaline phosphatase (ALP)	Liver (from biliary epithelium)<br>Bone<br>Placenta	48 - 164 *U*/L	Male: 45 - 115 *U*/L<br>Female: 30–100 *U*/L	• Bile duct obstruction<br>• Primary biliary cirrhosis<br>• Primary sclerosing cholangitis<br>• Drugs: Antibiotics, antiepileptics, MAOI's<br>• Bone growth, and bone disease<br>• Pregnancy<br>• Hepatic congestion from right sided heart failure
Gamma-glutamyl-transferase (GGT)	Liver<br>Kidney<br>Pancreas<br>Intestine<br>Prostate	Nil availabl e	Male: 8 - 61 *U*/L<br>Female: 5 - 36 *U*/L	• Obesity<br>• Hepatobiliary disease<br>• Pancreatic disease<br>• Alcoholism<br>• Drugs: carbamazepine, phenytoin, and barbituates.
Bilirubin (BR)	Red blood cells<br>Liver<br>Bone marrow	2.9 – 37.0 *m*mol/L	0 – 17 *m*mol/L	**Unconjugated hyperbilirubinaemia**<br>• Haemolysis (sickle cell disease and malaria particularly relevant)<br>• Ineffective erythropoiesis<br>• Gilbert's syndrome<br>• Drugs: Rifampicin<br>**Conjugated hyperbilirubinaemia**<br>• Liver disease<br>• Biliary obstruction

O'Hara G, *et al. BMJ Open* 2020; 10:e032890. doi: 10.1136/bmjopen-2019-032890

Albumin (Alb)	Liver; acute phase marker.	35 – 52 g/L	35 – 55 g/L	Lowered in association with:<br>• Chronic liver disease.<br>• Nephrotic syndrome,<br>• Protein losing enteropathy,<br>• Protein Energy Malnutrition<br>• Hypercatabolic states, e.g. in association with malignancy, infection.<br>• Congestive cardiac failure

LRR: Local Reference Ranges derived from a study by Karita et al (19). All ranges are for both male and female.
ARR: American Reference Ranges derived from MGH Clinical Laboratories.
MAOI: Monoamine oxidase inhibitors
[*]No local references were available for Gamma GT
[4]Bilirubin measurement is total Bilirubin concentration measured in *m*mol/L

Suppl Table 2: Scores to estimate liver fibrosis, calculated from liver function tests

**Score**	**Formula**	**Threshold used to predict fibrosis**	**Sensitivity and specificity of fibrosis threshold (derived from previous studies)**
**APRI**	(AST/ULN AST*100) / platelet count	0.7	Sensitivity: 77%<br>Specificity: 72%<br>Derived from meta-analysis of studies of HCV infection (26).
**FIB-4**	(Age in years*AST) / (platelet count * √ALT)	3.25	Specificity: 97%<br>Positive predictive value: 65%<br>Derived from HIV/HCV coinfected individuals (27).
**GPR**	(GGT/ULN of GGT/platelet count) ×100	0.32	Optimal cut-off value for predicting significant fibrosis.<br>Derived from individuals with chronic HBV infection in The Gambia (14).
**RPR**	Red cell distribution width%/ platelet count	0.825	Sensitivity: 63.1%<br>Specificity: 85.5%<br>Positive predictive value: 65%<br>Derived from individuals with chronic HBV infection in China (28).
**S-index**	(1000×GGT)÷(platelet count ×Albumin^2)	0.3	Specificity: 94%<br>Positive predictive value: 87%<br>Accuracy: 68%<br>Derived from individuals with chronic HBV infection in Egypt (29).

AST = Aspartate transaminase at u/l, ULN = upper limit of normal,
ALT = Alanine transaminase at u/l
GGT= Glutamyltansferase at u/l, ULN = upper limit of normal,
Platelet count at 10^9/L

O'Hara G, *et al. BMJ Open* 2020; 10:e032890. doi: 10.1136/bmjopen-2019-032890

Suppl Table 3: Description of characteristics of study participants with liver function test (LFT) results from the Ugandan General Population Cohort (N=8,099)

Variable	Total n(%)	Male n(%)	Female n(%)	p value[1]
	8,099 (100.00)	3,542 (100.00)	4,557 (100.00)	
Age Group				
16-19	2,481 (30.6)	1,268 (35.8)	1,213 (26.6)	<0.001
20-29	1,508 (18.6)	618 (17.5)	890 (19.5)	0.02
30-39	1,349 (16.6)	510 (14.4)	839 (18.4)	<0.001
40-49	1,095 (13.5)	454 (12.8)	641 (14.0)	0.10
50-59	744 (9.2)	315 (8.9)	429 (9.4)	0.42
>60	922 (11.4)	377 (10.8)	545 (12.0)	0.06
Max Education				
None	759 (9.4)	208 (5.9)	551 (12.1)	<0.001
Primary	5,165 (63.8)	2,380 (67.2)	2,785 (61.1)	<0.001
Secondary	1,839 (22.7)	793 (22.3)	1,046 (23.0)	0.54
Higher Level	336 (4.1)	161 (4.5)	175 (3.8)	0.11
SES[2]				
Lower	2,309 (34.6)	1,048 (35.7)	1,261 (33.6)	0.08
Middle	2,175 (32.5)	945 (32.1)	1,230 (32.8)	0.59
Upper	2,203 (32.9)	944 (32.1)	1,259 (33.6)	0.22
HIV Status				
Negative	7,483 (92.5)	3,331 (94.1)	4,152 (91.2)	
Positive	608 (7.5)	208 (5.9)	400 (8.8)	<0.001
Hepatitis B				
Negative	7,878 (97.3)	3,420 (96.6)	4,458 (97.8)	
Positive	220 (2.7)	122 (3.4)	98 (2.2)	<0.001
Hepatitis C				
Negative	8,086 (99.8)	3,533 (99.7)	4,553 (99.9)	
Positive	13 (0.2)	9 (0.3)	4 (0.1)	0.06
BMI[3]				
Normal weight	5,095 (65.1)	2,259 (64.4)	2,836 (65.7)	0.23
Underweight	1,772 (22.7)	1,075 (30.6)	697 (16.1)	<0.001
Overweight/Obese	960 (12.2)	175 (5.0)	785 (18.2)	<0.001
**Alcohol Consumption[4]**				
Never drinkers	5,180 (64.0)	2,120 (59.9)	3,060 (67.2)	
Drinkers	2,919 (36.0)	1,422 (40.1)	1,497 (32.8)	<0.001

[1] p-value calculated to determine whether significant difference between males and females in each category using chi-square test

[2]Socio-economic Score (SES) derived from conducting Principle Component Analysis (PCA) on a statistical software using variables relating to household infrastructure and property ownership

[3]Body Mass Index (BMI) Classification according to WHO (weight/height^2: kg/m^2): Underweight (<18.5 kg/m^2), Normal weight (18.5 – 24.99 kg/m^2), Overweight (25.0 – 29.99 kg/m^2), Obese (>30.0 kg/m^2)

[4] Alcohol consumption based on self-reported history of consuming alcohol vs never consuming alcohol

O'Hara G, *et al. BMJ Open* 2020; 10:e032890. doi: 10.1136/bmjopen-2019-032890

Suppl Table 4: Median and inter-quartile range for each liver function test, with the population divided by risk factors.

	ALT [1,6] *Median (IQR)*	*AST* [1] *Median (IQR)*	*ALP* [1] *Median (IQR)*	*GGT* [1] *Median (IQR)*	*Total BR* [1] *Median (IQR)*	*FIB-4* [1] *Median (IQR)*	*APRI* [1,#] *Median (IQR)*	*GPR* [1] *Median (IQR)*	*S-Index* [3] *Median (IQR)*
Sex									
Male	19.4 (15.6-25.0)	27.9 (23.9-33.5)	97.1 (74.3-209.9)	21.6 (15.5-32.8)	8.9 (5.9-14.1)	0.90 (0.49-1.57)	0.24 (0.18-0.33)	0.17 (0.12-0.30)	0.06 (0.04-0.11)
Female	16.4 (13.0-21.3)	23.1 (19.8-27.4)	89.5 (68.5-123.2)	16.9 (12.3-24.4)	6.9 (4.8-10.4)	0.81 (0.47-1.40)	0.18 (0.14-0.24)	0.21 (0.15-0.32)	0.04 (0.03-0.07)
p-value	***	***	***	***	***	**	***	***	***
Age									
<19	17.8 (14.5-22.1)	26.5 (22.6-31.4)	218.8 (134.5-306.0)	16.2 (12.6-21.2)	7.26 (4.97-11.4)	0.42 (0.33-0.54)	0.19 (0.15-0.25)	0.15 (0.11-0.21)	0.04 (0.03-0.05)
20-29	18.0 (13.8-23.8)	23.8 (19.9-28.5)	82.4 (66.8-102.8)	17.7 (12.5-25.3)	8.59 (5.69-13.9)	0.65 (0.50-0.90)	0.19 (0.15-0.30)	0.18 (0.13-0.27)	0.05 (0.03-0.07)
30-39	18.3 (13.9-24.5)	24.1 (20.1-29.6)	74.8 (59.9-94.5)	20.0 (13.7-32.0)	7.88 (5.28-12.7)	1.02 (0.78-1.34)	0.21 (0.16-0.29)	0.23 (0.16-0.39)	0.06 (0.04-0.11)
40-49	18.4 (14.3-24.1)	25.1 (21.0-31.1)	74.7 (60.3-90.0)	22.0 (14.8-36.7)	7.78 (5.08-12.1)	1.36 (1.03-1.80)	0.23 (0.16-0.32)	0.25 (0.17-0.45)	0.07 (0.04-0.14)
50-59	18.3 (14.2-23.6)	25.7 (21.5-32.0)	83.2 (71.6-99.4)	24.1 (16.7-38.1)	7.41 (5.41-11.4)	1.66 (1.32-2.29)	0.22 (0.17-0.32)	0.29 (0.19-0.48)	0.08 (0.05-0.13)
>60	15.4 (12.3-20.0)	24.9 (21.4-29.9)	89.5 (73.9-107.9)	23.7 (16.2-35.7)	7.16 (4.90-10.4)	2.20 (1.61-3.23)	0.20 (0.16-0.29)	0.26 (0.18-0.43)	0.08 (0.04-0.12)
p-value	***	***	***	***	***	***	***	***	***
Alcohol									
No	17.6 (13.9-22.4)	24.9 (21.1-29.7)	103.0 (74.3-204.3)	17.3 (12.8-23.7)	7.57 (5.16-12.0)	0.61 (0.40-1.08)	0.20 (0.15-0.27)	0.17 (0.12-0.26)	0.04 (0.03-0.07)
Yes	17.9 (13.9-24.0)	25.2 (21.4-31.8)	83.0 (67.1-103.9)	23.2 (15.6-38.9)	7.77 (5.24-12.1)	1.40 (0.88-2.14)	0.22 (0.17-0.32)	0.26 (0.17-0.46)	0.07 (0.04-0.14)
p-value	**	***	***	***	*ns*	***	***	***	***
BMI [2]									
Normal	17.9 (14.3-22.8)	27.5 (23.3-32.8)	185.2 (90.9-297.0)	18.5 (13.7-26.0)	7.09 (4.85-11.2)	0.53 (0.35-1.38)	0.21 (0.16-0.29)	0.17 (0.12-0.27)	0.04 (0.03-0.08)
Under-weight	17.9 (14.1-23.2)	25.0 (21.3-30.1)	88.1 (69.1-122.5)	18.7 (13.7-27.8)	8.00 (5.39-12.8)	0.90 (0.51-1.52)	0.21 (0.15-0.39)	0.20 (0.14-0.32)	0.05 (0.03-0.08)
Over-weight	18.0 (13.9-23.1)	22.7 (19.4-27.0)	81.3 (65.2-99.1)	21.8 (14.8-31.5)	7.28 (5.12-10.9)	0.96 (0.61-1.42)	0.18 (0.14-0.25)	0.23 (0.16-0.38)	0.06 (0.04-0.09)
p-value	*ns*	***	***	***	***	***	***	***	*ns*
HIV status									
Negative	17.6 (13.9-22.7)	25.0 (21.2-30.0)	93.7 (71.8-151.3)	18.4 (13.4-26.6)	7.89 (5.40-12.4)	0.82 (0.46-1.46)	0.20 (0.15-0.28)	0.19 (0.13-0.29)	0.05 (0.03-0.08)
Positive	19.4 (14.9-26.5)	27.1 (22.3-33.5)	83.2 (65.1-107.8)	30.6 (16.7-58.3)	5.06 (3.53-7.66)	1.21 (0.76-1.70)	0.23 (0.18-0.34)	0.44 (0.20-0.79)	0.11 (0.05-0.20)
p-value	***	***	***	***	***	***	***	***	***
HBV status									
Negative	17.7 (13.9-22.7)	25.1 (21.1-30.2)	92.6 (71.3-144.8)	18.6 (13.5-27.6)	7.62 (5.19-12.0)	0.84 (0.47-1.47)	0.20 (0.15-0.27)	0.19 (0.13-0.31)	0.05 (0.03-0.08)
Positive	22.0 (15.8-29.2)	28.2 (23.0-39.0)	91.9 (71.6-131.5)	23.5 (15.4-37.6)	8.76 (5.34-13.4)	1.01 (0.55-1.80)	0.25 (0.19-0.48)	0.32 (0.17-0.52)	0.11 (0.06-0.20)
p-value	***	***	*ns*	***	*	*ns*	***	***	***

[1] ALT - Alanine Transminase, AST - Aspartate Transminase, GGT - Gamma-glutamyl transpeptidase, ALP - Alkaline Phosphatase, TB - Total Bilirubin, FIB-4 - fibrosis 4, APRI - AST to Platelet Ratio Index, GPR - GGT to platelet ratio, IQR – inter-quartile range

[2] Body Mass Index (BMI) Classification according to WHO (weight/height^2: kg/m^2): Underweight (<18.5 kg/m^2), Normal weight (18.5 – 24.99 kg/m^2), Overweight (25.0 – 29.99 kg/m^2), Obese (>30.0 kg/m^2)

[3] An S-index score of >0.3 is suggestive of liver fibrosis

[#] APRI score calculated using ULN of AST using African reference range

p-value significance level: * = (p<0.05), ** = (p<0.01), *** = (p<0.001), *ns* = (p>0.05)

O'Hara G, *et al. BMJ Open* 2020; 10:e032890. doi: 10.1136/bmjopen-2019-032890

Suppl Fig 1: Distribution of liver function tests in Uganda General Population Cohort. Top row: ALT -alanine transferase, AST – aspartate transferase, GGT – gamma glutamyl transferase. Bottom row: BR – bilirubin, ALP – alkaline phosphatase.

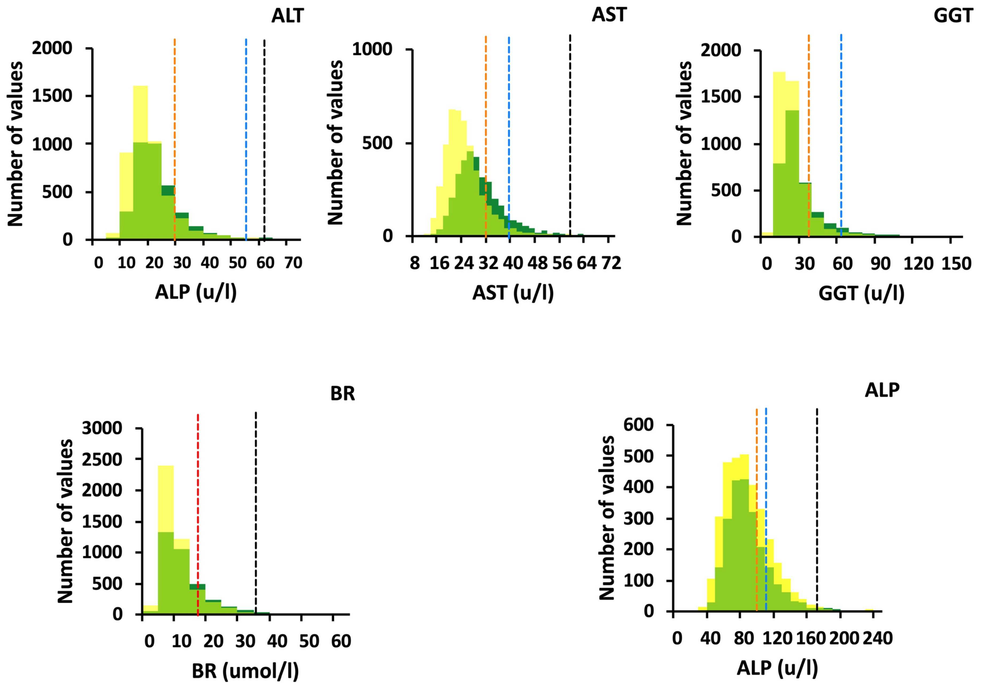

O'Hara G, *et al. BMJ Open* 2020; 10:e032890. doi: 10.1136/bmjopen-2019-032890

Suppl Fig 2: Odds ratio for deranged ALT, AST, APRI, GPR and AST/ALT among participants of te General Population Cohort in Uganda. Data grouped by sex and age, by multivariate analysis.

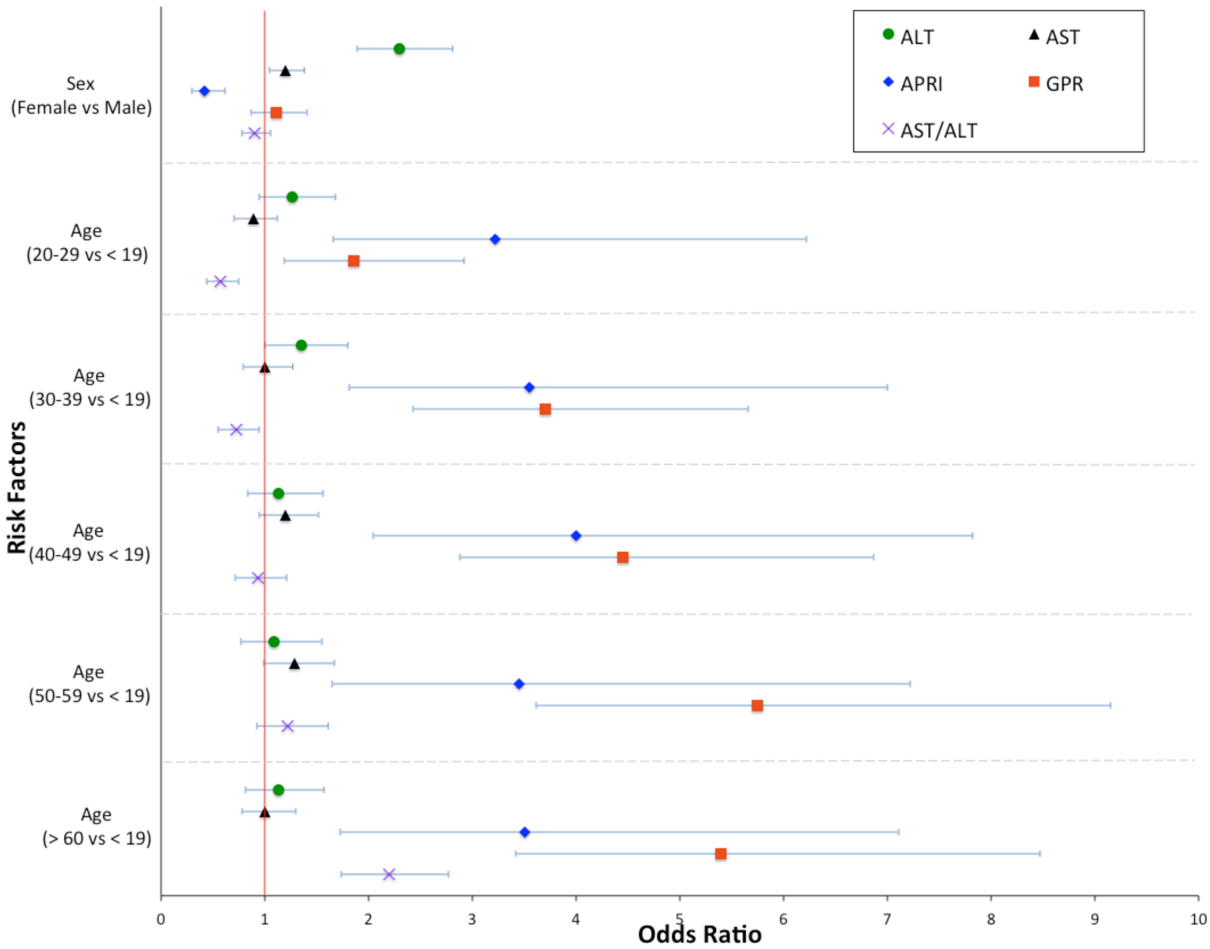

Suppl Fig 3: Proportion of Uganda General Population Cohort reporting alcohol consumption among individuals with and without AST/ALT ratio >2

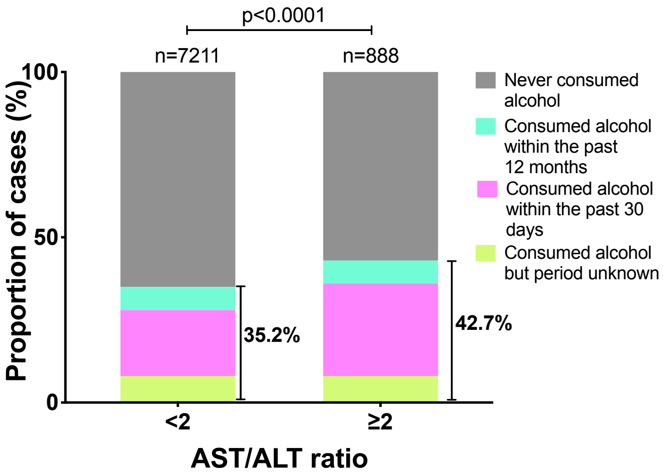

O'Hara G, *et al. BMJ Open* 2020; 10:e032890. doi: 10.1136/bmjopen-2019-032890

Suppl Fig 4: Proportion of Uganda General Population Cohort with elevated GGT, according to AST/ALT ratio. (A) males, with upper limit of normal GGT=61 (B) females, with upper limit of normal GGT=36. P-values by Fisher's Exact Test

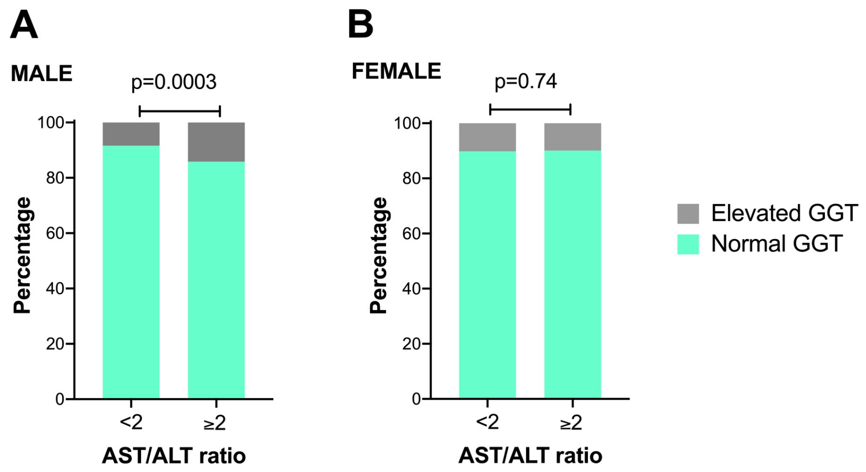

O'Hara G, *et al. BMJ Open* 2020; 10:e032890. doi: 10.1136/bmjopen-2019-032890

Suppl Fig 5: Proportion of Uganda General Population Cohort with blood borne virus (BBV) infection, according to GPR score. P-value by Fisher's Exact Test, showing significant enrichment of BBV infection among individuals with elevated GPR score >0.32.

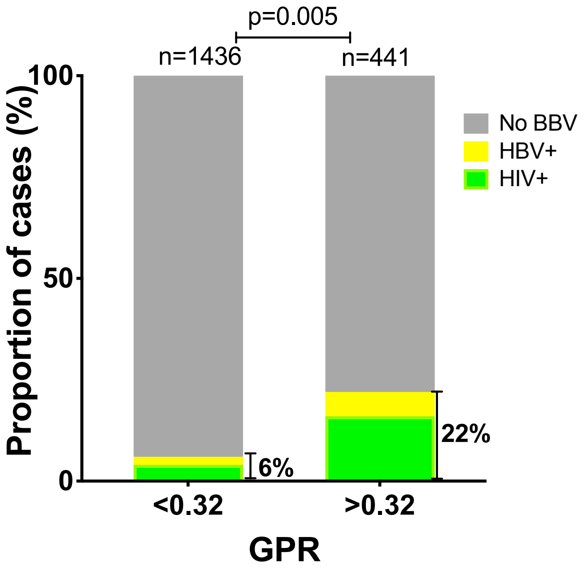

Supplement: Supplementary data [file bmjopen-2019-032890supp001.pdf]
