# Supplementary material for: Liver function tests and fibrosis scores in a rural population in Africa: a cross-sectional study to estimate the burden of disease and associated risk factors
Source: BMJ Open. 2020 Mar 31;10(3):e032890. doi: 10.1136/bmjopen-2019-032890 (PMC7170602; doi:10.1136/bmjopen-2019-032890)
Supplement: Author's manuscript [file bmjopen-2019-032890.draft_revisions.pdf]

BMJ Open

BMJ Open is committed to open peer review. As part of this commitment we make the peer review history of every article we publish publicly available.

When an article is published we post the peer reviewers' comments and the authors' responses online. We also post the versions of the paper that were used during peer review. These are the versions that the peer review comments apply to.

The versions of the paper that follow are the versions that were submitted during the peer review process. They are not the versions of record or the final published versions. They should not be cited or distributed as the published version of this manuscript.

BMJ Open is an open access journal and the full, final, typeset and author-corrected version of record of the manuscript is available on our site with no access controls, subscription charges or pay-per-view fees (http://bmjopen.bmj.com).

If you have any questions on BMJ Open's open peer review process please email info.bmjopen@bmj.com

BMJ Open

Liver function tests and fibrosis scores in a rural population in Africa: a cross-sectional study to estimate the burden of disease and associated risk factors

Journal:	*BMJ Open*
Manuscript ID	bmjopen-2019-032890
Article Type:	Research
Date Submitted by the Author:	20-Jul-2019
Complete List of Authors:	O'Hara, Geraldine; London School of Hygiene and Tropical Medicine, Faculty of Infectious and Tropical Diseases<br>Mokaya, Jolynne; Nuffied Department of Medicine<br>Hau, Jeffrey; London School of Hygiene and Tropical Medicine, Faculty of Infectious and Tropical Diseases<br>Downs, Louise; Oxford University Hospitals NHS Foundation Trust, Department of Microbiology and Infectious Diseases<br>McNaughton, Anna; University of Oxford, Nuffield Department of Medicine<br>Karabarinde, Alex; Medical Research Council/Uganda Virus Research Institute<br>Asiki, G; Medical Research Council/Uganda Virus Research Institute<br>Seeley, Janet; London School of Hygiene and Tropical Medicine, Department of Global Health &Development<br>Matthews, Philippa; University of Oxford, Nuffield Department of Medicine; Oxford University Hospitals NHS Foundation Trust, Department of Microbiology and Infectious Diseases<br>Newton, Robert; Medical Research Council/Uganda Virus Research Institute
Keywords:	Epidemiology < TROPICAL MEDICINE, Hepatology < INTERNAL MEDICINE, HIV & AIDS < INFECTIOUS DISEASES

SCHOLARONE™
Manuscripts

BMJ

I, the Submitting Author has the right to grant and does grant on behalf of all authors of the Work (as defined in the below author licence), an exclusive licence and/or a non-exclusive licence for contributions from authors who are: i) UK Crown employees; ii) where BMJ has agreed a CC-BY licence shall apply, and/or iii) in accordance with the terms applicable for US Federal Government officers or employees acting as part of their official duties; on a worldwide, perpetual, irrevocable, royalty-free basis to BMJ Publishing Group Ltd ("BMJ") its licensees and where the relevant Journal is co-owned by BMJ to the co-owners of the Journal, to publish the Work in this journal and any other BMJ products and to exploit all rights, as set out in our licence.

The Submitting Author accepts and understands that any supply made under these terms is made by BMJ to the Submitting Author unless you are acting as an employee on behalf of your employer or a postgraduate student of an affiliated institution which is paying any applicable article publishing charge ("APC") for Open Access articles. Where the Submitting Author wishes to make the Work available on an Open Access basis (and intends to pay the relevant APC), the terms of reuse of such Open Access shall be governed by a Creative Commons licence – details of these licences and which Creative Commons licence will apply to this Work are set out in our licence referred to above.

Other than as permitted in any relevant BMJ Author's Self Archiving Policies, I confirm this Work has not been accepted for publication elsewhere, is not being considered for publication elsewhere and does not duplicate material already published. I confirm all authors consent to publication of this Work and authorise the granting of this licence.

For peer review only

Liver function tests and fibrosis scores in a rural population in Africa: a cross-sectional study to estimate the burden of disease and associated risk factors

Geraldine A O'Hara[1,3*], Jolynne Mokaya[2*], Jeffrey P Hau [1,3], Louise O Downs [2.4], Anna L McNaughton [2], Alex Karabarinde[3], Gershim Asiki[3,5], Janet Seeley[3,6], Philippa C Matthews[2,4#] Robert Newton[3,7#]

[1]Faculty of Infectious and Tropical Diseases, London School of Hygiene and Tropical Medicine, London, United Kingdom

[2]Nuffield Department of Medicine, University of Oxford, Medawar Building for Pathogen Research, South Parks Road, Oxford OX1 3SY, UK;

[3] Medical Research Council/Uganda Virus Research Institute and London School of Hygiene and Tropical Medicine Uganda Research Unit, Entebbe, Uganda;

[4]Department of Infectious Diseases and Microbiology, Oxford University Hospitals NHS Foundation Trust, John Radcliffe Hospital, Headington, Oxford OX3 9DU, UK;

[5]African Population and Health Research Center, Nairobi, Kenya;

[6]Department of Global Health and Development, London School of Hygiene and Tropical Medicine, Tavistock Place, London, UK;

[7]Department of Health Sciences, University of York, York, United Kingdom;

* These authors contributed equally

\# These authors contributed equally

CORRESPONDING AUTHOR: Philippa Matthews, Email: philippa.matthews@ndm.ox.ac.uk

KEY WORDS

Hepatitis, liver function tests, epidemiology, prevalence, alcohol, alcoholic liver disease, HBV, HIV, Uganda, Africa, cirrhosis, fibrosis

WORD COUNT: 3999

ACKNOWLEDGEMENTS: Nil

For peer review only

ABBREVIATIONS

- ALT – alanine transferase
- APRI - AST to Platelet Ratio Index
- ARR – American reference range
- AST – aspartate transaminase
- BR – bilirubin
- BBV – blood borne virus (HIV, HBV, HCV)
- FIB-4- fibrosis-4
- GGT – gamma glutamyl-transferase
- GPC – general population cohort, Uganda
- GPR - GGT to platelet ratio
- HBV – hepatitis B virus
- HCV – hepatitis C virus
- HIV – human immunodeficiency virus
- LFTs – liver function tests
- LRR – local reference range
- NAFLD – non-alcoholic fatty liver disease
- PAR – population attributable risk
- RPR- red cell distribution width to platelet ratio
- sSA – sub Saharan Africa
- ULN – upper limit of normal
- USS – ultrasound scan
- WHO – World Health Organisation

For peer review only

ABSTRACT

Introduction: Liver disease is a major cause of morbidity and mortality in sub-Saharan Africa. However, its prevalence, distribution and aetiology have not been well characterised. We examined liver function tests (LFTs) and calculated liver fibrosis scores in a rural population in Uganda.

Methodology: A cross-sectional survey of LFTs was undertaken in 2011 in a rural population cohort in South-Western Uganda. We classified abnormal LFTs based on reference ranges set in America and in Africa. We derived fibrosis scores (AST to Platelet Ratio Index, fibrosis-4, GGT to platelet ratio, red cell distribution width to platelet ratio, and S-index) to evaluate the potential prevalence of liver disease. We collected information about alcohol intake, and infection with HIV, HBV and HCV, to determine the contribution made by these factors to liver inflammation or fibrosis.

Results: Data were available for 8,099 participants (median age 30 years; 56% female). The prevalence of HBV, HCV and HIV infection were 3%, 0.2% and 8%, respectively. The prevalence of abnormal LFTs was higher based on the American reference range compared to the African reference range (e.g. for AST 13% vs 3%, respectively). The prevalence of AST/ALT ratio >2 was 11%, suggestive of alcoholic hepatitis. The highest prevalence of fibrosis was suggested by the GPR score, with 24% of the population falling above the threshold for fibrosis. By multivariate analysis, elevated LFTs and fibrosis scores were most consistently associated with older age, male sex, being under-weight, infection with HIV or HBV, and alcohol consumption. Based on population attributable risk, the highest proportion of elevated fibrosis scores was associated with alcohol use (e.g. 64% of elevated S-index scores).

Conclusion: Further work is required to determine normal reference ranges for LFTs in this setting, to evaluate the specificity and sensitivity of fibrosis scores, and to determine aetiology of liver disease.

For peer review only

For peer review only - http://bmjopen.bmj.com/site/about/guidelines.xhtml

ARTICLE SUMMARY

Strengths and Limitations of the Study

- Liver disease is not well characterised in many parts of sSA despite the high prevalence of chronic viral infections (HIV, HBV and HCV), and potential exposure to hepatotoxins including alcohol, aflatoxins and traditional herbal medicine; this study is therefore an important addition to the existing literature.
- This is a cross sectional study of a large well-defined population cohort in rural South-Western Uganda where the burden of liver disease and its aetiology is not well described, based on liver function tests (LFTs).
- The approach has allowed us to develop insights into some of the risk factors for liver disease, and estimate the burden of liver disease that can currently be accounted for.
- LFTs are a blunt tool for assessment of liver health, with many potential confounding factors. This current study only accounts for a limited range of aetiological agents.
- LFTs were measured at only one point in time, potentially overcalling liver disease as a result of transient abnormalities.

INTRODUCTION

Liver disease causes an estimated 200,000 deaths each year in sub-Saharan Africa (sSA) as a result of liver cirrhosis and hepatocellular carcinoma (1). More than 80% of Africa's burden of liver disease has been attributed to endemic blood borne virus (BBV) infections, such as HIV, hepatitis B (HBV) and hepatitis C (HCV), alcohol, hepatotoxic medications (including traditional and herbal medicines), non-alcoholic fatty liver disease (NAFLD) and exposure to aflatoxins (1–3). However, the prevalence, distribution and aetiology of liver disease in many parts of Africa have not been well characterised, and the neglect of cirrhosis has recently been highlighted (2). In order to improve screening for liver disease, and to implement appropriate investigations and intervention, we have undertaken a survey of liver function tests (LFTs) together with demographic data for a large rural cohort in South-Western Uganda (4).

LFTs are usually the first approach to evaluation of liver disease (reference ranges and causes of derangement are summarised in Suppl Table 1). In addition, liver synthetic function can be assessed by measuring prothrombin time; platelet production may be decreased in chronic liver disease due to hypersplenism, decreased thrombopoietin levels and bone marrow suppression (5). Abnormal LFTs are often non-specific and can arise transiently in association with many acute illnesses or usage of

For peer review only - http://bmjopen.bmj.com/site/about/guidelines.xhtml

medications. However, persistent derangement can indicate chronic liver disease, with associated morbidity and mortality (6). The pattern of derangement can sometimes help to establish aetiology – for example AST/ALT ratio >2 is characteristically associated with alcoholic hepatitis (7,8).

Determination of the origin of liver disease and ascertainment of treatment requirements necessitates accurate characterisation of the degree of liver disease. Liver biopsy allows objective grading of fibrosis and can provide information about the likely aetiology of liver disease based on specific changes to cellular architecture. However, biopsy is costly, requires experts to undertake the procedure and analyse samples, and exposes patients to potentially life-threatening risks. Imaging can also be employed to assess fibrosis. Typically, this comprises ultrasound-based techniques, including fibroscan to derive elastography scores. In most low and middle-income settings, evaluation of liver disease currently depends on use of non-invasive (blood) markers, combined with ultrasound and/or fibroscan when available.

Non-invasive fibrosis blood tests are relatively simple and offer a safe route to assess for liver fibrosis, appealing in resource limited settings. Scores of liver fibrosis, such as AST to Platelet Ratio Index (APRI), fibrosis-4 (FIB-4), GGT to platelet ratio (GPR), red cell distribution width to platelet ratio (RPR) and S-index have been derived using liver enzymes (ALT, AST, GGT) in combination with platelet count. However diagnostic accuracy is not well established in sSA and can be influenced by the population being assessed and the nature of underlying liver disease (9–14). GPR has recently been reported as an independent predictor of significant fibrosis in treatment naïve Gambian patients with chronic hepatitis B (CHB) infection (12). However, further studies are needed to determine the specificity and sensitivity of different scores in different settings.

Appropriate reference ranges for LFTs are crucial for optimising the detection of underlying liver disease (15). Application of reference ranges defined in one setting to different populations may lead to either under- or over-estimation of abnormalities (15–17). As well as being dependent on the population being assessed, the distribution of LFTs in any given setting can also be influenced by the type of instrument, reagents used, and the strength of quality assurance (17). Efforts have been made to establish ‘population-specific’ reference ranges (16,18); one example is through the application of cross-sectional data from seven South-Eastern African countries (16). However, such local reference ranges for Africa have been derived from cross-sectional data collected in adults without addressing the potential prevalence of underlying liver disease. Thus, while American reference ranges potentially over-estimate of the burden of liver disease in an African setting, it is also possible that

For peer review only - http://bmjopen.bmj.com/site/about/guidelines.xhtml

locally derived reference ranges under-estimate the burden (as they are based on thresholds that have been derived from populations in which liver disease is highly prevalent).

We here set out to assess LFTs and fibrosis scores derived from a large, well defined population cohort in rural South-Western Uganda (19). We applied reference ranges set in both America and in Africa (16,20), in order to assess the possible burden of liver disease, highlighting the discrepancies that arise as a result of the difference between thresholds. We derived fibrosis scores to further evaluate the potential prevalence of liver disease in this setting and to estimate the contributions of alcohol and BBVs to the burden of disease.

METHODS

Study design and study population

We conducted a cross-sectional study in a rural population in Kyamulibwa, in the Kalungu district of South-Western Uganda as part of the survey of the General Population Cohort (GPC). The GPC is a community-based cohort established in 1989 with funding from the UK Medical Research Council (MRC) in collaboration with the Uganda Virus Research Institute (UVRI) (21). Regular census and medical surveys have been conducted in this population cohort. In 2011, data collection included screening for viral hepatitis and LFTs among 8,145 adults (≥16 years), which we used for this analysis.

Data collection

Demographic and health history data were collected using questionnaires and standardised procedures described elsewhere (21,22). Blood samples were drawn at home and transported for testing at the Medical Research Council central laboratories in Entebbe. LFTs (serum AST, ALT, ALP, GGT and BR) were measured using a Cobas Integra 400 plus machine, with Roche reagents. Screening for HIV testing was done using an algorithm recommended by the Uganda Ministry of Health, based on initial screening with a rapid test. If the test result was negative, the participant was considered to be HIV negative. If the test result was positive, the sample was re-tested with the rapid test HIV-1 or -2 Stat-Pak. If both tests resulted in a positive result, the participant was diagnosed as HIV positive. If the tests gave discordant results, the sample was further evaluated with the rapid test Uni-Gold Recombinant HIV-1/2. For those samples assessed by all three tests, two positive test results were interpreted as positive, and two negative results were considered negative. HBV surface antigen (HBsAg) testing was conducted using Cobas HBsAg II (2011-08 V10), and those who tested positive were invited for further serologic testing. HCV was tested using a combination of

For peer review only - http://bmjopen.bmj.com/site/about/guidelines.xhtml

immunoassays followed by PCR, as previously described (23). Normal serum levels of liver enzymes were classified according to the American reference range (ARR, MGH Clinical Laboratories) and Local Reference Ranges (LRR, (16); Suppl Table 1). We excluded individuals ≤19 years from ALP analysis, since elevated ALP can be attributable to bone growth in teenagers.

Data from the full blood count was used to calculate fibrosis scores (mean corpuscular volume, MCV and platelet count). This was collected starting part-way through the 2011 data collection period; the data are, therefore, population-based, although based on only a subset of the whole cohort (n=1,877).

Calculation of fibrosis scores and AST/ALT ratio

Where data were available (n=1,877), we calculated APRI, FIB-4, GPR, RPR and S-Index. The formulae for calculating these scores are presented in Suppl Table 2, along with the sensitivity and specificity of each, based on previous studies. We used previously established thresholds to suggest the presence of liver fibrosis, as follows: APRI >0.7 (24), FIB-4 >3.25 (25), GPR >0.32 (12), RPR >0.825 (26), S-index >0.3 (27). We calculated AST/ALT ratio; a score >2 has been associated with alcoholic hepatitis (8).

Statistical Analysis

We analysed data using standard statistical software, Stata/IC 13 (Stata Corporation, College Station, USA) and GraphPad Prism v7·0. We summarised participant baseline characteristics using proportions (%) and these were stratified by sex. We reported prevalence and distribution of LFTs, laboratory markers of fibrosis and elastography scores using descriptive statistics. We reported p-values from chi-square tests, comparing the proportions of each potential risk factor between male and female participants.

We used logistic regression in our univariate and multivariate analyses, using the threshold for significance set at 0.05, to estimate the odd ratios (OR), along with its 95% confidence intervals (95% CI), to identify potential factors associated with abnormal LFTs and liver fibrosis scores, using a forward stepwise approach to develop our multivariate models. We added risk factors that were identified in the age and sex adjusted univariate analysis to the multivariate model. The final multivariate models for each LFT and liver fibrosis score were obtained by excluding variables in the final model until all remaining variables were associated with abnormal LFTs and liver fibrosis scores at the $p<0.05$ threshold. Once the final multivariate model had been established, variables that were

For peer review only - http://bmjopen.bmj.com/site/about/guidelines.xhtml

For peer review only

eliminated through this forward stepwise approach were added back to the model and were reported if associated at the p<0.05 threshold, to reduce the effects of residual confounding. Due to the low number of individuals with active HCV infection at the time of the study, we did not include this sub-group in univariate or multivariate analysis. These HCV RNA positive individuals have been described in more detail elsewhere (28). We present results of multivariate analysis in the form of Forrest plots generated using Microsoft Excel. A tabular form of the multivariate analysis containing the adjusted odds ratios (Adj. OR), and 95% CIs are included in the supplementary section of the manuscript.

Ethics

Ethics approval was provided by the Science and Ethics Committee of the Uganda Virus Research Institute (GC/127/12/11/06), the Ugandan National Council for Science and Technology (HS870), and the East of England-Cambridge South (formerly Cambridgeshire 4) NHS Research Ethics Committee UK (11/H0305/5). All participants provided written informed consent.

Patient and public involvement

Patients and the public were not involved in the design, conduct and reporting of the research.

RESULTS

Characteristics of study population

We analysed complete data for 8,099 participants (Suppl Table 3). Compared to females, there were more males who were HBV positive, (prevalence 3% vs 2%, respectively; p<0.001) and had consumed alcohol in the past 30 days, (40% vs 33%, respectively; p<0.001). More females were HIV positive (9% vs 6%, respectively; p<0.001). Males were more likely to be underweight (31% vs 16%), and females to be overweight (18% vs 5%); p<0.001 in both cases.

Proportion of population defined as having abnormal LFTs varies according to the reference range that is applied

The proportion of the population falling above the upper limit of normal (ULN) for each parameter is shown in Table 1, with ALT, AST and GGT distributions in Fig 1A-C (full data for all LFTs are shown in Suppl Fig 1). These results highlight the different burden of disease that can be estimated according to the reference range that is applied, with a higher proportion of the population falling above the ULN when the ARR was applied compared to the LRR (Fig 1A, B). Most striking, for AST, 13% of the population had a value that was deemed to be elevated based on ARR, compared to only

For peer review only - http://bmjopen.bmj.com/site/about/guidelines.xhtml

For peer review only

3% based on the LRR (Fig 1B). Using the ARR, ALT and BR were significantly more likely to be above the ULN in males than in females, and ALP was more likely to be higher in females ($p<0.001$ in each case, Table 1). These sex differences were not apparent when the LRR was applied. OR for deranged LFTs and fibrosis scores according to age and sex is shown in Suppl. fig 2.

The highest prevalence of liver fibrosis is predicted using the GPR score

We calculated APRI, FIB-4, GPR, RPR and S-index scores (Table 1). The estimated prevalence of fibrosis was highest when based on GPR score (23.5%; Fig 1D), compared to FIB-4 (5.3%), APRI (3.2%), S-index (3.9%) and RPR (0.1%). We excluded RPR scores from further statistical analysis because so few individuals were classified as having an elevated score (we therefore did not have statistical power to detect any factors associated with abnormal score). Because the APRI is derived using the ULN of AST, the proportion of the population classified as having a score consistent with liver fibrosis changes according to whether the ARR or LRR is used (Table 1). Based on previous validation among African individuals, there is evidence to suggest that GPR is the most accurate score for staging liver fibrosis (12); applying this approach, there is a prevalence of almost 1 in 4 adults with liver fibrosis in this population.

Evidence for the contribution of alcohol to liver disease

The prevalence of AST/ALT ratio >2, suggestive of alcoholic hepatitis, was 11% (888/8,099) (Fig 1E). There was a significant relationship between self-reported alcohol consumption and elevated AST/ALT ratio ($p<0.001$; Suppl Fig 3). However, 57% of participants with AST/ALT ratio >2 reported never having consumed alcohol (Fig 1E), possibly reflecting either under-reporting of alcohol use and/or other factors that underpin this pattern of LFTs. Self-reported alcohol consumption was associated with raised LFTs, as follows: ALT (Adj. OR 1.33, 95% CI 1.09,1.63) AST (Adj. OR 1.53, 95% CI 1.30, 1.78) GGT (Adj. OR 2.00 95% CI 1.69, 2.36), and with abnormal fibrosis scores, particularly GPR (Adj. OR 1.96, 95% CI 1.52, 2.54). All ORs, adjusted ORs, their respective 95% confidence intervals and p-values are shown in Table 2, and selected variables in Fig 2.

A raised GGT level in combination with AST/ALT ratio >2 can be used to increase the sensitivity of detection of alcoholic hepatitis (8). GGT levels were significantly higher among males with AST/ALT ratio ≥2 ($p<0.001$), but there was no relationship between GGT and AST/ALT ratio in females ($p=0.7$); Suppl Fig 4. This potentially indicates that alcohol is of more influence as a cause of an elevated AST/ALT ratio in men than in women. There was no significant association between AST/ALT ratio ≥2 and the presence of an elevated GPR score, predicting fibrosis ($p=0.2$; data not shown). We

For peer review only - http://bmjopen.bmj.com/site/about/guidelines.xhtml

calculated population attributable risk (PAR) as a way to assess the relative contribution of different risk factors to the overall burden of liver disease; Table 3. Overall, the most striking contribution arose from reported alcohol consumption, which accounted for 64% of abnormal S-index scores, 32% of elevated FIB-4 scores, and 19% of GPR abnormalities.

Abnormal LFTs and/or elevated fibrosis scores are associated with sex, age, and body mass index

Females were less likely to have high fibrosis scores based on FIB-4 compared to males (Adj. OR: 0.6), APRI (Adj. OR: 0.42), and S-Index (Adj. OR: 0.37) compared to males. FIB-4 score increased markedly with age: adults aged 40 – 49 (Adj. OR: 7.04), 50 – 59 (Adj. OR: 11.29), and adults >60 years (Adj. OR: 25.15) were more likely to have a higher FIB-4 than individuals < 39 years. Elevated BMI was associated only with a rise in GGT (Adj. OR: 1.47). However, being underweight was associated with a more pronounced pattern of liver derangement, including elevations in ALT (Adj. OR: 1.40), AST (Adj. OR: 1.44), GGT (Adj. OR: 1.37), abnormal fibrosis scores (APRI Adj. OR: 1.72,) and with raised AST/ALT ratio (Adj. OR: 1.61). 95% CI in each case are shown in Table 2.

Relationship between BBV infection and liver disease

HIV infection was associated with abnormal liver function tests, with significant OR for increased ALT, AST, ALP and GGT, as well as with raised GPR and S-index (on univariate and multivariate analysis; Table 2). HBV infection was significantly associated with a rise in hepatic transaminases (OR for raised ALT and AST 2.6 and 2.4 respectively, on multivariate analysis), and with liver fibrosis as measured by APRI and GPR (OR 3.6 and 4.2 respectively, on multivariate analysis). We investigated the prevalence of BBV infection among individuals with raised fibrosis scores. There was an association between the presence of HIV or HBV and raised GPR (p=0.005) and S-Index (p<0.001). Therefore, GPR and S-Index may be the most sensitive markers of inflammation and/or fibrosis in the context of HBV or HIV infection. HIV and HBV were associated with a lesser proportion of liver disease than alcohol based on calculation of PAR (Table 3), but still contributed to elevations in both LFTs and fibrosis scores. The OR for deranged LFTs/fibrosis scores in the context of HIV or HBV infection is shown in Fig 2.

Liver disease of unknown aetiology

Among individuals with GPR>0.32, 33.8% had either BBV infection or had AST/ALT>2 (suggesting potential alcoholic hepatitis) (Fig 1D; Suppl Fig 5). However, this illustrates that 66% have raised

For peer review only - http://bmjopen.bmj.com/site/about/guidelines.xhtml

fibrosis scores in the absence of a history of alcohol use, or HIV or HBV infection, suggesting that other factors unaccounted for in this study are likely to be contributing to the overall burden of liver disease. True prevalence of liver disease cannot be ascertained until reference ranges have been more carefully defined, correlating LFTs and fibrosis scores with the confirmed presence of underlying liver disease based on imaging or biopsy.

DISCUSSION

Liver disease is not well characterised in many parts of sSA despite the high prevalence of HIV and HBV, and potential exposure to hepatotoxins (1,3). In this study, we used cross-sectional data from a large population cohort to estimate the burden of liver disease and to assess the possible impact of BBV infection and alcohol consumption. The prevalence of abnormal LFTs depends on the reference range that is applied. The ARR suggests a higher prevalence of liver disease, therefore including more false-positives. The LRR was established based on individuals recruited from several countries across Africa (Rwanda, Uganda, Kenya, Zambia) (16). While the values were derived from purportedly healthy adults, it is impossible to rule out a high background prevalence of underlying liver disease; in defining higher values for the ULN of all tests, the LRR is more susceptible to false-negatives if used to screen for liver disease.

LFTs are a blunt tool for assessment of liver health, with many potential confounding factors. This current study only accounts for a limited range of aetiological agents, and we did not include other potentially relevant factors such as Schistosomiasis infection, exposure to aflatoxin and use of traditional medications. Furthermore, LFTs were measured at only one point in time, potentially overcalling liver disease as a result of transient abnormalities. Further studies will be required to investigate a greater range of risk factors, and to undertake longitudinal follow-up.

Composite fibrosis scores have been developed with the aim of improving sensitivity of detection of liver disease (29), but these scores also depend on platelet count which can be influenced by diverse factors. For example, in some African populations, thrombocytopenia is common due to infections such as malaria, schistosomiasis, HIV or endemic parasites, as well as being influenced by inflammatory conditions and certain drugs (9,10). We only had platelet counts for a sub-set of our study population, limiting the number for whom we could determine APRI, FIB-4, GPR, S-Index and RPR scores. Data surrounding the use of these scores in sSA is variable, but since in many low-income settings alternative diagnostic equipment is unavailable, non-invasive approaches are vital to

For peer review only - http://bmjopen.bmj.com/site/about/guidelines.xhtml

estimate liver damage and to stratify clinical management decisions.

APRI and FIB-4 are currently recommended by the World Health Organisation (WHO) for assessment of hepatic fibrosis in patients with chronic HBV or HCV infection (30,31). However, there is evidence showing that APRI is more accurate in assessing liver fibrosis among individuals with chronic HCV compared to HBV infection (11). GPR and S-Index have been validated in small studies in sSA, and have been associated with improved classification of liver fibrosis in chronic HBV infection when compared to APRI and FIB-4 (12–14). It is apparent that either larger studies, or indeed a meta-analysis, are required to further assess the accuracy of these tests in different populations. GPR and S-index may be worthwhile options to include in routine clinical practice to assess for liver fibrosis in African populations, given the high burden of HBV in this continent (32,33). RPR has been used to detect fibrosis among individuals with chronic HBV in China (26), however this score was excluded from our analysis due to a very small number of individuals falling above the suggested threshold for fibrosis.

The prevalence of AST/ALT ratio >2 in this population is 11%, suggesting potential alcoholic hepatitis (34), concordant with a previous study in Uganda in which 10% of the population was estimated to have alcoholic hepatitis (35), and with data from Uganda's non-communicable diseases risk factor survey which estimated that almost 10% of Ugandan adults have alcohol use disorders (36). Data from emergency attendances at Mulago Hospital in Kampala recorded 47% who reported alcohol use, while 21% and 10% met the study definitions of alcoholic misuse and alcoholic liver disease, respectively (35). Our data are based on self-reported alcohol consumption so may underestimate the true extent of alcohol use. We were unable to quantify alcohol intake or the nature of the alcohol consumed: this is challenging as alcohol is often home-brewed or home-distilled from locally grown grains or fruits, and the alcohol content may vary widely; e.g. the alcohol content of locally produced maize-based brews and liquor in Kenya ranged from 2%-7% and 18%-53%, respectively (36). The global challenge of morbidity and mortality associated with alcohol use is highlighted by recent studies from the Global Burden of Disease consortium, in which alcohol ranks as the seventh highest cause of DALYs and deaths and worldwide (2), and together with HBV infection is a leading aetiological agent of liver cancer (37).

Abnormal LFTs are common in HIV infection for diverse reasons including direct cytopathic effects of HIV on the hepatocytes, co-infection with other BBVs, opportunistic infection, malignancy, ART or other drugs, or secondary to other factors such as alcoholism (38–41). Although a proportion of our

For peer review only - http://bmjopen.bmj.com/site/about/guidelines.xhtml

study population with fibrosis were infected with BBV (21.6%) and/or had a history of alcohol consumption (12.2%), there was a residual proportion with scores suggestive of fibrosis and AST/ALT ratio >2 who cannot be accounted for through either alcohol or BBV infection. This implies that other factors contribute towards liver dysfunction in this population; a recently published article reported approximately 30% of liver cirrhosis in Africa are not attributed to HBV, HCV, or alcohol misuse and could be as a result of other understudied factors such as NAFLD and use of traditional medicine (35). Aflatoxin exposure is associated with liver cirrhosis and is among the major causes of hepatocellular carcinoma globally, with most cases reported from sSA. Within a previous study of the GPC, >90% of individuals had evidence of exposure (42–44).

In our population women were significantly more likely to be overweight women than men. This may be associated with a higher incidence of NAFLD in women. However, typically only mild rises in ALT are seen, and 80% of those with NAFLD have normal LFTs (45–47) so may not be identified within our current dataset. Diagnosis of NAFLD therefore depends on ultrasound scan (USS); previous studies have consistently shown 70-80% of obese patients have NAFLD on imaging (46,48,49). These imaging modalities were not available in our population, so we are unable to comment specifically on the possible prevalence of NAFLD. Interestingly, in this setting low body weight was more associated with deranged LFTs and with biochemical evidence of liver fibrosis, suggesting a range of pathology that may contribute to liver disease, including organ-specific effects of under-nutrition or stunting (37), as well as the effect of general systemic illness. Further studies are required to investigate the specific relationship between BMI and liver fibrosis in African populations.

In African populations, HCV infection has frequently been often over-reported due to a reliance on HCV-antibody (HCV-Ab) testing, which detects not only current infection but also previous exposure, and is known to be susceptible to false positive results (28). In this cohort, 298/8145 (3.7%) individuals tested HCV-Ab positive, but among these only 13 were HCV RNA positive (overall prevalence 13/8145 = 0.2%).

Appropriate reference ranges for LFTs are necessary to contribute to an understanding of the burden and aetiology of liver disease. Further work is required to determine appropriate thresholds for the ULN of different parameters in different settings in sSA, and to determine which fibrosis score is most specific, through application of a more widespread approach to elastography and/or other imaging. At present, we have identified alcohol, HIV and HBV as risk factors for deranged LFTs and liver fibrosis, with a striking contribution made by alcohol, but further investigation is needed to determine other risk

For peer review only

factors that contribute to liver disease in this setting.

DECLARATIONS:

CONSENT TO PUBLISH

All authors approve the publication of this manuscript

DATA SHARING STATEMENT

All data generated or analysed during this study are included in this published article (and its Supplementary Information files).

CONFLICT OF INTEREST

We have no conflicts of interest to declare.

FINANCIAL SUPPORT

The General Population Cohort is jointly funded by the UK Medical Research Council (MRC) and the UK Department for International Development (DFID) under the MRC/DFID Concordat agreement. The work on liver function also received additional funding from the MRC, (grant numbers G0801566 and G0901213-92157). JM is funded by a Leverhulme Mandela Rhodes Scholarship. PCM is funded by the Wellcome Trust, grant number 110110. LOD is funded by NIHR.

AUTHORS' CONTRIBUTIONS:

- Conceived the study : GAO, PCM, RN
- Data collection : AL, GA, RN
- Analysed the data : JM, JPH, PCM
- Wrote the manuscript : GAO, JM, JPH, LOD, PCM, RN
- Revised the manuscript : All authors

All authors have read and approved the manuscript

ACKNOWLEDGEMENTS

Nil

For peer review only

REFERENCES

1. Spearman CW, Sonderup MW. Health disparities in liver disease in sub-Saharan Africa. Vol. 35, Liver International. 2015. p. 2063–71.

2. Vento S, Dzudzor B, Cainelli F, Tachi K. Liver cirrhosis in sub-Saharan Africa: neglected, yet important. Lancet Glob Heal. 2018;6(10):e1060–1.

3. Price JC, Thio CL. Liver Disease in the HIV-Infected Individual. Clin Gastroenterol Hepatol. 2011;8(12):1002–12.

4. Hotez PJ, Kamath A. Neglected tropical diseases in sub-saharan Africa: review of their prevalence, distribution, and disease burden. PLoS Negl Trop Dis. 2009 A;3(8):e412.

5. Mitchell O, Feldman DM, Diakow M, Sigal SH. The pathophysiology of thrombocytopenia in chronic liver disease. Hepat Med. 2016;8:39–50.

6. Radcke S, Dillon JF, Murray AL. A systematic review of the prevalence of mildly abnormal liver function tests and associated health outcomes. Eur J Gastroenterol Hepatol. 2015;27(1):1–7.

7. Botros M, Sikaris KA. The de ritis ratio: the test of time. Clin Biochem Rev. 2013 34(3):117–30.

8. Limdi JK. Evaluation of abnormal liver function tests. Postgr Med J. 2003;79:307–12.

9. Huang R, Jia B, Wang G, Wu C. Non-invasive fibrosis markers for chronic hepatitis B in sub-Saharan Africa. Liver Int. 2017;37(11):1738–1738. A

10. Vaughan JL, Fourie J, Naidoo S, Subramony N, Wiggill T, Alli N. Prevalence and causes of thrombocytopenia in an academic state-sector laboratory in Soweto, Johannesburg, South Africa. S Afr Med J. 2015;105(3):215–9.

11. Yilmaz Y, Yonal O, Kurt R, Bayrak M, Aktas B, Ozdogan O. Noninvasive assessment of liver fibrosis with the aspartate transaminase to platelet ratio index (APRI): Usefulness in patients with chronic liver disease: APRI in chronic liver disease. Hepat Mon. 2011;11(2):103–6.

12. Lemoine M, Shimakawa Y, Nayagam S, Khalil M, Suso P, Lloyd J, et al. The gamma-glutamyl transpeptidase to platelet ratio (GPR) predicts significant liver fibrosis and cirrhosis in patients with chronic HBV infection in West Africa. Gut. 2016;65(8):1369–76.

13. Tag-Adeen M, Omar MZ, Abd-Elsalam FM, Hasaneen A, Mohamed MA, Elfeky HM, et al. Assessment of liver fibrosis in Egyptian chronic hepatitis B patients. Medicine (Baltimore). 2018;97(6):e9781.

14. Desalegn H, Aberra H, Berhe N, Gundersen SG, Johannessen A. Are non-invasive fibrosis markers for chronic hepatitis B reliable in sub-Saharan Africa? Liver Int. 2017 37(10):1461–7.

15. Mu R, Chen W, Pan B, Wang L, Hao X, Huang X, et al. First definition of reference intervals of liver function tests in China: a large-population-based multi-center study about healthy

For peer review only

adults. PLoS One. 2013;8(9):e72916.

16. Karita E, Ketter N, Price MA, Kayitenkore K, Kaleebu P, Nanvubya A, et al. CLSI-derived hematology and biochemistry reference intervals for healthy adults in eastern and southern Africa. PLoS One. 2009;4(2).

17. Melkie M, Yigeremu M, Nigussie P, Asrat S, Gebreegziabher T, Teka T, et al. Robust reference intervals for liver function test (LFT) analytes in newborns and infants. BMC Res Notes. 2012. 5:493.

18. Rustad P, Felding P, Franzson L, Kairisto V, Lahti A, Mårtensson A, et al. The Nordic Reference Interval Project 2000: recommended reference intervals for 25 common biochemical properties. Scand J Clin Lab Invest. 2004;64(4):271–84.

19. Neuschwander-Tetri BA, Unalp A, Creer MH, Nonalcoholic Steatohepatitis Clinical Research Network. Influence of local reference populations on upper limits of normal for serum alanine aminotransferase levels. Arch Intern Med. 2008;168(6):663–6.

20. Stevens W, Kamali A, Karita E, Anzala O, Sanders EJ, Jaoko W, et al. Baseline Morbidity in 2,990 Adult African Volunteers Recruited to Characterize Laboratory Reference Intervals for Future HIV Vaccine Clinical Trials. PLoS One. 2008;3(4):e2043.

21. Asiki G, Murphy GA V, Baisley K, Nsubuga RN, Karabarinde A, Newton R, et al. Prevalence of dyslipidaemia and associated risk factors in a rural population in South-Western Uganda: a community based survey. PLoS One. 2015;10(5):e0126166.

22. Murphy A, Av G, Rebecca N, Elizabeth H. Sociodemographic distribution of non-communicable disease risk factors in rural Uganda : a cross-sectional study . Int J Epidemiol 2013. 2014;42(6):1740–1753.

23. Stockdale AJ, Phillips RO, Geretti AM, HEPIK Study Group. The gamma-glutamyl transpeptidase to platelet ratio (GPR) shows poor correlation with transient elastography measurements of liver fibrosis in HIV-positive patients with chronic hepatitis B in West Africa. Response to: "The gamma-glutamyl transpeptidase to platelet ratio (GPR) predicts significant liver fibrosis and cirrhosis in patients with chronic HBV infection in West Africa" by Lemoine et al. Gut. 2016;65(5):882–4.

24. Lin Z-H, Xin Y-N, Dong Q-J, Wang Q, Jiang X-J, Zhan S-H, et al. Performance of the aspartate aminotransferase-to-platelet ratio index for the staging of hepatitis C-related fibrosis: An updated meta-analysis. Hepatology. 2011;53(3):726–36.

25. Sterling RK, Lissen E, Clumeck N, Sola R, Correa MC, Montaner J, et al. Development of a simple noninvasive index to predict significant fibrosis in patients with HIV/HCV coinfection. Hepatology. 2006;43(6):1317–25.

For peer review only

26. Chen B, Ye B, Zhang J, Ying L, Chen Y. RDW to platelet ratio: a novel noninvasive index for predicting hepatic fibrosis and cirrhosis in chronic hepatitis B. PLoS One. 2013;8(7):e68780.

27. Tag-Adeen M, Omar MZ, Abd-Elsalam FM, Hasaneen A, Mohamed MA, Elfeky HM, et al. Assessment of liver fibrosis in Egyptian chronic hepatitis B patients: A comparative study including 5 noninvasive indexes. Medicine (Baltimore). 2018 97(6):e9781.

28. Adland E, Jesuthasan G, Downs L, Wharton V, Wilde G, McNaughton AL, et al. Hepatitis virus (HCV) diagnosis and access to treatment in a UK cohort. BMC Infect Dis. 2018;18(1):461.

29. Yap CY, Choon Aw T. Liver Function Tests (LFTs). Vol. 19, Proceedings of Singapore Healthcare □. 2010

30. World health Organisation: Guidelines for the care and treatment of persons diagnosed with chronic hepatitis C virus infection. 2018. http://apps.who.int/iris/bitstream/handle/10665/273174/9789241550345-eng.pdf?ua=1

31. World health Organisation: Guidelines for the prevention, care and treatment of persons with chronic hepatitis B infection. 2015. http://apps.who.int/iris/bitstream/handle/10665/154590/9789241549059_eng.pdf?sequence=1

32. O'Hara GA, McNaughton AL, Maponga T, Jooste P, Ocama P, Chilengi R, et al. Hepatitis B virus infection as a neglected tropical disease. PLoS Negl Trop Dis. 2017;11(10):e0005842.

33. Lemoine M, Shimakawa Y, Njie R, Taal M, Ndow G, Chemin I, et al. Acceptability and feasibility of a screen-and-treat programme for hepatitis B virus infection in The Gambia: the Prevention of Liver Fibrosis and Cancer in Africa (PROLIFICA) study. Lancet Glob Heal. 2016;4(8):e559-67.

34. Hall P, Cash J. What is the real function of the liver "function" tests? Ulster Med J. 2012;81(1):30–6.

35. Opio CK, Seremba E, Ocama P, Lalitha R, Kagimu M, Lee WM. Diagnosis of alcohol misuse and alcoholic liver disease among patients in the medical emergency admission service of a large urban hospital in Sub-Saharan Africa; a cross sectional study. Pan Afr Med J. 2013;15:23.

36. Kabwama SN, Ndyanabangi S, Mutungi G, Wesonga R, Bahendeka SK, Guwatudde D. Alcohol use among adults in Uganda: findings from the countrywide non-communicable diseases risk factor cross-sectional survey. Glob Health Action. 2016;9(1):31302.

37. Akinyemiju T, Abera S, Ahmed M, Alam N, Alemayohu MA, Allen C, et al. The Burden of Primary Liver Cancer and Underlying Etiologies From 1990 to 2015 at the Global, Regional, and National Level. JAMA Oncol. 2017;3(12):1683.

38. Sterling RK, Chiu S, Snider K, Nixon D. The Prevalence and Risk Factors for Abnormal Liver Enzymes in HIV-Positive Patients without Hepatitis B or C Coinfections. Dig Dis Sci.

For peer review only - http://bmjopen.bmj.com/site/about/guidelines.xhtml

2008;53(5):1375–82.
39. Osakunor DNM, Obirikorang C, Fianu V, Asare I, Dakorah M. Hepatic Enzyme Alterations in HIV Patients on Antiretroviral Therapy: A Case-Control Study in a Hospital Setting in Ghana. PLoS One. 2015;10(8):e0134449.
40. Crane M, Iser D, Lewin SR. Human immunodeficiency virus infection and the liver. World J Hepatol. 2012;4(3):91.
41. Mata-Marín JA, Gaytán-Martínez J, Grados-Chavarría BH, Fuentes-Allen JL, Arroyo-Anduiza CI, Alfaro-Mejía A. Correlation between HIV viral load and aminotransferases as liver damage markers in HIV infected naive patients: A concordance cross-sectional study. Virol J. 2009;6:4–7.
42. Kuniholm MH, Lesi OA, Mendy M, Akano AO, Sam O, Hall AJ, et al. Aflatoxin exposure and viral hepatitis in the etiology of liver cirrhosis in the Gambia, West Africa. Environ Health Perspect. 2008;116(11):1553–7.
43. Kang M-S, Nkurunziza P, Muwanika R, Qian G, Tang L, Song X, et al. Longitudinal evaluation of aflatoxin exposure in two cohorts in south-western Uganda. Food Addit Contam Part A. 2015;32(8):1322–30.
44. Liu Y, Wu F. Global burden of aflatoxin-induced hepatocellular carcinoma: a risk assessment. Environ Health Perspect. 2010;118(6):818–24.
45. Almobarak AO, Barakat S, Khalifa MH, Elhoweris MH, Elhassan TM, Ahmed MH. Non alcoholic fatty liver disease (NAFLD) in a Sudanese population: What is the prevalence and risk factors? Arab J Gastroenterol. 2014;15(1):12–5.
46. Dyson JK, Anstee QM, McPherson S. Non-alcoholic fatty liver disease: a practical approach to diagnosis and staging. Frontline Gastroenterol. 2014 5(3):211–8.
47. Onyekwere CA, Ogbera AO, Balogun BO. Non-alcoholic fatty liver disease and the metabolic syndrome in an urban hospital serving an African community. Ann Hepatol.;10(2):119–24.
48. Argo CK, Caldwell SH. Epidemiology and Natural History of Non-Alcoholic Steatohepatitis. Clin Liver Dis. 2009;13(4):511–31.
49. Williams CD, Stengel J, Asike MI, Torres DM, Shaw J, Contreras M, et al. Prevalence of Nonalcoholic Fatty Liver Disease and Nonalcoholic Steatohepatitis Among a Largely Middle-Aged Population Utilizing Ultrasound and Liver Biopsy: A Prospective Study. Gastroenterology. 2011;140(1):124–31.

For peer review only

FIGURE LEGENDS

Fig 1: Liver function tests and hepatic fibrosis scores among adults in the Uganda General Population Cohort. Distribution of (A) ALT, (B) AST and (C) GGT. Dashed vertical lines indicate upper limit of normal (ULN) based on American reference range, ARR (blue) and local reference range, LRR (red), as shown in Suppl Table 2. Note no LRR for GGT. (D) Proportion of the population with an elevated GPR score, and among those with elevated GPR the proportion with a defined risk factor for fibrosis. (E) Proportion of the population with an elevated AST/ALT ratio, and among those with an elevated ratio the proportion with a self-reported history of alcohol intake.

Fig 2: Forrest plots to show odds ratio (OR) for host risk factors and elevated LFTs or fibrosis scores in the Uganda General Population Cohort. Data are presented for the final multivariate model for ALT, AST, APRI, GPR, and AST/ALT and we show variables that were independently associated with the outcome (statistically significant at the P<0.05 level after adjusting for other variables).

SUPPLEMENTARY DATA

All supporting data are accessible on-line via the following link: https://figshare.com/s/0b08de8a740991a7aa22 (this will be converted to a permanent DOI on acceptance of the paper).

Metadata table: raw data for 8145 adults in the Uganda General Population cohort (available as .xls and .csv files)

Supporting data file (pdf file) contains the following tables and figures:

Suppl Table 1: Origin, reference ranges and clinical significance of liver functions tests (LFTs)

Suppl Table 2: Scores to estimate liver fibrosis, calculated from liver function tests

Suppl Table 3: Description of characteristics of study participants with liver function test (LFT) results from the Ugandan General Population Cohort (N=8,099)

For peer review only - http://bmjopen.bmj.com/site/about/guidelines.xhtml

Suppl Fig 1: Distribution of liver function tests in Uganda General Population Cohort. Dashed vertical lines indicate upper limit of normal (ULN) based on American reference range, ARR (orange line is the ULN for female; blue line is the ULN for males) and local reference range, LRR (black), as shown in Suppl Table 1. Note no LRR for GGT. ULN for bilirubin using ARR is the same for both male and female, indicated by red dashed line. Data are shown for study participants aged ≥16 years, apart from ALP which is shown for participants aged ≥20 to exclude teenagers who may have elevated ALP as a normal physiological consequence of bone growth.

Suppl Fig 2: Odds ratio for deranged ALT, AST, APRI, GPR and AST/ALT among participants grouped by sex and age.

Suppl Fig 3: **Proportion of Uganda General Population cohort reporting alcohol consumption among individuals with and without AST/ALT ratio >2**

Suppl Fig 4: Proportion of Uganda General Population Cohort with elevated GGT, according to AST/ALT ratio. (A) males, with upper limit of normal GGT=61 (B) females, with upper limit of normal GGT=36. P-values by Fisher's Exact Test.

Suppl Fig 5: Proportion of Uganda General Population Cohort with blood borne virus (BBV) infection, according to GPR score. P-value by Fisher's Exact Test, showing significant enrichment of BBV infection among individuals with elevated GPR score >0.32.

For peer review only

TABLES

Table 1: Study participants from the Uganda General Population Cohort with abnormal LFT results and fibrosis scores based on upper limit of normal according to American reference range (ARR) and local reference ranges (LRR).

Enzyme Type	Total n / N (%)	Male n / N (%)	Female n / N (%)	p value[1]
ALT[2]				
Abnormal ARR*	573 / 8,099 (7.1)	162 / 3,542 (4.6)	411 / 4,557 (9.0)	<0.001
Abnormal LRR**	209 / 8,099 (2.6)	87 / 3,542 (2.5)	122 / 4,557 (2.7)	0.53
AST[2]				
Abnormal ARR*	1,011 / 8,099 (12.5)	434 / 3,542 (12.3)	577 / 4,557 (12.7)	0.58
Abnormal LRR**	241 / 8,099 (3.0)	123 / 3,542 (3.5)	118 / 4,557 (2.6)	0.02
GGT[2,3]				
Abnormal ARR*	889 / 8,099 (11.0)	362 / 3,542 (10.2)	527 / 4,557 (11.6)	0.06
BR[2]				
Abnormal ARR*	1,051 / 8,099 (13.0)	635 / 3,542 (18.0)	416 / 4,557 (9.1)	<0.001
Abnormal LRR**	497 / 8,099 (6.1)	214 / 3,542 (6.0)	283 / 4,557 (6.2)	0.75
ALP[2,4]				
Abnormal ARR*	1,161 / 5,616 (20.7)	315 / 2,273 (13.9)	846 / 3,343 (25.3)	<0.001
Abnormal LRR**	139 / 5,616 (2.5)	60 / 2,273 (2.6)	79 / 2,273 (2.4)	0.513
FIB-4[2]				
Abnormal***	99 / 1,877 (5.3)	54 / 824 (6.6)	45 / 1,053 (4.3)	0.03
APRI[2,5]				
Abnormal ARR*,***	145 / 1,877 (7.7)	95 / 824 (11.5)	50 / 1,053 (4.8)	<0.001
Abnormal LRR*, ***	60 / 1,877 (3.2)	42 / 824 (5.1)	18 / 1,053 (1.7)	<0.001
GPR[2]				
Abnormal***	441 / 1,877 (23.5)	185 / 824 (22.5)	256 / 1,053 (24.3)	0.35
AST/ALT[2]				
Abnormal***	882 / 8,099 (10.9)	420 / 3,542 (11.9)	462 / 4,557 (10.1)	0.01
S-Index[2]				
Abnormal***	73 / 1,877 (3.9)	50 / 824 (6.1)	23 / 1,053 (2.2)	<0.001

[1] p-value calculated to determine whether significant difference between males and females in each category using chi-square test. [2] ALT - Alanine Transminase, AST - Aspartate Transminase, GGT - Gamma-glutamyl transpeptidase, ALP - Alkaline Phosphatase, BR - Total Bilirubin, FIB-4 - fibrosis 4, APRI - AST to Platelet Ratio Index, GPR - GGT to platelet ratio, AST/ALT ratio - Aspartate/ Alanine ratio. [3] LRR for GGT not defined. [4] Individuals under the age of 19 were excluded.[5] APRI score calculated using ULN of AST using both the ARR and LRR.
* Abnormal LFTs, according to ARR, are defined as test results outside of the following ranges: ALT (Male: 10 – 55 U/L, Female: 7 – 30 U/L), AST (Male: 10 – 40 U/L, Female: 9 – 32 U/L), GGT (Male: 8 – 61 U/L, Female: 5 – 36 U/L), BR (0 – 17 mmol/L), ALP (Male: 45 – 115 U/L, Female: 30 – 100 U/L). ** Abnormal LFTs, according to LRR, are defined as test results outside of the following ranges: ALT (8 – 61 U/L), AST (14 – 60 U/L), BR (2.9 – 37 mmol/L), ALP (48 – 164 U/L). *** Threshold used to predict liver fibrosis: APRI > 0.7. FIB-4 >3.25. GPR >0.32. RPR >0.825. S-Index >0.3

For peer review only - http://bmjopen.bmj.com/site/about/guidelines.xhtml

Table 2: Univariate and multivariate analysis for factors associated with abnormal liver function tests according to American reference ranges (ARR) for ALT, AST, ALP, GGT, and TB, and laboratory markers of fibrosis in adults in the Uganda General Population Cohort.

	ALT [1,6] OR (95% CI)	*AST [1,6] OR (95% CI)*	*ALP [1,4,6,] OR (95% CI)*	*GGT [1,6] OR (95% CI)*	*TB [1,6] OR (95% CI)*	*FIB-4 [1,7] OR (95% CI)*	*APRI [1,7,#] OR (95% CI)*	*GPR [1,7] OR (95% CI)*	*AST/ALT [1,7] OR (95% CI)*	*S-Index [3,7] OR (95% CI)*
UNIVARIATE ANALYSIS										
Sex										
Male	*Ref*	*Ref*	*Ref*	*Ref*	*Ref*	*Ref*	*Ref*	*Ref*	*Ref*	*Ref*
Female	2.06 (1.71,2.49)***	1.04 (0.91,1.18)^{ns}	0.93 (0.84,1.01)^{ns}	1.15 (1.00,1.32)*	0.46 (0.20,0.24)***	0.64(0.42,0.96)*	0.38 (0.27,0.55)***	1.10 (0.89,1.38)^{ns}	0.84 (0.73,0.96)*	0.35 (0.21,0.57)***
Age										
<19	*Ref*	*Ref*	-	*Ref*	*Ref*	*Ref* [5]	*Ref*	*Ref*	*Ref*	*Ref* [5]
20-29	1.33 (1.03,1.73)*	0.9 (0.73,1.11)^{ns}	*Ref* [4]	2.61 (1.92,3.56)***	1.46 (1.22,1.75)***		2.57 (1.41,4.71)**	2.63 (1.72,4.03)***	0.55 (0.43,0.70)***	
30-39	1.58 (1.22,2.04)***	1.17 (0.95,1.43)^{ns}	0.72 (0.60,087)***	6.59 (5.00,8.72)***	1.15 (0.94,1.39)^{ns}		3.15 (1.76,5.68)***	6.22 (4.21,9.18)***	0.67 (0.53,0.85)**	
40-49	1.41 (1.04,1.87)*	1.47 (1.12,1.80)***	0.48 (0.38,0.59)***	8.34 (6.29,11.07)***	1.02 (0.83,1.27)^{ns}	8.48 (3.95,18.18)***	4.00 (2.22,7.18)***	7.63 (5.12,11.36)***	0.83 (0.65,1.05)^{ns}	5.02 (2.79,9.68)***
50-59	1.38 (1.00,1.90)*	1.57 (1.25,2.00)***	0.82 (0.66,1.02)^{ns}	8.03 (5.93,10.86)***	0.92 (0.71,1.18)^{ns}	14.60 (9.86,31.03)***	3.50 (1.80,6.73)***	9.10 (5.91,14.0)***	1.11 (0.86,1.43)^{ns}	4.71 (2.31,9.59)***
>60	1.39 (1.03,1.88)*	1.24 (0.98,1.55)^{ns}	1.28 (1.06,1.54)**	6.84 (5.09,9.20)***	0.56 (0.42,0.74)***	34.88 (17.80,68.39)***	3.68 (2.00,7.00)***	8.20 (5.42,12.41)***	2.23 (1.82,2.72)***	5.43 (2.84,10.39)***
Alcohol										
No	*Ref*	*Ref*	*Ref*	*Ref*	*Ref*	*Ref*	*Ref*	*Ref*	*Ref*	*Ref*
Yes	1.41 (1.16,1.70)***	1.57 (1.35,1.83)***	1.0 (0.86,1.13)***	2.14 (1.83,2.51)***	0.99 (0.85,1.15)^{ns}	2.02 (1.22,3.32)**	1.60 (1.04,2.31)*	2.10 (1.61,2.66)***	1.28 (1.08,1.50)**	6.09 (3.16,11.72)***
BMI [2]										
Normal	*Ref*	*Ref*	*Ref*	*Ref*	*Ref*	*Ref*	*Ref*	*Ref*	*Ref*	*Ref*
Underweight	1.41 (1.12,1.77)**	1.45 (1.23,1.71)***	1.17 (0.96,1.44)^{ns}	1.42 (1.16,1.73)**	0.69 (0.57,0.83)***	1.78 (1.06,3.00)^{ns}	1.78 (1.10,2.60)*	1.07 (0.78,1.50)^{ns}	1.62 (1.37,1.92)***	1.87 (1.04,3.33)*
Overweight	1.10 (0.85,1.41)^{ns}	0.73 (0.58,0.92)**	0.93 (0.77,1.13)^{ns}	1.36 (1.11,1.66)**	0.75 (0.59,0.95)*	0.74 (0.35,1.56)^{ns}	0.91(0.50,1.65)^{ns}	1.15 (0.82,1.60)^{ns}	0.57 (0.42,0.76)***	0.87 (0.38,2.03)^{ns}
HIV status										
Negative	*Ref*	*Ref*	*Ref*	*Ref*	*Ref*	*Ref*	*Ref*	*Ref*	*Ref*	*Ref*
Positive	1.63 (1.24,2.15)***	2.30 (1.87,2.83)***	1.47 (1.19,1.81)***	4.83 (3.98,5.85)***	0.21 (0.14,0.33)***	0.28 (0.07,1.20)^{ns}	1.30 (0.68,2.30)^{ns}	3.88 (2.62,5.73)***	1.06 (0.80,1.42)^{ns}	4.00 (2.08,7.69)***
HBV status										
Negative	*Ref*	*Ref*	*Ref*	*Ref*	*Ref*	*Ref*	*Ref*	*Ref*	*Ref*	*Ref*
Positive	2.61 (1.77,3.84)***	2.52 (1.84,3.44)***	1.07 (0.72,1.60)^{ns}	1.80 (1.24,2.60)***	1.10 (0.76,1.60)^{ns}	2.01 (0.62,6.50)^{ns}	3.56 (1.80,7.10)***	4.24 (2.27,7.93)***	0.98 (0.63,0.15)^{ns}	4.92 (2.07,11.69)***

For peer review only - http://bmjopen.bmj.com/site/about/guidelines.xhtml

MULTIVARIATE ANALYSIS										
Sex										
Male	*Ref*	*Ref*	*Ref*	*Ref*	*Ref*	*Ref*	*Ref*	*Ref*	*Ref*	*Ref*
Female	2.30 (1.89,2.81)***	1.20 (1.04,1.38)*	2.11 (1.83,2.44)***	1.01 (0.86,1.19)^{ns}	0.46 (0.40,0.53)***	0.62 (0.40,0.97)*	0.42 (0.30,0.62)***	1.11 (0.87,1.41)^{ns}	0.90 (0.78,1.06)^{ns}	0.37 (0.22,0.63)***
Age										
<19	*Ref*	*Ref*	-	*Ref*	*Ref*		*Ref*	*Ref*	*Ref*	
20-29	1.26 (0.95,1.68)^{ns}	0.89 (0.70,1.12)^{ns}	*Ref*[4]	1.69 (1.19,2.41)**	1.52 (1.25,1.84)***	*Ref*[5]	3.22 (1.66,6.22)**	1.86 (1.19,2.92)**	0.57 (0.44,0.75)***	*Ref*[5]
30-39	1.35 (1.00,1.80)*	1.00 (0.79,1.27)^{ns}	0.68 (0.56,0.82)***	3.96 (2.87,5.46)***	1.29 (1.02,1.59)*		3.55 (1.81,7.00)***	3.70 (2.43,5.66)***	0.72 (0.55,0.95)*	
40-49	1.13 (0.83,1.56)^{ns}	1.20 (0.95,1.52)^{ns}	0.46 (0.37,0.57)***	4.87 (3.54,6.70)***	1.17 (0.94,1.47)^{ns}	7.04 (3.19,15.52)***	4.00 (2.04,7.82)***	4.45 (2.88,6.87)***	0.93 (0.71,1.21)^{ns}	2.68 (1.37,5.26)**
50-59	1.09 (0.77,1.55)^{ns}	1.29 (0.99,1.67)^{ns}	0.82 (0.66,1.02)^{ns}	5.02 (3.58,7.02)***	1.01 (0.78,1.32)^{ns}	11.29 (5.13,24.80)***	3.45 (1.65,7.22)**	5.75 (3.61,9.15)***	1.22 (0.92,1.61)^{ns}	2.76 (1.29,5.90)**
>60	1.13 (0.81,1.57)^{ns}	1.00 (0.78,1.30)^{ns}	1.32 (1.09,1.59)**	4.98 (3.59,6.90)***	0.60 (0.45,0.80)***	25.15 (12.32,51.35)***	3.50 (1.73,7.11)**	5.39 (3.42,8.47)***	2.20 (1.74,2.77)***	3.34 (1.63,6.84)**
Alcohol										
No	*Ref*	*Ref*	-	*Ref*	-	*Ref*	*Ref*	*Ref*	*Ref*	*Ref*
Yes	1.33 (1.09,1.63)**	1.53 (1.30,1.78)***	-	2.00 (1.69,2.36)***	-	2.05 (1.24,3.40)**	1.51 (1.00,2.27)*	1.96 (1.52,2.54)***	1.26 (1.06,1.50)**	5.23 (2.72,10.04)***
BMI[2]										
Normal	*Ref*	*Ref*	-	*Ref*	*Ref*	-	*Ref*	-	*Ref*	-
Underweight	1.40 (1.11,1.75)**	1.44 (1.21,1.70)***	-	1.37 (1.11,1.68)**	0.70 (0.58,0.83)***	-	1.72 (1.11,2.65)*	-	1.61 (1.36,1.91)***	-
Overweight	1.12 (0.87,1.44)^{ns}	0.75 (0.60,0.95)*	-	1.47 (1.19,1.82)***	0.72 (0.57,0.92)**	-	0.95 (0.52,1.73)^{ns}	-	0.56 (0.42,0.76)***	-
HIV status										
Negative	*Ref*	*Ref*	*Ref*	*Ref*	*Ref*	-	-	*Ref*	-	*Ref*
Positive	1.59 (1.20,2.10)***	2.13 (1.72,2.63)***	1.47 (1.19,1.81)***	4.76 (3.89,5.82)***	0.22 (0.14,0.34)***	-	-	3.84 (2.58,5.70)***	-	3.58 (1.84,6.94)***
HBV status										
Negative	*Ref*	*Ref*	-	*Ref*	-	-	*Ref*	*Ref*	-	*Ref*
Positive	2.61 (1.76,3.86)***	2.40 (1.74,3.31)***	-	1.65 (1.11,2.45)*	-	-	3.60 (1.79,7.27)***	4.26 (2.23,8.12)***	-	4.37 (1.80,10.58)***

[1] ALT - Alanine Transminase, AST - Aspartate Transminase, GGT - Gamma-glutamyl transpeptidase, ALP - Alkaline Phosphatase, BR -Total Bilirubin, FIB-4 - fibrosis 4, APRI - AST to Platelet Ratio Index, GPR - GGT to plate Alanine ratio. OR - odds ratio.
[2] Body Mass Index (BMI) Classification according to WHO (weight/height^2: kg/m^2): Underweight (<18.5 kg/m^2), Normal weight (18.5 – 24.99 kg/m^2), Overweight (25.0 – 29.99 kg/m^2), Obese (>30.0 kg/m^2)
[3] An S-index score of >0.3 is suggestive of liver fibrosis
[4] Individuals under the age of 19 were excluded. Reference age group is 20 – 29
[5] Reference age group consists of all individuals under the age of 39
[6] Abnormal LFTs, according to ARR, are defined as test results outside of the following ranges: ALT (Male: 10 – 55 U/L, Female: 7 – 30 U/L), AST (Male: 10 – 40 U/L, Female: 9 – 32 U/L), GGT (Male: 8 – 61 U/L, Female: 5 – 36 U/L), BR (0 – 17 mmol/L), ALP (Male: 45 – 115 U/L, Female: 30 – 100 U/L)
[7] Threshold used to predict liver fibrosis: APRI > 0.7. FIB-4 >3.25. GPR >0.32. RPR >0.825. S-Index >0.3
[#] APRI score calculated using ULN of AST using African reference range
Significance level: * = (p<0.05), ** = (p<0.01), *** = (p<0.001), *ns* = (p>0.05)

For peer review only - http://bmjopen.bmj.com/site/about/guidelines.xhtml

For peer review only

For peer review only - http://bmjopen.bmj.com/site/about/guidelines.xhtml

Table 3: Relative risk, population attributable risk (PAR) percent, and the number of individuals with abnormal liver function tests in the Uganda General Population Cohort. Analysis according to American reference ranges (ARR for ALT, AST, ALP, GGT, and TB)

Variable	ALT [1,3]	AST [1,3]	ALP [1,3]	GGT [1,3]	TB [1,3]	FIB-4 [1,4]	APRI [1,4,#]	GPR [1,4]	AST/ ALT [1,4]	S-Index [2,4]
Alcohol+										
Abnormal Result n (%)	248 (8.5)	467 (16.0)	533 (19.6)	555 (19)	381 (13.1)	72 (11.0)	80 (12.25)	260 (39.8)	379 (13.0)	60 (9.2)
RR (95% CI)[1]	1.4 (1.2 – 1.6)	1.5 (1.4 – 1.7)	1.2 (0.9 – 1.7)	2.9 (2.6 – 3.4)	1.0 (0.9 – 1.1)	5.0 (3.2 – 7.7)	2.3 (1.7 – 3.2)	2.7 (2.3 – 3.2)	1.3 (1.2 – 1.5)	8.7 (4.8 – 15.6)
PAR (%)[1,6]	11.3%	15.9%	0.6%	41.3%	0.3%	58.2%	31.3%	37.1%	10.8%	72.7%
Adj. PAR (%)[5,6]	10.0%	13.9%	-2.6%	26.7%	1.0%	32.4%	16.2%	19.4%	8.0%	64.0%
HIV+										
Abnormal Result n (%)	71 (11.7)	144 (23.7)	142 (24.8)	227 (37.3)	21 (3.5)	2 (1.6)	14 (11.0)	73 (57.5)	59 (9.7)	15 (11.8)
RR (95% CI)[1]	1.7 (1.4 – 2.2)	2.0 (1.8 – 2.4)	1.2 (1.1 – 1.4)	4.2 (3.7 – 4.8)	0.3 (0.2 – 0.4)	0.3 (0.1– 1.1)	1.5 (0.9 – 2.5)	2.7 (2.3 – 3.3)	0.9 (0.7 – 1.1)	3.6 (2.1 – 6.1)
PAR (%)[1,6]	5.3%	7.3%	2.2%	19.5%	-6.0%	-5.09%	3.1%	10.5%	-0.9%	14.7%
Adj. PAR (%)[5,6]	4.3%	6.5%	1.1%	17.6%	-6.0%	-4.6%	1.4%	8.3%	-0.1%	13.6%
HBV+										
Abnormal Result n (%)	33 (15.0)	56 (25.4)	32 (19.5)	39 (17.7)	35 (16)	4 (8.2)	13 (26.53)	25 (51.0)	22 (10.0)	8 (16.3)
RR (95% CI)[1]	2.2 (1.6 – 3.0)	2.1 (1.7 – 2.7)	0.9 (0.7 – 1.3)	1.6 (1.2 – 2.2)	1.2 (0.9 – 1.7)	1.6 (0.6 – 4.1)	1.5 (0.9 – 2.5)	2.2 (1.7 – 3.0)	0.9 (0.6 – 1.4)	4.6 (2.3 – 9.0)
PAR (%)[1,6]	3.1%	2.9%	-0.2%	1.7%	0.6%	1.5%	3.1%	3.1%	-0.2%	8.6%
Adj. PAR (%)[5,6]	3.3%	2.8%	0.02%	1.4%	0.2%	1.4%	5.7%	2.9%	-0.3%	7.6%

[1] ALT - Alanine Transminase, AST - Aspartate Transminase, GGT - Gamma-glutamyl transpeptidase, ALP - Alkaline Phosphatase, BR - Total Bilirubin, FIB-4 - fibrosis 4, APRI - AST to Platelet Ratio Index, GPR - GGT to platelet ratio, AST/ALT ratio - Aspartate/ Alanine ratio, RR - relative risk, PAR (%) - population attributable risk percent, 95% CI denotes 95% confidence interval

[2] An S-index score of >0.3 is suggestive of liver fibrosis

[3] Abnormal LFTs, according to ARR, are defined as test results outside of the following ranges: ALT (Male: 10 – 55 U/L, Female: 7 – 30 U/L), AST (Male: 10 – 40 U/L, Female: 9 – 32 U/L), GGT (Male: 8 – 61 U/L, Female: 5 – 36 U/L), BR (0 – 17 mmol/L), ALP (Male: 45 – 115 U/L, Female: 30 – 100 U/L)

[4] Threshold used to predict liver fibrosis: APRI > 0.7. FIB-4 >3.25. GPR >0.32. RPR >0.825. S-Index >0.3

[5] Adjusted for age, sex, alcohol consumption, HBV diagnosis, HIV status, and Body Mass Index.

[6] A measure of zero indicates of no association between the risk factor and abnormal liver function tests. A positive value indicates that the exposure to the risk factor is a risk factor, while a negative value indicates that it is a protective factor.

[#] APRI score calculated using ULN of AST using African reference range

+ number of abnormal result, RR and PAR (%) are based on individuals who were classified as positives within each variable (ie. Alcohol drinkers, HIV positive, HBV positive)

For peer review only

For peer review only - http://bmjopen.bmj.com/site/about/guidelines.xhtml

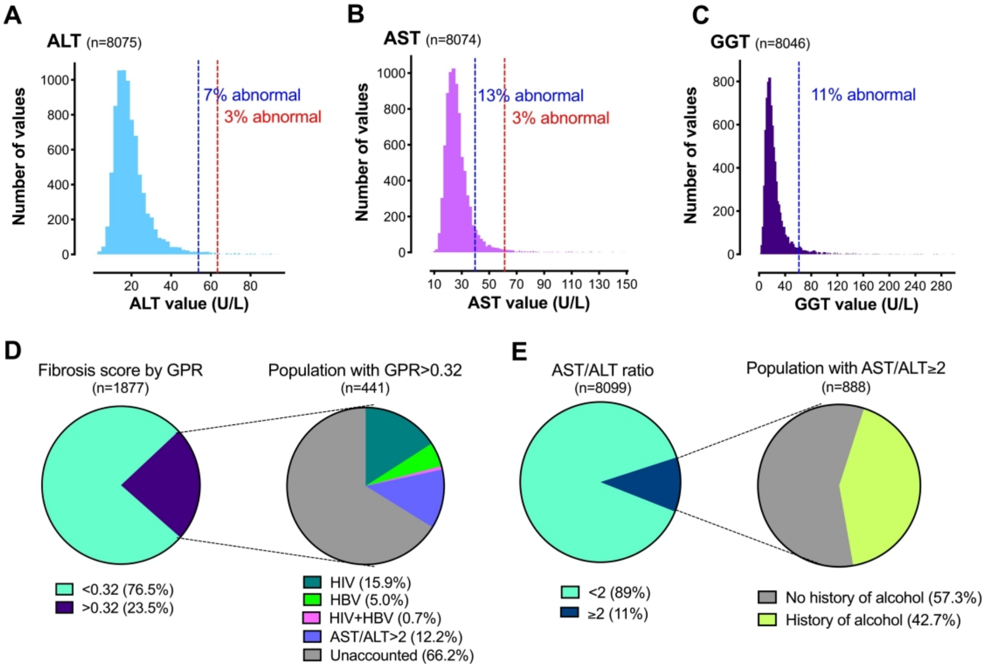

291x198mm (300 x 300 DPI)

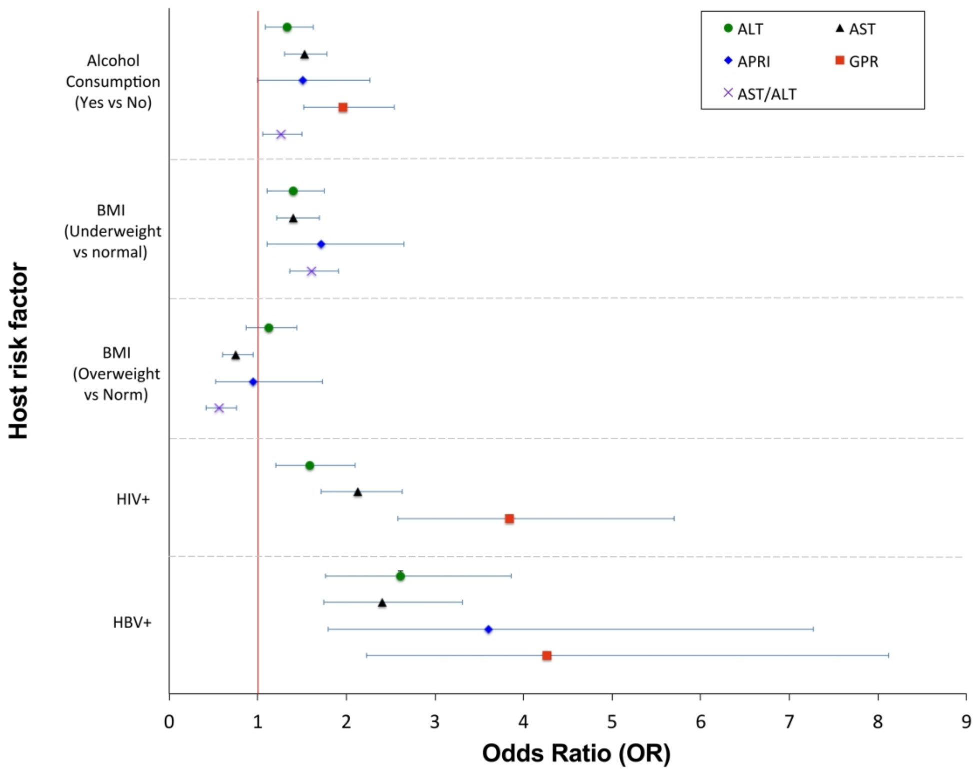

226x180mm (300 x 300 DPI)

SUPPLEMENTARY MATERIAL

This material is available on-line at https://figshare.com/s/0b08de8a740991a7aa22
On acceptance for publication, this will be made publicly available using a DOI

Liver function tests and fibrosis scores in a rural population in Africa: a cross-sectional study to estimate the burden of disease and associated risk factors

CONTENTS

Suppl Table 1: Origin, reference ranges and clinical significance of liver function tests (LFTs)

Suppl Table 2: Scores to estimate liver fibrosis, calculated from liver function tests

Suppl Table 3: Description of characteristics of study participants with liver function test (LFT) results from the Ugandan General Population Cohort (N=8,099)

Suppl Fig 1: Distribution of liver function tests in Uganda General Population Cohort.

Suppl Fig 2: Odds ratio for deranged ALT, AST, APRI, GPR and AST/ALT among participants grouped by sex and age.

Suppl Fig 3: Proportion of Uganda General Population cohort reporting alcohol consumption among individuals with and without AST/ALT ratio >2

Suppl Fig 4: Proportion of Uganda General Population Cohort with elevated GGT, according to AST/ALT ratio. (A) males, with upper limit of normal GGT=61 (B) females, with upper limit of normal GGT=36. P-values by Fisher's Exact Test

Suppl Fig 5: Proportion of Uganda General Population Cohort with blood borne virus (BBV) infection, according to GPR score. P-value by Fisher's Exact Test, showing significant enrichment of BBV infection among individuals with elevated GPR score >0.32.

For peer review only

Suppl data Table 1: Origin, reference ranges and clinical significance of liver function tests (LFTs) identified from published literature (7,10,54). LRR: local reference range (derived from populations in Africa); ARR: American reference range.

Biomarker	Origin	LRR	ARR	Common causes of derangement (Abnormal elevation for all markers other than albumin)
Alanine transferase (ALT)	Highest concentration in hepatocytes (small amounts in other tissues: muscles, adipose tissues, intestines, colon, prostate, and brain)	8 – 61*U*/L	Male: 10 - 55 *U*/L<br>Female: 7 - 30 *U*/L	• Acute / chronic viral hepatitis (EBV/CMV/HBV/HCV/HEV)<br>• Alcoholism<br>• Non-alcoholic fatty liver disease (NAFLD)<br>• Drugs: antipsychotics, antibiotics, statins.<br>• Autoimmune hepatitis<br>• Ischaemic liver damage<br>• Haemochromatosis<br>• Wilson's disease<br>• Coeliac disease
Aspartate transferase (AST)	Hepatocytes<br>Cardiac muscle<br>Skeletal muscle	14 - 60 *U*/L	Male: 10 - 40 *U*/L<br>Female: 9 - 32 *U*/L	• The causes listed for raised ALT.<br>• As AST is abundant in skeletal, cardiac and smooth muscle it may also be elevated in patients with cardiac disease, myositis or muscular dystrophy.
Alkaline phosphatase (ALP)	Liver (from biliary epithelium)<br>Bone<br>Placenta	48 - 164 *U*/L	Male: 45 - 115 *U*/L<br>Female: 30–100 *U*/L	• Bile duct obstruction<br>• Primary biliary cirrhosis<br>• Primary sclerosing cholangitis<br>• Drugs: Antibiotics, antiepileptics, MAOI's<br>• Bone growth, and bone disease<br>• Pregnancy<br>• Hepatic congestion from right sided heart failure
Gamma-glutamyl-transferase (GGT)	Liver<br>Kidney<br>Pancreas<br>Intestine<br>Prostate	Nil availabl e	Male: 8 - 61 *U*/L<br>Female: 5 - 36 *U*/L	• Obesity<br>• Hepatobiliary disease<br>• Pancreatic disease<br>• Alcoholism<br>• Drugs: carbamazepine, phenytoin, and barbituates.
Bilirubin (BR)	Red blood cells<br>Liver<br>Bone marrow	2.9 – 37.0 *m*mol/L	0 – 17 *m*mol/L	**Unconjugated hyperbilirubinaemia**<br>• Haemolysis (sickle cell disease and malaria particularly relevant)<br>• Ineffective erythropoiesis<br>• Gilbert's syndrome<br>• Drugs: Rifampicin<br>**Conjugated hyperbilirubinaemia**<br>• Liver disease<br>• Biliary obstruction

For peer review only - http://bmjopen.bmj.com/site/about/guidelines.xhtml

Albumin (Alb)	Liver; acute phase marker.	35 – 52 g/L	35 – 55 g/L	Lowered in association with:<br>• Chronic liver disease.<br>• Nephrotic syndrome,<br>• Protein losing enteropathy,<br>• Protein Energy Malnutrition<br>• Hypercatabolic states, e.g. in association with malignancy, infection.<br>• Congestive cardiac failure

LRR: Local Reference Ranges derived from a study by Karita et al (19). All ranges are for both male and female.
ARR: American Reference Ranges derived from MGH Clinical Laboratories.
MAOI: Monoamine oxidase inhibitors
*No local references were available for Gamma GT
[4]Bilirubin measurement is total Bilirubin concentration measured in *m*mol/L

For peer review only

Suppl Table 2: Scores to estimate liver fibrosis, calculated from liver function tests

Score	Formula	Threshold used to predict fibrosis	Sensitivity and specificity of fibrosis threshold (derived from previous studies)
APRI	(AST/ULN AST*100) / platelet count	0.7	Sensitivity: 77%<br>Specificity: 72%<br>Derived from meta-analysis of studies of HCV infection (26).
FIB-4	(Age in years*AST) / (platelet count * √ALT level)	3.25	Specificity: 97%<br>Positive predictive value: 65%<br>Derived from HIV/HCV coinfected individuals (27).
GPR	(GGT/ULN of GGT/platelet count) ×100	0.32	Optimal cut-off value for predicting significant fibrosis.<br>Derived from individuals with chronic HBV infection in The Gambia (14).
RPR	Red cell distribution width%/ platelet count	0.825	Sensitivity: 63.1%<br>Specificity: 85.5%<br>Positive predictive value: 65%<br>Derived from individuals with chronic HBV infection in China (28).
S-index	(1000×GGT)÷(platelet count ×Albumin^2)	0.3	Specificity: 94%<br>Positive predictive value: 87%<br>Accuracy: 68%<br>Derived from individuals with chronic HBV infection in Egypt (29).

AST = Aspartate transaminase at u/l, ULN = upper limit of normal,
ALT = Alanine transaminase at u/l
GGT= Glutamyltansferase at u/l, ULN = upper limit of normal,
Platelet count at 10^9/L

For peer review only - http://bmjopen.bmj.com/site/about/guidelines.xhtml

Suppl Table 3: Description of characteristics of study participants with liver function test (LFT) results from the Ugandan General Population Cohort (N=8,099)

Variable	*Total n(%)*	*Male n(%)*	*Female n(%)*	*p value[1]*
	8,099 (100.00)	3,542 (100.00)	4,557 (100.00)	
Age Group				
16-19	2,481 (30.6)	1,268 (35.8)	1,213 (26.6)	<0.001
20-29	1,508 (18.6)	618 (17.5)	890 (19.5)	0.02
30-39	1,349 (16.6)	510 (14.4)	839 (18.4)	<0.001
40-49	1,095 (13.5)	454 (12.8)	641 (14.0)	0.10
50-59	744 (9.2)	315 (8.9)	429 (9.4)	0.42
>60	922 (11.4)	377 (10.8)	545 (12.0)	0.06
Max Education				
None	759 (9.4)	208 (5.9)	551 (12.1)	<0.001
Primary	5,165 (63.8)	2,380 (67.2)	2,785 (61.1)	<0.001
Secondary	1,839 (22.7)	793 (22.3)	1,046 (23.0)	0.54
Higher Level	336 (4.1)	161 (4.5)	175 (3.8)	0.11
SES[2]				
Lower	2,309 (34.6)	1,048 (35.7)	1,261 (33.6)	0.08
Middle	2,175 (32.5)	945 (32.1)	1,230 (32.8)	0.59
Upper	2,203 (32.9)	944 (32.1)	1,259 (33.6)	0.22
HIV Status				
Negative	7,483 (92.5)	3,331 (94.1)	4,152 (91.2)	
Positive	608 (7.5)	208 (5.9)	400 (8.8)	<0.001
Hepatitis B				
Negative	7,878 (97.3)	3,420 (96.6)	4,458 (97.8)	
Positive	220 (2.7)	122 (3.4)	98 (2.2)	<0.001
Hepatitis C				
Negative	8,086 (99.8)	3,533 (99.7)	4,553 (99.9)	
Positive	13 (0.2)	9 (0.3)	4 (0.1)	0.06
BMI[3]				
Normal weight	5,095 (65.1)	2,259 (64.4)	2,836 (65.7)	0.23
Underweight	1,772 (22.7)	1,075 (30.6)	697 (16.1)	<0.001
Overweight/Obese	960 (12.2)	175 (5.0)	785 (18.2)	<0.001
**Alcohol Consumption[4]**				
Never drinkers	5,180 (64.0)	2,120 (59.9)	3,060 (67.2)	
Drinkers	2,919 (36.0)	1,422 (40.1)	1,497 (32.8)	<0.001

[1] p-value calculated to determine whether significant difference between males and females in each category using chi-square test

[2]Socio-economic Score (SES) derived from conducting Principle Component Analysis (PCA) on a statistical software using variables relating to household infrastructure and property ownership

[3]Body Mass Index (BMI) Classification according to WHO (weight/height^2: kg/m^2): Underweight (<18.5 kg/m^2), Normal weight (18.5 – 24.99 kg/m^2), Overweight (25.0 – 29.99 kg/m^2), Obese (>30.0 kg/m^2)

[4] Alcohol consumption based on self-reported history of consuming alcohol vs never consuming alcohol

For peer review only - http://bmjopen.bmj.com/site/about/guidelines.xhtml

Suppl Fig 1: Distribution of liver function tests in Uganda General Population Cohort.
Top row: ALT -alanine transferase, AST – aspartate transferase, GGT – gamma glutamyl transferase
Bottom row: BR – bilirubin, ALP – alkaline phosphatase.

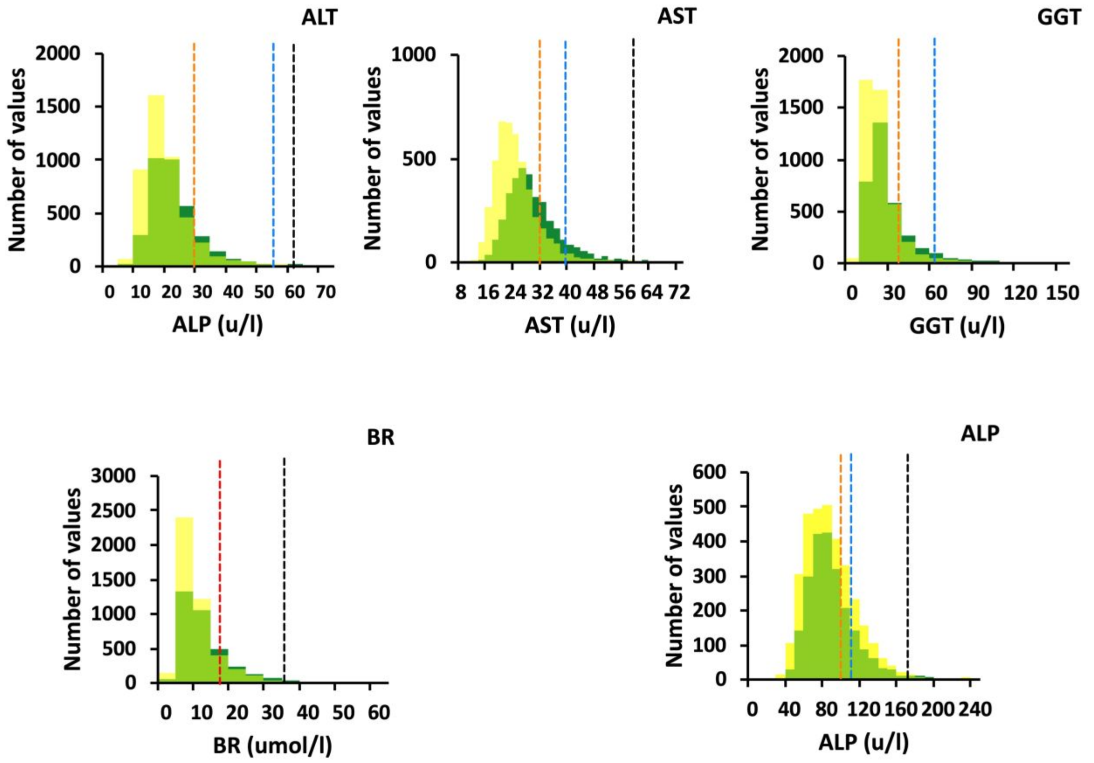

Suppl Fig 2: Odds ratio for deranged ALT, AST, APRI, GPR and AST/ALT among participants grouped by sex and age, by multivariate analysis.

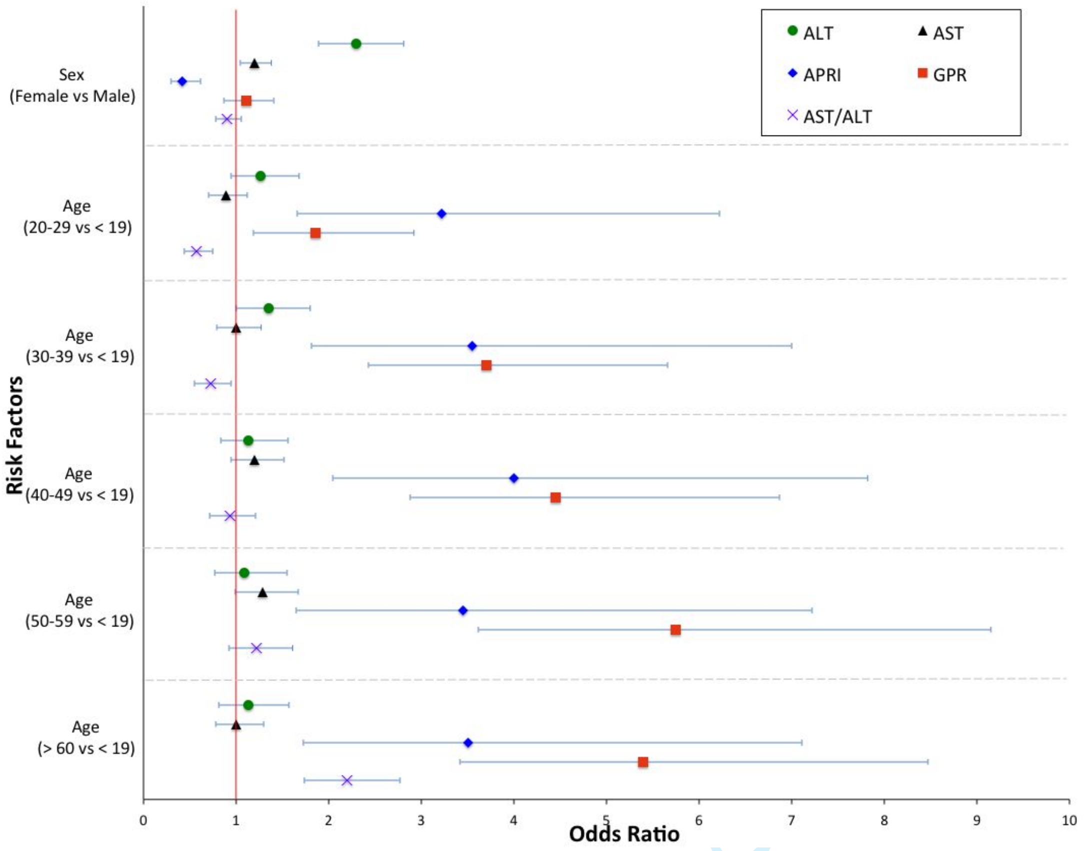

Suppl Fig 3: Proportion of Uganda General Population Cohort reporting alcohol consumption among individuals with and without AST/ALT ratio >2

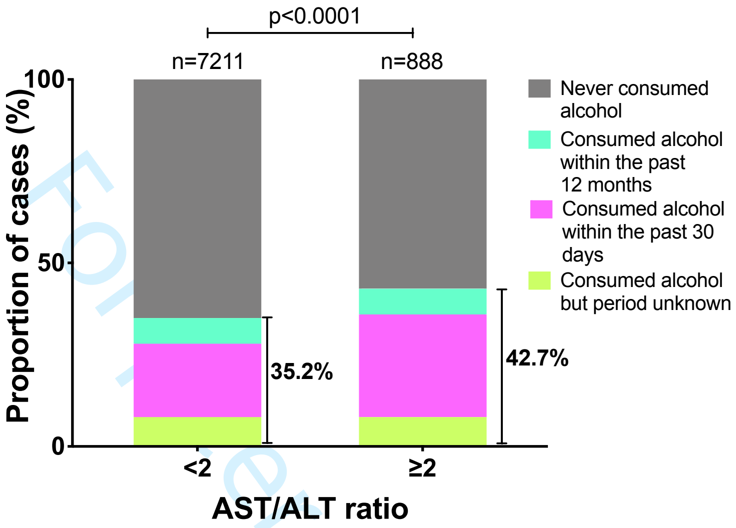

For peer review only

Suppl Fig 4: Proportion of Uganda General Population Cohort with elevated GGT, according to AST/ALT ratio. (A) males, with upper limit of normal GGT=61 (B) females, with upper limit of normal GGT=36. P-values by Fisher's Exact Test

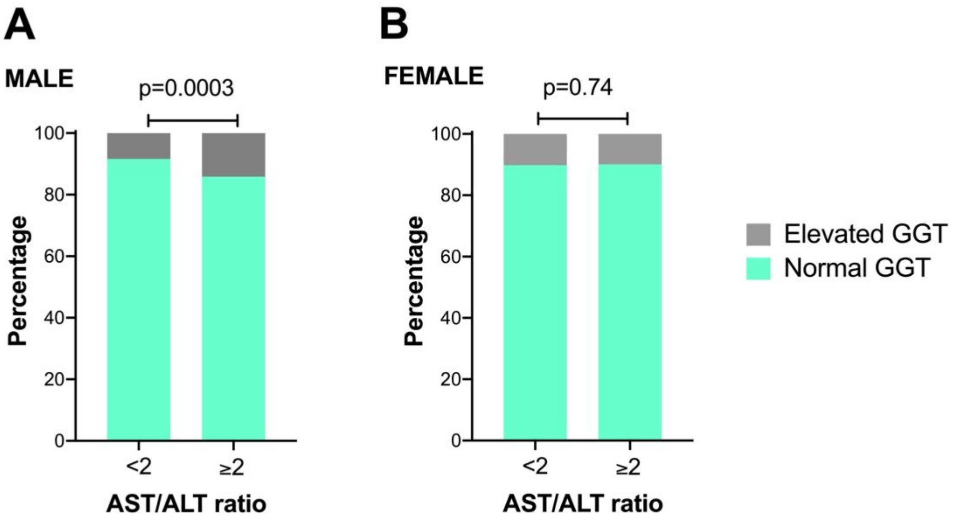

For peer review only - http://bmjopen.bmj.com/site/about/guidelines.xhtml

Suppl Fig 5: Proportion of Uganda General Population Cohort with blood borne virus (BBV) infection, according to GPR score. P-value by Fisher's Exact Test, showing significant enrichment of BBV infection among individuals with elevated GPR score >0.32.

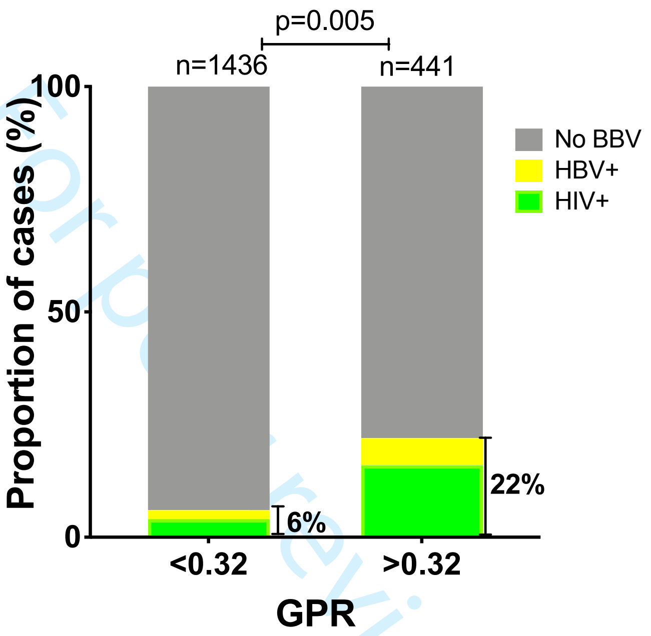

For peer review only

STROBE Statement—checklist of items that should be included in reports of observational studies

	Item No	Recommendation	Page No
**Title and abstract**	1	(*a*) Indicate the study's design with a commonly used term in the title or the abstract	1
		(*b*) Provide in the abstract an informative and balanced summary of what was done and what was found	3
**Introduction**			
Background/rationale	2	Explain the scientific background and rationale for the investigation being reported	4
Objectives	3	State specific objectives, including any prespecified hypotheses	6
**Methods**			
Study design	4	Present key elements of study design early in the paper	6
Setting	5	Describe the setting, locations, and relevant dates, including periods of recruitment, exposure, follow-up, and data collection	6
Participants	6	(*a*) *Cohort study*—Give the eligibility criteria, and the sources and methods of selection of participants. Describe methods of follow-up<br>*Case-control study*—Give the eligibility criteria, and the sources and methods of case ascertainment and control selection. Give the rationale for the choice of cases and controls<br>*Cross-sectional study*—Give the eligibility criteria, and the sources and methods of selection of participants	6
		(*b*) *Cohort study*—For matched studies, give matching criteria and number of exposed and unexposed<br>*Case-control study*—For matched studies, give matching criteria and the number of controls per case	n/a
Variables	7	Clearly define all outcomes, exposures, predictors, potential confounders, and effect modifiers. Give diagnostic criteria, if applicable	6,7
Data sources/ measurement	8*	For each variable of interest, give sources of data and details of methods of assessment (measurement). Describe comparability of assessment methods if there is more than one group	*6*
Bias	9	Describe any efforts to address potential sources of bias	11
Study size	10	Explain how the study size was arrived at	6
Quantitative variables	11	Explain how quantitative variables were handled in the analyses. If applicable, describe which groupings were chosen and why	7
Statistical methods	12	(*a*) Describe all statistical methods, including those used to control for confounding	7
		(*b*) Describe any methods used to examine subgroups and interactions	7
		(*c*) Explain how missing data were addressed	7,8
		(*d*) *Cohort study*—If applicable, explain how loss to follow-up was addressed<br>*Case-control study*—If applicable, explain how matching of cases and controls was addressed<br>*Cross-sectional study*—If applicable, describe analytical methods taking account of sampling strategy	6,7
		(*e*) Describe any sensitivity analyses	n/a

Continued on next page

For peer review only - http://bmjopen.bmj.com/site/about/guidelines.xhtml

**Results**			
Participants	13*	(a) Report numbers of individuals at each stage of study—eg numbers potentially eligible, examined for eligibility, confirmed eligible, included in the study, completing follow-up, and analysed	8,9 and tables
		(b) Give reasons for non-participation at each stage	n/a
		(c) Consider use of a flow diagram	n/a
Descriptive data	14*	(a) Give characteristics of study participants (eg demographic, clinical, social) and information on exposures and potential confounders	Suppl table 3
		(b) Indicate number of participants with missing data for each variable of interest	Tables
		(c) *Cohort study*—Summarise follow-up time (eg, average and total amount)	n/a
Outcome data	15*	*Cohort study*—Report numbers of outcome events or summary measures over time	*n/a*
		Case-control study—Report numbers in each exposure category, or summary measures of exposure	*n/a*
		Cross-sectional study—Report numbers of outcome events or summary measures	*Tables*
Main results	16	(*a*) Give unadjusted estimates and, if applicable, confounder-adjusted estimates and their precision (eg, 95% confidence interval). Make clear which confounders were adjusted for and why they were included	Tables
		(*b*) Report category boundaries when continuous variables were categorized	Tables
		(*c*) If relevant, consider translating estimates of relative risk into absolute risk for a meaningful time period	n/a
Other analyses	17	Report other analyses done—eg analyses of subgroups and interactions, and sensitivity analyses	n/a
**Discussion**			
Key results	18	Summarise key results with reference to study objectives	11
Limitations	19	Discuss limitations of the study, taking into account sources of potential bias or imprecision. Discuss both direction and magnitude of any potential bias	11,12
Interpretation	20	Give a cautious overall interpretation of results considering objectives, limitations, multiplicity of analyses, results from similar studies, and other relevant evidence	12,13
Generalisability	21	Discuss the generalisability (external validity) of the study results	12
**Other information**			
Funding	22	Give the source of funding and the role of the funders for the present study and, if applicable, for the original study on which the present article is based	14

*Give information separately for cases and controls in case-control studies and, if applicable, for exposed and unexposed groups in cohort and cross-sectional studies.

Note: An Explanation and Elaboration article discusses each checklist item and gives methodological background and published examples of transparent reporting. The STROBE checklist is best used in conjunction with this article (freely available on the Web sites of PLoS Medicine at http://www.plosmedicine.org/, Annals of Internal Medicine at http://www.annals.org/, and Epidemiology at http://www.epidem.com/). Information on the STROBE Initiative is available at www.strobe-statement.org.

For peer review only

Liver function tests and fibrosis scores in a rural population in Africa: a cross-sectional study to estimate the burden of disease and associated risk factors

Journal:	*BMJ Open*
Manuscript ID	bmjopen-2019-032890.R1
Article Type:	Research
Date Submitted by the Author:	16-Jan-2020

Complete List of Authors:	O'Hara, Geraldine; London School of Hygiene and Tropical Medicine, Faculty of Infectious and Tropical Diseases<br>Mokaya, Jolynne; Nuffied Department of Medicine<br>Hau, Jeffrey; London School of Hygiene and Tropical Medicine, Faculty of Infectious and Tropical Diseases<br>Downs, Louise; Oxford University Hospitals NHS Foundation Trust, Department of Microbiology and Infectious Diseases<br>McNaughton, Anna; University of Oxford, Nuffield Department of Medicine<br>Karabarinde, Alex; Medical Research Council/Uganda Virus Research Institute<br>Asiki, G; Medical Research Council/Uganda Virus Research Institute<br>Seeley, Janet; London School of Hygiene and Tropical Medicine, Department of Global Health &Development<br>Matthews, Philippa; University of Oxford, Nuffield Department of Medicine; Oxford University Hospitals NHS Foundation Trust, Department of Microbiology and Infectious Diseases<br>Newton, Robert; Medical Research Council/Uganda Virus Research Institute

**Primary Subject Heading**:	Gastroenterology and hepatology
Secondary Subject Heading:	Infectious diseases, Global health, HIV/AIDS, Public health
Keywords:	Epidemiology < TROPICAL MEDICINE, Hepatology < INTERNAL MEDICINE, HIV & AIDS < INFECTIOUS DISEASES

SCHOLARONE™
Manuscripts

I, the Submitting Author has the right to grant and does grant on behalf of all authors of the Work (as defined in the below author licence), an exclusive licence and/or a non-exclusive licence for contributions from authors who are: i) UK Crown employees; ii) where BMJ has agreed a CC-BY licence shall apply, and/or iii) in accordance with the terms applicable for US Federal Government officers or employees acting as part of their official duties; on a worldwide, perpetual, irrevocable, royalty-free basis to BMJ Publishing Group Ltd ("BMJ") its licensees and where the relevant Journal is co-owned by BMJ to the co-owners of the Journal, to publish the Work in this journal and any other BMJ products and to exploit all rights, as set out in our licence.

The Submitting Author accepts and understands that any supply made under these terms is made by BMJ to the Submitting Author unless you are acting as an employee on behalf of your employer or a postgraduate student of an affiliated institution which is paying any applicable article publishing charge ("APC") for Open Access articles. Where the Submitting Author wishes to make the Work available on an Open Access basis (and intends to pay the relevant APC), the terms of reuse of such Open Access shall be governed by a Creative Commons licence – details of these licences and which Creative Commons licence will apply to this Work are set out in our licence referred to above.

Other than as permitted in any relevant BMJ Author's Self Archiving Policies, I confirm this Work has not been accepted for publication elsewhere, is not being considered for publication elsewhere and does not duplicate material already published. I confirm all authors consent to publication of this Work and authorise the granting of this licence.

For peer review only

Liver function tests and fibrosis scores in a rural population in Africa: a cross-sectional study to estimate the burden of disease and associated risk factors

Geraldine A O'Hara[1,3*], Jolynne Mokaya[2*], Jeffrey P Hau[1,3], Louise O Downs[2.4], Anna L McNaughton[2], Alex Karabarinde[3], Gershim Asiki[3,5], Janet Seeley[3,6], Philippa C Matthews[2,4#] Robert Newton[3,7#]

[1]Faculty of Infectious and Tropical Diseases, London School of Hygiene and Tropical Medicine, London, United Kingdom

[2]Nuffield Department of Medicine, University of Oxford, Medawar Building for Pathogen Research, South Parks Road, Oxford OX1 3SY, UK;

[3] Medical Research Council/Uganda Virus Research Institute and London School of Hygiene and Tropical Medicine Uganda Research Unit, Entebbe, Uganda;

[4]Department of Infectious Diseases and Microbiology, Oxford University Hospitals NHS Foundation Trust, John Radcliffe Hospital, Headington, Oxford OX3 9DU, UK;

[5]African Population and Health Research Center, Nairobi, Kenya;

[6]Department of Global Health and Development, London School of Hygiene and Tropical Medicine, Tavistock Place, London, UK;

[7]Department of Health Sciences, University of York, York, United Kingdom;

* These authors contributed equally

\# These authors contributed equally

CORRESPONDING AUTHOR: Philippa Matthews, Email: philippa.matthews@ndm.ox.ac.uk

KEY WORDS

Hepatitis, liver function tests, epidemiology, prevalence, alcohol, alcoholic liver disease, HBV, HIV, Uganda, Africa, cirrhosis, fibrosis

ACKNOWLEDGEMENTS: Nil

For peer review only

ABBREVIATIONS

- ALT – alanine transferase
- APRI - AST to Platelet Ratio Index
- ARR – American reference range
- AST – aspartate transaminase
- BR – bilirubin
- BBV – blood borne virus (HIV, HBV, HCV)
- FIB-4- fibrosis-4
- GGT – gamma glutamyl-transferase
- GPC – general population cohort, Uganda
- GPR - GGT to platelet ratio
- HBV – hepatitis B virus
- HCV – hepatitis C virus
- HIV – human immunodeficiency virus
- LFTs – liver function tests
- LRR – local reference range
- NAFLD – non-alcoholic fatty liver disease
- OR – odds ratio
- PAR – population attributable risk
- RPR- red cell distribution width to platelet ratio
- sSA – sub Saharan Africa
- ULN – upper limit of normal
- USS – ultrasound scan
- WHO – World Health Organisation

For peer review only

ABSTRACT

Objectives: Liver disease is a major cause of morbidity and mortality in sub-Saharan Africa, but its prevalence, distribution and aetiology have not been well characterised. We therefore set out to examine liver function tests (LFTs) and liver fibrosis scores in a rural African population.

Design: We undertook a cross-sectional survey of LFTs. We classified abnormal LFTs based on reference ranges set in America and in Africa. We derived fibrosis scores (AST to Platelet Ratio Index (APRI), fibrosis-4 (FIB-4), GGT to platelet ratio (GPR), red cell distribution width to platelet ratio (RPR), and S-index). We collected information about alcohol intake, and infection with human immunodeficiency virus (HIV), hepatitis B virus (HBV) and hepatitis C virus (HCV).

Setting: We studied a population cohort in South-Western Uganda.

Participants: Data were available for 8,099 adults (median age 30 years; 56% female).

Results: The prevalence of HBV, HCV and HIV infection was 3%, 0.2% and 8%, respectively. The prevalence of abnormal LFTs was higher based on the American reference range compared to the African reference range (e.g. for AST 13% vs 3%, respectively). Elevated AST/ALT ratio was significantly associated with self-reported alcohol consumption ($p<0.001$), and the overall prevalence of AST/ALT ratio >2 was 11% (suggesting alcoholic hepatitis). The highest prevalence of fibrosis was predicted by the GPR score, with 24% of the population falling above the threshold for fibrosis. There was an association between the presence of HIV or HBV and raised GPR ($p=0.005$) and S-Index ($p<0.001$). By multivariate analysis, elevated LFTs and fibrosis scores were most consistently associated with older age, male sex, being under-weight, HIV or HBV infection, and alcohol consumption.

Conclusions: Further work is required to determine normal reference ranges for LFTs in this setting, to evaluate the specificity and sensitivity of fibrosis scores, and to determine aetiology of liver disease.

For peer review only - http://bmjopen.bmj.com/site/about/guidelines.xhtml

ARTICLE SUMMARY

Strengths and Limitations of the Study

- This is a cross sectional study of a large well-defined population cohort in rural South-Western Uganda where the burden of liver disease and its aetiology is not well described, based on liver function tests (LFTs).
- Our cross-sectional analysis of LFTs and fibrosis scores provides insights into some of the risk factors for liver disease, allowing us to make preliminary estimates of the burden of liver disease, and particularly highlighting a significant contribution of alcohol. .
- LFTs are a blunt tool for assessment of liver health, with many potential confounding factors. This current study only accounts for a limited range of aetiological agents.
- LFTs were measured at only one point in time, potentially overcalling liver disease as a result of transient abnormalities.
- A high HIV prevalence may be a confounding factor, causing abnormalities in platelet counts and elevation in LFTs that may not correlate well with underlying liver disease.

For peer review only

For peer review only - http://bmjopen.bmj.com/site/about/guidelines.xhtml

INTRODUCTION

Liver disease causes an estimated 200,000 deaths each year in sub-Saharan Africa (sSA) as a result of liver cirrhosis and hepatocellular carcinoma (1). More than 80% of Africa's burden of liver disease has been attributed to endemic blood borne virus (BBV) infections, such as HIV, hepatitis B (HBV) and hepatitis C (HCV), alcohol, hepatotoxic medications (including traditional and herbal medicines), non-alcoholic fatty liver disease (NAFLD) and exposure to aflatoxins (1–3). However, the prevalence, distribution and aetiology of liver disease in many parts of Africa have not been well characterised, and the neglect of cirrhosis has recently been highlighted (2). In order to improve screening for liver disease, and to implement appropriate investigations and intervention, we have undertaken a survey of liver function tests (LFTs) together with demographic data for a large rural cohort in South-Western Uganda (4).

The term 'LFTs' can be ambiguous, as it is widely applied to biochemical markers of liver inflammation or biliary obstruction, rather than genuine hepatic function. These include aspartate aminotransferase (AST), alanine aminotransferase (ALT), alkaline phosphatase (ALP), gamma-glutamyl transferase (GGT), and bilirubin (BR). This panel of blood biomarkers is usually the first approach to evaluation of liver disease; reference ranges and causes of derangement are summarised in Suppl Table 1 (5). In addition, true tests of liver synthetic function can be assessed by measuring prothrombin time or albumin, and platelet production may be decreased in chronic liver disease due to hypersplenism, decreased thrombopoietin levels and bone marrow suppression (6). Abnormal LFTs are often non-specific and can arise transiently in association with many acute illnesses or usage of medications. However, persistent derangement can indicate chronic liver disease, with associated morbidity and mortality (7). The pattern of derangement can sometimes help to establish aetiology – for example AST/ALT ratio >2 is characteristically associated with alcoholic hepatitis (8,9).

Determination of the origin of liver disease and stratification for treatment necessitates estimation of the extent and nature of hepatic injury. Liver biopsy allows objective grading of fibrosis and can provide information about the likely aetiology of liver disease based on specific changes to cellular architecture. However, biopsy is costly, requires experts to undertake the procedure and analyse samples, and exposes patients to potentially life-threatening risks. Imaging can also be employed to assess fibrosis. Typically, this comprises ultrasound-based techniques, including fibroscan to derive elastography scores. In most low and middle-income settings, evaluation of liver disease currently depends on use of non-invasive (blood) markers, often combined with ultrasound and/or fibroscan when available.

For peer review only - http://bmjopen.bmj.com/site/about/guidelines.xhtml

Non-invasive fibrosis blood tests are relatively simple and offer a safe route to assess for liver fibrosis, appealing in resource limited settings. Scores of liver fibrosis, such as AST to Platelet Ratio Index (APRI), fibrosis-4 (FIB-4), GGT to platelet ratio (GPR), red cell distribution width to platelet ratio (RPR) and S-index have been derived using liver enzymes (ALT, AST, GGT) in combination with platelet count. However, diagnostic accuracy is not well established in sSA and can be influenced by the population being assessed and the nature of underlying liver disease (10–15). GPR has recently been reported as an independent predictor of significant fibrosis in naïve Gambian patients with chronic hepatitis B (CHB) infection (13), while the usefulness of cut-off values for APRI scores in CHB has been questioned (16). However, further studies are needed to determine the specificity and sensitivity of different scores in different settings.

Appropriate reference ranges for LFTs are crucial for optimising the detection of underlying liver disease (17). Application of reference ranges defined in one setting to different populations may lead to either under- or over-estimation of abnormalities (17–19). As well as being dependent on the population being assessed, the distribution of LFTs in any given setting can also be influenced by the type of instrument, reagents used, and the strength of quality assurance (19). Efforts have been made to establish 'population-specific' reference ranges (18,20); one example is through the application of cross-sectional data from seven South-Eastern African countries (18). However, such local reference ranges for Africa have been derived from cross-sectional data collected in adults without addressing the potential prevalence of underlying liver disease. Thus, while American reference ranges potentially over-estimate of the burden of liver disease in an African setting, it is also possible that locally derived reference ranges under-estimate the burden (as they are based on thresholds that have been derived from populations in which liver disease is highly prevalent).

We here set out to assess LFTs and fibrosis scores derived from a large, well defined population cohort in rural South-Western Uganda (21). We applied reference ranges set in both America and in Africa (18,22), in order to assess the possible burden of liver disease, highlighting the discrepancies that arise as a result of the difference between thresholds. We derived fibrosis scores to further evaluate the potential prevalence of liver disease in this setting and to estimate the contributions of alcohol and BBVs to the burden of disease.

METHODS

For peer review only - http://bmjopen.bmj.com/site/about/guidelines.xhtml

Study design and study population

We conducted a cross-sectional study in a rural population in Kyamulibwa, in the Kalungu district of South-Western Uganda as part of the survey of the General Population Cohort (GPC). The GPC is a community-based cohort established in 1989 with funding from the UK Medical Research Council (MRC) in collaboration with the Uganda Virus Research Institute (UVRI) (23). Regular census and medical surveys have been conducted in this population cohort. In 2011, data collection included screening for viral hepatitis and LFTs among 8,145 adults (≥16 years), which we used for this analysis.

Data collection

Demographic and health history data were collected using questionnaires and standardised procedures described elsewhere (23,24). Blood samples were drawn at home and transported for testing at the Medical Research Council central laboratories in Entebbe. LFTs (serum AST, ALT, ALP, GGT and BR) were measured using a Cobas Integra 400 plus machine, with Roche reagents. Screening for HIV testing was done using an algorithm recommended by the Uganda Ministry of Health, based on initial screening with a rapid test. If the test result was negative, the participant was considered to be HIV negative. If the test result was positive, the sample was re-tested with the rapid test HIV-1 or -2 Stat-Pak. If both tests resulted in a positive result, the participant was diagnosed as HIV positive. If the tests gave discordant results, the sample was further evaluated with the rapid test Uni-Gold Recombinant HIV-1/2. For those samples assessed by all three tests, two positive test results were interpreted as positive, and two negative results were considered negative. HBV surface antigen (HBsAg) testing was conducted using Cobas HBsAg II (2011-08 V10), and those who tested positive were invited for further serologic testing. HCV was tested using a combination of immunoassays followed by PCR, as previously described (25). Normal serum levels of liver enzymes were classified according to the American reference range (ARR, MGH Clinical Laboratories) and Local Reference Ranges (LRR, (18); values listed in Suppl Table 1 (5)). We excluded individuals ≤19 years from ALP analysis, since elevated ALP can be attributable to bone growth in teenagers.

Data from the full blood count was used to calculate fibrosis scores (mean corpuscular volume, MCV and platelet count). This was collected starting part-way through the 2011 data collection period; the data are, therefore, population-based, although based on only a subset of the whole cohort (n=1,877).

Calculation of fibrosis scores and AST/ALT ratio

Where data were available (n=1,877), we calculated APRI, FIB-4, GPR, RPR and S-Index. The formulae for calculating these scores are presented in Suppl Table 2 (5), along with the sensitivity and

For peer review only - http://bmjopen.bmj.com/site/about/guidelines.xhtml

specificity of each, based on previous studies. We used previously established thresholds to suggest the presence of liver fibrosis, as follows: APRI >0.7 (26), FIB-4 >3.25 (27), GPR >0.32 (13), RPR >0.825 (28), S-index >0.3 (29). We calculated AST/ALT ratio; a score >2 has been associated with alcoholic hepatitis (9).

Statistical Analysis

We analysed data using standard statistical software, Stata/IC 13 (Stata Corporation, College Station, USA) and GraphPad Prism v7.0. We summarised participant baseline characteristics using proportions (%) and these were stratified by sex. We reported prevalence and distribution of LFTs, laboratory markers of fibrosis and elastography scores using descriptive statistics. We reported p-values from chi-square tests, comparing the proportions of each potential risk factor between male and female participants. We also reported the medians and inter-quartile ranges (IQR) of each LFT and liver fibrosis scores. We compared the difference in medians of LFTs and liver fibrosis scores for each potential risk factor using the Kruskal-Wallis test.

We used logistic regression in our univariate and multivariate analyses, using the threshold for significance set at 0.05, to estimate the odd ratios (OR), along with its 95% confidence intervals (95% CI), to identify potential factors associated with abnormal LFTs and liver fibrosis scores, using a forward stepwise approach to develop our multivariate models. We added risk factors that were identified in the age and sex adjusted analysis to the multivariate model. The final multivariate models for each LFT and liver fibrosis score were obtained by excluding variables in the final model until all remaining variables were associated with abnormal LFTs and liver fibrosis scores at the p<0.05 threshold. Once the final multivariate model had been established, variables that were eliminated through this forward stepwise approach were added back to the model and were reported if associated at the p<0.05 threshold, to reduce the effects of residual confounding.

Due to the low number of individuals with active HCV infection at the time of the study, we did not include this sub-group in univariate or multivariate analysis. These HCV RNA positive individuals have been described in more detail elsewhere (30). We present results of multivariate analysis in the form of Forrest plots generated using Microsoft Excel.

We calculated population attributable risk (PAR) as the proportion of the cases of liver dysfunction (defined either as elevated LFTs or fibrosis score) in the population that is due to exposure to alcohol,

For peer review only

HIV or HBV. This provides us with an estimate of the proportion of liver dysfunction that would be eliminated if exposure were removed (31).

Ethics

Ethics approval was provided by the Science and Ethics Committee of the Uganda Virus Research Institute (GC/127/12/11/06), the Ugandan National Council for Science and Technology (HS870), and the East of England-Cambridge South (formerly Cambridgeshire 4) NHS Research Ethics Committee UK (11/H0305/5). All participants provided written informed consent.

Patient and public involvement

Patients and the public were not involved in the design, conduct or reporting of this research.

RESULTS

Characteristics of study population

We analysed complete data for 8,099 participants (summarised in Suppl Table 3 (5)). Compared to females, there were more males who were HBV positive, (prevalence 3% vs 2%, respectively; $p<0.001$) and had consumed alcohol in the past 30 days, (40% vs 33%, respectively; $p<0.001$). More females were HIV positive (9% vs 6%, respectively; $p<0.001$). Males were more likely to be underweight (31% vs 16%), and females to be overweight (18% vs 5%); $p<0.001$ in both cases. Median and IQR for each parameter analysed are presented in Suppl Table 4 (5).

Proportion of population defined as having abnormal LFTs varies according to the reference range that is applied

The proportion of the population falling above the upper limit of normal (ULN) for each parameter is shown in Table 1, with ALT, AST and GGT distributions in Fig 1A-C (full data for all LFTs are shown in Suppl Fig 1 (5)). These results highlight the different burden of disease that can be estimated according to the reference range that is applied, with a higher proportion of the population falling above the ULN when the ARR was applied compared to the LRR (Fig 1A, B). Most striking, for AST, 13% of the population had a value that was deemed to be elevated based on ARR, compared to only 3% based on the LRR (Fig 1B). Using the ARR, ALT and BR were significantly more likely to be above the ULN in males than in females, and ALP was more likely to be higher in females ($p<0.001$ in each case, Table 1). These sex differences were not apparent when the LRR was applied. OR for deranged LFTs and fibrosis scores according to age and sex is shown in Suppl. fig 2 (5).

For peer review only - http://bmjopen.bmj.com/site/about/guidelines.xhtml

For peer review only

The highest prevalence of liver fibrosis is predicted using the GPR score

We calculated APRI, FIB-4, GPR, RPR and S-index scores (Table 1). The estimated prevalence of fibrosis was highest when based on GPR score (23.5%; Fig 1D), compared to FIB-4 (5.3%), APRI (3.2%), S-index (3.9%) and RPR (0.1%). We excluded RPR scores from further statistical analysis because so few individuals were classified as having an elevated score (we therefore did not have statistical power to detect any factors associated with abnormal score). Because the APRI is derived using the ULN of AST, the proportion of the population classified as having a score consistent with liver fibrosis changes according to whether the ARR or LRR is used (Table 1). Based on previous validation among African individuals, there is some limited evidence to suggest that GPR is the most accurate score for staging liver fibrosis (13); applying this approach, there is a prevalence of almost 1 in 4 adults with liver fibrosis in this population.

Evidence for the contribution of alcohol to liver disease

The prevalence of AST/ALT ratio >2, suggestive of alcoholic hepatitis, was 11% (888/8,099) (Fig 1E). The median and IQR of GGT among alcohol drinkers were significantly larger than non-drinkers (23.2 (15.6-38.9) vs 17.3 (12.8-23.7)); (Suppl Table 4 (5)). There was a significant relationship between self-reported alcohol consumption and elevated AST/ALT ratio ($p<0.001$; Suppl Fig 3 (5)). However, 57% of participants with AST/ALT ratio >2 reported never having consumed alcohol (Fig 1E), possibly reflecting either under-reporting of alcohol use and/or other factors that underpin this pattern of LFTs. Self-reported alcohol consumption was associated with raised LFTs, as follows: ALT (Adj. OR 1.33, 95% CI 1.09,1.63) AST (Adj. OR 1.53, 95% CI 1.30, 1.78) GGT (Adj. OR 2.00 95% CI 1.69, 2.36), and with abnormal fibrosis scores, particularly GPR (Adj. OR 1.96, 95% CI 1.52, 2.54). All ORs, adjusted ORs, their respective 95% confidence intervals and p-values are shown in Table 2, and selected variables in Fig 2.

A raised GGT level in combination with AST/ALT ratio >2 can be used to increase the sensitivity of detection of alcoholic hepatitis (9). GGT levels were significantly higher among males with AST/ALT ratio ≥2 ($p<0.001$), but there was no relationship between GGT and AST/ALT ratio in females ($p=0.7$); Suppl Fig 4 (5). This potentially indicates that alcohol is of more influence as a cause of an elevated AST/ALT ratio in men than in women. There was no significant association between AST/ALT ratio ≥2 and the presence of an elevated GPR score, predicting fibrosis ($p=0.2$; data not shown). We calculated population attributable risk (PAR) as a way to assess the relative contribution of different risk factors to the overall burden of liver disease; Table 3. Overall, the most striking contribution arose from reported

For peer review only - http://bmjopen.bmj.com/site/about/guidelines.xhtml

alcohol consumption, which accounted for 64% of abnormal S-index scores, 32% of elevated FIB-4 scores, and 19% of GPR abnormalities.

Abnormal LFTs and/or elevated fibrosis scores are associated with sex, age, and body mass index

Compared to males, females were less likely to have high fibrosis scores based on FIB-4 (Adj. OR: 0.6), APRI (Adj. OR: 0.42), and S-Index (Adj. OR: 0.37). FIB-4 score increased markedly with age: adults aged 40 – 49 (Adj. OR: 7.04), 50 – 59 (Adj. OR: 11.29), and adults >60 years (Adj. OR: 25.15) were more likely to have a higher FIB-4 than individuals <40 years. Elevated BMI was associated only with a rise in GGT (Adj. OR: 1.47). However, being underweight was associated with a more pronounced pattern of liver derangement, including elevations in ALT (Adj. OR: 1.40), AST (Adj. OR: 1.44), GGT (Adj. OR: 1.37), abnormal fibrosis scores (APRI Adj. OR: 1.72,) and with raised AST/ALT ratio (Adj. OR: 1.61). 95% CI in each case are shown in Table 2.

Relationship between BBV infection and liver disease

HIV infection was associated with abnormal liver function tests, with significant OR for increased ALT, AST, ALP and GGT, as well as with raised GPR and S-index (on univariate and multivariate analysis; Table 2). Individuals with HIV or HBV infection had higher liver function tests (ALT, AST, ALP, GGT) and elevated liver fibrosis scores (FIB-4, APRI, GPR, and S-Index) compared to uninfected individuals (Suppl Table 4 (5)). HBV infection was significantly associated with a rise in hepatic transaminases (Adj. OR for raised ALT and AST 2.6 and 2.4 respectively), and with liver fibrosis as measured by APRI and GPR (Adj. OR 3.6 and 4.2 respectively). We investigated the prevalence of BBV infection among individuals with raised fibrosis scores. There was an association between the presence of HIV or HBV and raised GPR (p=0.005) and S-Index (p<0.001). HIV and HBV were associated with a lesser proportion of liver disease than alcohol based on calculation of PAR (Table 3), but still contributed to elevations in both LFTs and fibrosis scores. The OR for deranged LFTs/fibrosis scores in the context of HIV or HBV infection is shown in Fig 2.

Liver disease of unknown aetiology

Among individuals with GPR>0.32, 33.8% had either BBV infection or had AST/ALT ratio >2 (suggesting potential alcoholic hepatitis) (Fig 1D; Suppl Fig 5 (5)). However, this illustrates that 66% have raised fibrosis scores in the absence of a history of alcohol use, or HIV or HBV infection, suggesting that other factors unaccounted for in this study are likely to be contributing to the overall burden of liver disease. In the setting of a population-based cohort (where the background prevalence

For peer review only - http://bmjopen.bmj.com/site/about/guidelines.xhtml

of liver fibrosis is relatively low), many of those with an abnormal test result may not have liver disease; these 'false positive' cases of elevated GPR may also account for some of the 66% in whom we could not identify a risk factor. True prevalence of liver disease cannot be ascertained until reference ranges have been more carefully defined, correlating LFTs and fibrosis scores with the confirmed presence of underlying liver disease based on imaging or biopsy.

DISCUSSION

Liver disease is not well characterised in many parts of sSA despite the high prevalence of HIV and HBV, and potential exposure to hepatotoxins (1,3). In this study, we used cross-sectional data from a large population cohort to estimate the burden of liver disease and to assess the possible impact of BBV infection and alcohol consumption. The prevalence of abnormal LFTs depends on the reference range that is applied. The ARR suggests a higher prevalence of liver disease, therefore including more false-positives. The LRR was established based on individuals recruited from several countries across Africa (Rwanda, Uganda, Kenya, Zambia) (18). While the values were derived from purportedly healthy adults, it is impossible to rule out a high background prevalence of underlying liver disease; in defining higher values for the ULN of all tests, the LRR is more susceptible to false-negatives if used to screen for liver disease. Composite fibrosis scores have been developed with the aim of improving sensitivity of detection of liver disease (32), but these it is striking that there is a large variation in the prevalence of liver fibrosis estimated by different scores, ranging from 23.5% based on assessment using GPR, down to <1% with RPR. This discrepancy highlights the differing performance of different scores, but in the absence of elastography data we are currently unable to determine which test offers the most accurate assessment.

LFTs are a blunt tool for assessment of liver health, with many potential confounding factors. This current study only accounts for a limited range of aetiological agents, and we did not include other potentially relevant factors such as Schistosomiasis infection, exposure to aflatoxin and use of traditional medications. Furthermore, LFTs were measured at only one point in time, potentially overcalling liver disease as a result of transient abnormalities. Further studies will be required to investigate a greater range of risk factors, and to undertake longitudinal follow-up.

Fibrosis scores also depend on platelet count which can be influenced by diverse factors. For example, in some African populations, thrombocytopenia is common due to infections such as malaria, schistosomiasis, HIV or endemic parasites, as well as being influenced by inflammatory conditions and

For peer review only - http://bmjopen.bmj.com/site/about/guidelines.xhtml

certain drugs (10,11). We only had platelet counts for a sub-set of our study population, limiting the number for whom we could determine APRI, FIB-4, GPR, S-Index and RPR scores. Data surrounding the use of these scores in sSA is variable, but since in many low-income settings alternative diagnostic equipment is unavailable, non-invasive approaches are vital to estimate liver damage and to stratify clinical management decisions. The finding that almost 1:4 individuals in this population study had an abnormal GPR score is concerning and striking. This could be influenced by high GGT values (potentially in association with alcohol), or low platelet counts (for the reasons outlined above). However, it should also be noted that we used stringent thresholds for GGT, with different thresholds for the upper limit of normal in males and females (Suppl Table 1 (5)), which influence the proportion of the population meeting the threshold for elevation of both GGT and GPR.

APRI and FIB-4 are currently recommended by the World Health Organisation (WHO) for assessment of hepatic fibrosis in patients with chronic HBV or HCV infection (33,34). However, the evidence is limited, and to some extent conflicting. One report concludes that APRI is more accurate in assessing liver fibrosis among individuals with chronic HCV compared to HBV infection (12). Meanwhile, GPR and S-Index have been validated in small studies in sSA, and have been associated with improved classification of liver fibrosis in chronic HBV infection when compared to APRI and FIB-4 (13–15). A study in Ethiopia reported a similar specificity of APRI, GPR and FIB-4 for the detection of fibrosis and cirrhosis [ref Desalegn doi: 10.1111/liv.13393]. It is apparent that either larger studies, or indeed a meta-analysis, are required to further assess the accuracy of these tests in different populations and in the context of different underlying disease processes. GPR and S-index may be worthwhile options to include in routine clinical practice to assess for liver fibrosis in African populations, given the high burden of HBV in this continent (35,36). RPR has been used to detect fibrosis among individuals with chronic HBV in China (28), however this score was excluded from our analysis due to a very small number of individuals falling above the suggested threshold for fibrosis.

The prevalence of AST/ALT ratio >2 in this population is 11%, suggesting potential alcoholic hepatitis (37), concordant with a previous study in Uganda in which 10% of the population was estimated to have alcoholic hepatitis (38), and with data from Uganda's non-communicable diseases risk factor survey which estimated that almost 10% of Ugandan adults have alcohol use disorders (39). Data from emergency attendances at Mulago Hospital in Kampala recorded 47% who reported alcohol use, while 21% and 10% met the study definitions of alcoholic misuse and alcoholic liver disease, respectively (38). Our data are based on self-reported alcohol consumption so may underestimate the true extent of alcohol use. We were unable to quantify alcohol intake or the nature of the alcohol

For peer review only - http://bmjopen.bmj.com/site/about/guidelines.xhtml

consumed: this is challenging as alcohol is often home-brewed or home-distilled from locally grown grains or fruits, and the alcohol content may vary widely; e.g. the alcohol content of locally produced maize-based brews and liquor in Kenya ranged from 2%-7% and 18%-53%, respectively (39). The global challenge of morbidity and mortality associated with alcohol use is highlighted by recent studies from the Global Burden of Disease consortium, in which alcohol ranks as the seventh highest cause of DALYs and deaths and worldwide (2), and together with HBV infection is a leading aetiological agent of liver cancer (40). Further data collection using validated tools to quantify the frequency, volume and patterns of alcohol consumption will be important to improve insights into the relationship between alcohol and liver disease in our population setting.

The calculation of PAR that we have undertaken in this study should be interpreted with caution, as we recognise that robust assessment of exposure to alcohol is difficult, and the markers we are using to represent underlying liver disease each comes with associated caveats. We have nevertheless included this analysis as part of our output on the grounds that it is congruent with other aspects of the analysis in highlighting a likely significant role for alcohol as a driver of liver disease, and therefore may be of influence in informing future studies as well as underpinning appropriate interventions.

Abnormal LFTs are common in HIV infection for diverse reasons including direct cytopathic effects of HIV on hepatocytes, co-infection with other BBVs, opportunistic infection, malignancy, ART or other drugs, or secondary to other factors such as alcoholism (41–44). Although a proportion of our study population with fibrosis were infected with BBV (21.6%) and/or had a history of alcohol consumption (12.2%), there was a residual proportion with scores suggestive of fibrosis and AST/ALT ratio >2 who cannot be accounted for through either alcohol or BBV infection. This is in keeping with other studies from Africa that report a high proportion of cases of liver disease that are not attributable to viral infection or alcohol and could be as a result of other understudied factors such as NAFLD and use of traditional medicine (38,45). Khat chewing (a popular recreational drug in some settings), was recently found to be a major cause of unexplained liver disease in east Ethiopia (45). Aflatoxin exposure is associated with liver cirrhosis and is among the major causes of hepatocellular carcinoma globally, with most cases reported from sSA. Within a previous study of the GPC, >90% of individuals had evidence of exposure (46–48).

In our population women were significantly more likely to be overweight women than men. This may be associated with a higher incidence of NAFLD in women. However, typically only mild rises in ALT are

For peer review only - http://bmjopen.bmj.com/site/about/guidelines.xhtml

seen, and 80% of those with NAFLD have normal LFTs (49–51) so may not be identified within our current dataset. Diagnosis of NAFLD therefore depends on ultrasound scan (USS); previous studies have consistently shown 70-80% of obese patients have NAFLD on imaging (50,52,53). These imaging modalities were not available in our population, so we are unable to comment specifically on the possible prevalence of NAFLD. Interestingly, in this setting low body weight was more associated with deranged LFTs and with biochemical evidence of liver fibrosis, suggesting a range of pathology that may contribute to liver disease, including organ-specific effects of under-nutrition or stunting (40), as well as the effect of general systemic illness. Further studies are required to investigate the specific relationship between BMI and liver fibrosis in African populations.

In African populations, HCV infection has frequently been often over-reported due to a reliance on HCV-antibody (HCV-Ab) testing, which detects not only current infection but also previous exposure, and is known to be susceptible to false positive results (30). In this cohort, 298/8145 (3.7%) individuals tested HCV-Ab positive, but among these only 13 were HCV RNA positive (overall prevalence 13/8145 = 0.2%).

Appropriate reference ranges for LFTs are necessary to contribute to an understanding of the burden and aetiology of liver disease. Further work is required to determine appropriate thresholds for the ULN of different parameters in different settings in sSA, and to determine which fibrosis score is most specific, through application of a more widespread approach to elastography and/or other imaging. At present, we have identified alcohol, HIV and HBV as risk factors for deranged LFTs and elevated liver fibrosis scores, with a striking contribution made by alcohol, but further investigation is needed to determine other risk factors that contribute to liver disease in this setting.

For peer review only

DECLARATIONS:

CONSENT TO PUBLISH

All authors approve the publication of this manuscript

DATA SHARING STATEMENT

All data generated or analysed during this study are included in this published article, and its Supplementary Information files which are accessible on-line at Figshare: https://doi.org/10.6084/m9.figshare.8292194 (5).

CONFLICT OF INTEREST

We have no conflicts of interest to declare.

FINANCIAL SUPPORT

The General Population Cohort is jointly funded by the UK Medical Research Council (MRC) and the UK Department for International Development (DFID) under the MRC/DFID Concordat agreement. The work on liver function also received additional funding from the MRC, (grant numbers G0801566 and G0901213-92157). JM is funded by a Leverhulme Mandela Rhodes Scholarship. PCM is funded by the Wellcome Trust, grant number 110110. LOD is funded by NIHR.

AUTHORS' CONTRIBUTIONS:

- Conceived the study : GAO'H, JS, PCM, RN
- Data collection : AK, GA, JS, RN
- Analysed the data : JM, JPH, LOD, ALM, PCM
- Wrote the manuscript : GAO'H, JM, JPH, LOD, PCM, RN
- Revised the manuscript : All authors

All authors have read and approved the manuscript

ACKNOWLEDGEMENTS

Nil

For peer review only

REFERENCES

1. Spearman CW, Sonderup MW. Health disparities in liver disease in sub-Saharan Africa. Vol. 35,Liver International. 2015. p. 2063–71.
2. Vento S, Dzudzor B, Cainelli F, Tachi K. Liver cirrhosis in sub-Saharan Africa: neglected, yet important. Lancet Glob Heal. 2018;6(10):e1060–1.
3. Price JC, Thio CL. Liver Disease in the HIV-Infected Individual. Clin Gastroenterol Hepatol. 2011;8(12):1002–12.
4. Hotez PJ, Kamath A. Neglected tropical diseases in sub-saharan Africa: review of their prevalence, distribution, and disease burden. PLoS Negl Trop Dis. 2009 A;3(8):e412.
5. O'Hara G, Mokaya J, Hau J, Downs L, Karabarinde A, Asiki G et al. (2019): Liver function tests and fibrosis scores in a rural population in Africa. figshare. Dataset. https://doi.org/10.6084/m9.figshare.8292194
6. Mitchell O, Feldman DM, Diakow M, Sigal SH. The pathophysiology of thrombocytopenia in chronic liver disease. Hepat Med. 2016;8:39–50.
7. Radcke S, Dillon JF, Murray AL. A systematic review of the prevalence of mildly abnormal liver function tests and associated health outcomes. Eur J Gastroenterol Hepatol. 2015;27(1):1–7.
8. Botros M, Sikaris KA. The de ritis ratio: the test of time. Clin Biochem Rev. 2013 34(3):117–30.
9. Limdi JK. Evaluation of abnormal liver function tests. Postgr Med J. 2003;79:307–12.
10. Huang R, Jia B, Wang G, Wu C. Non-invasive fibrosis markers for chronic hepatitis B in sub-Saharan Africa. Liver Int. 2017;37(11):1738–1738.
11. Vaughan JL, Fourie J, Naidoo S, Subramony N, Wiggill T, Alli N. Prevalence and causes of thrombocytopenia in an academic state-sector laboratory in Soweto, Johannesburg, South Africa. S Afr Med J. 2015;105(3):215–9.
12. Yilmaz Y, Yonal O, Kurt R, Bayrak M, Aktas B, Ozdogan O. Noninvasive assessment of liver fibrosis with the aspartate transaminase to platelet ratio index (APRI): Usefulness in patients with chronic liver disease: APRI in chronic liver disease. Hepat Mon. 2011;11(2):103–6.
13. Lemoine M, Shimakawa Y, Nayagam S, Khalil M, Suso P, Lloyd J, et al. The gamma-glutamyl transpeptidase to platelet ratio (GPR) predicts significant liver fibrosis and cirrhosis in patients with chronic HBV infection in West Africa. Gut. 2016;65(8):1369–76.
14. Tag-Adeen M, Omar MZ, Abd-Elsalam FM, Hasaneen A, Mohamed MA, Elfeky HM, et al. Assessment of liver fibrosis in Egyptian chronic hepatitis B patients. Medicine (Baltimore). 2018;97(6):e9781.
15. Desalegn H, Aberra H, Berhe N, Gundersen SG, Johannessen A. Are non-invasive fibrosis markers

For peer review only

for chronic hepatitis B reliable in sub-Saharan Africa? Liver Int. 2017 37(10):1461–7.

16. Udompap P, Sukonrut K, Suvannarerg V, Pongpaibul A, Charatcharoenwitthaya P. Prospective Comparison of Transient Elastography, Point Shear Wave Elastography, APRI, and FIB-4 for Staging Liver Fibrosis in Chronic Viral Hepatitis. J Viral Hepat. 2019;jvh.13246.
17. Mu R, Chen W, Pan B, Wang L, Hao X, Huang X, et al. First definition of reference intervals of liver function tests in China: a large-population-based multi-center study about healthy adults. PLoS One. 2013;8(9):e72916.
18. Karita E, Ketter N, Price MA, Kayitenkore K, Kaleebu P, Nanvubya A, et al. CLSI-derived hematology and biochemistry reference intervals for healthy adults in eastern and southern Africa. PLoS One. 2009;4(2).
19. Melkie M, Yigeremu M, Nigussie P, Asrat S, Gebreegziabher T, Teka T, et al. Robust reference intervals for liver function test (LFT) analytes in newborns and infants. BMC Res Notes. 2012. 5:493.
20. Rustad P, Felding P, Franzson L, Kairisto V, Lahti A, Mårtensson A, et al. The Nordic Reference Interval Project 2000: recommended reference intervals for 25 common biochemical properties. Scand J Clin Lab Invest. 2004;64(4):271–84.
21. Neuschwander-Tetri BA, Unalp A, Creer MH, Nonalcoholic Steatohepatitis Clinical Research Network. Influence of local reference populations on upper limits of normal for serum alanine aminotransferase levels. Arch Intern Med. 2008;168(6):663–6.
22. Stevens W, Kamali A, Karita E, Anzala O, Sanders EJ, Jaoko W, et al. Baseline Morbidity in 2,990 Adult African Volunteers Recruited to Characterize Laboratory Reference Intervals for Future HIV Vaccine Clinical Trials. PLoS One. 2008;3(4):e2043.
23. Asiki G, Murphy GA V, Baisley K, Nsubuga RN, Karabarinde A, Newton R, et al. Prevalence of dyslipidaemia and associated risk factors in a rural population in South-Western Uganda: a community based survey. PLoS One. 2015;10(5):e0126166.
24. Murphy A, Av G, Rebecca N, Elizabeth H. Sociodemographic distribution of non-communicable disease risk factors in rural Uganda : a cross-sectional study . Int J Epidemiol 2013. 2014;42(6):1740–1753.
25. Stockdale AJ, Phillips RO, Geretti AM, HEPIK Study Group. The gamma-glutamyl transpeptidase to platelet ratio (GPR) shows poor correlation with transient elastography measurements of liver fibrosis in HIV-positive patients with chronic hepatitis B in West Africa. Response to: “The gamma-glutamyl transpeptidase to platelet ratio (GPR) predicts significant liver fibrosis and cirrhosis in patients with chronic HBV infection in West Africa” by Lemoine et al. Gut. 2016;65(5):882–4.

26. Lin Z-H, Xin Y-N, Dong Q-J, Wang Q, Jiang X-J, Zhan S-H, et al. Performance of the aspartate

For peer review only - http://bmjopen.bmj.com/site/about/guidelines.xhtml

aminotransferase-to-platelet ratio index for the staging of hepatitis C-related fibrosis: An updated meta-analysis. Hepatology. 2011;53(3):726–36.

27. Sterling RK, Lissen E, Clumeck N, Sola R, Correa MC, Montaner J, et al. Development of a simple noninvasive index to predict significant fibrosis in patients with HIV/HCV coinfection. Hepatology. 2006;43(6):1317–25.

28. Chen B, Ye B, Zhang J, Ying L, Chen Y. RDW to platelet ratio: a novel noninvasive index for predicting hepatic fibrosis and cirrhosis in chronic hepatitis B. PLoS One. 2013;8(7):e68780.

29. Tag-Adeen M, Omar MZ, Abd-Elsalam FM, Hasaneen A, Mohamed MA, Elfeky HM, et al. Assessment of liver fibrosis in Egyptian chronic hepatitis B patients: A comparative study including 5 noninvasive indexes. Medicine (Baltimore). 2018 97(6):e9781.

30. Adland E, Jesuthasan G, Downs L, Wharton V, Wilde G, McNaughton AL, et al. Hepatitis virus (HCV) diagnosis and access to treatment in a UK cohort. BMC Infect Dis. 2018;18(1):461.

31. Population Attributable Risk (PAR). In: Encyclopedia of Public Health. Springer Netherlands; 2008. p. 1117–8.

32. Yap CY, Choon Aw T. Liver Function Tests (LFTs). Vol. 19, Proceedings of Singapore Healthcare. 2010

33. World health Organisation: Guidelines for the care and treatment of persons diagnosed with chronic hepatitis C virus infection. 2018. http://apps.who.int/iris/bitstream/handle/10665/273174/9789241550345-eng.pdf?ua=1

34. World health Organisation: Guidelines for the prevention, care and treatment of persons with chronic hepatitis B infection. 2015. http://apps.who.int/iris/bitstream/handle/10665/154590/9789241549059_eng.pdf?sequence=1

35. O'Hara GA, McNaughton AL, Maponga T, Jooste P, Ocama P, Chilengi R, et al. Hepatitis B virus infection as a neglected tropical disease. PLoS Negl Trop Dis. 2017;11(10):e0005842.

36. Lemoine M, Shimakawa Y, Njie R, Taal M, Ndow G, Chemin I, et al. Acceptability and feasibility of a screen-and-treat programme for hepatitis B virus infection in The Gambia: the Prevention of Liver Fibrosis and Cancer in Africa (PROLIFICA) study. Lancet Glob Heal. 2016;4(8):e559-67.

37. Hall P, Cash J. What is the real function of the liver "function" tests? Ulster Med J. 2012;81(1):30–6.

38. Opio CK, Seremba E, Ocama P, Lalitha R, Kagimu M, Lee WM. Diagnosis of alcohol misuse and alcoholic liver disease among patients in the medical emergency admission service of a large urban hospital in Sub-Saharan Africa; a cross sectional study. Pan Afr Med J. 2013;15:23.

39. Kabwama SN, Ndyanabangi S, Mutungi G, Wesonga R, Bahendeka SK, Guwatudde D. Alcohol use among adults in Uganda: findings from the countrywide non-communicable diseases risk factor cross-sectional survey. Glob Health Action. 2016;9(1):31302.

For peer review only - http://bmjopen.bmj.com/site/about/guidelines.xhtml

40. Akinyemiju T, Abera S, Ahmed M, Alam N, Alemayohu MA, Allen C, et al. The Burden of Primary Liver Cancer and Underlying Etiologies From 1990 to 2015 at the Global, Regional, and National Level. JAMA Oncol. 2017;3(12):1683.
41. Sterling RK, Chiu S, Snider K, Nixon D. The Prevalence and Risk Factors for Abnormal Liver Enzymes in HIV-Positive Patients without Hepatitis B or C Coinfections. Dig Dis Sci. 2008;53(5):1375–82.
42. Osakunor DNM, Obirikorang C, Fianu V, Asare I, Dakorah M. Hepatic Enzyme Alterations in HIV Patients on Antiretroviral Therapy: A Case-Control Study in a Hospital Setting in Ghana. PLoS One. 2015;10(8):e0134449.
43. Crane M, Iser D, Lewin SR. Human immunodeficiency virus infection and the liver. World J Hepatol. 2012;4(3):91.
44. Mata-Marín JA, Gaytán-Martínez J, Grados-Chavarría BH, Fuentes-Allen JL, Arroyo-Anduiza CI, Alfaro-Mejía A. Correlation between HIV viral load and aminotransferases as liver damage markers in HIV infected naive patients: A concordance cross-sectional study. Virol J. 2009;6:4–7.
45. Orlien SMS, Ismael NY, Ahmed TA, Berhe N, Lauritzen T, Roald B, et al. Unexplained chronic liver disease in Ethiopia: A cross-sectional study. BMC Gastroenterol. 2018 Feb 13;18(1).
46. Kuniholm MH, Lesi OA, Mendy M, Akano AO, Sam O, Hall AJ, et al. Aflatoxin exposure and viral hepatitis in the etiology of liver cirrhosis in the Gambia, West Africa. Environ Health Perspect. 2008;116(11):1553–7.
47. Kang M-S, Nkurunziza P, Muwanika R, Qian G, Tang L, Song X, et al. Longitudinal evaluation of aflatoxin exposure in two cohorts in south-western Uganda. Food Addit Contam Part A. 2015;32(8):1322–30.
48. Liu Y, Wu F. Global burden of aflatoxin-induced hepatocellular carcinoma: a risk assessment. Environ Health Perspect. 2010;118(6):818–24.
49. Almobarak AO, Barakat S, Khalifa MH, Elhoweris MH, Elhassan TM, Ahmed MH. Non alcoholic fatty liver disease (NAFLD) in a Sudanese population: What is the prevalence and risk factors? Arab J Gastroenterol. 2014;15(1):12–5.
50. Dyson JK, Anstee QM, McPherson S. Non-alcoholic fatty liver disease: a practical approach to diagnosis and staging. Frontline Gastroenterol. 2014 5(3):211–8.
51. Onyekwere CA, Ogbera AO, Balogun BO. Non-alcoholic fatty liver disease and the metabolic syndrome in an urban hospital serving an African community. Ann Hepatol.;10(2):119–24.
52. Argo CK, Caldwell SH. Epidemiology and Natural History of Non-Alcoholic Steatohepatitis. Clin Liver Dis. 2009;13(4):511–31.
53. Williams CD, Stengel J, Asike MI, Torres DM, Shaw J, Contreras M, et al. Prevalence of

For peer review only

Nonalcoholic Fatty Liver Disease and Nonalcoholic Steatohepatitis Among a Largely Middle-Aged Population Utilizing Ultrasound and Liver Biopsy: A Prospective Study. Gastroenterology. 2011;140(1):124–31.

FIGURE LEGENDS

Fig 1: Liver function tests and hepatic fibrosis scores among adults in the Uganda General Population Cohort. Distribution of (A) ALT, (B) AST and (C) GGT. Dashed vertical lines indicate upper limit of normal (ULN) based on American reference range, ARR (blue) and local reference range, LRR (red), as shown in Suppl Table 2 (5). Note no LRR for GGT. (D) Proportion of the population with an elevated GPR score, and among those with elevated GPR the proportion with a defined risk factor for fibrosis. (E) Proportion of the population with an elevated AST/ALT ratio, and among those with an elevated ratio the proportion with a self-reported history of alcohol intake.

Fig 2: Forrest plots to show odds ratio (OR) for host risk factors and elevated LFTs or fibrosis scores in the Uganda General Population Cohort. Data are presented for the final multivariate model for ALT, AST, APRI, GPR, and AST/ALT, showing variables that were independently associated with the outcome (statistically significant at the $P<0.05$ level after adjusting for other variables).

For peer review only

SUPPLEMENTARY DATA

All supporting data are accessible on-line at FigShare: O'Hara, Geraldine; Mokaya, Jolynne; Hau, Jeffrey; Downs, Louise; Karabarinde, Alex; Asiki, Gershim; et al. (2019): Liver function tests and fibrosis scores in a rural population in Africa. figshare. Dataset. https://doi.org/10.6084/m9.figshare.8292194

Metadata table: raw data for 8145 adults in the Uganda General Population cohort (available as .xls and .csv files)

STROBE statement: checklist of items that should be included in reports of observational studies (pdf file)

Supporting data file (pdf file) contains the following tables and figures:

Suppl Table 1: Origin, reference ranges and clinical significance of liver function tests (LFTs)

Suppl Table 2: Scores to estimate liver fibrosis, calculated from liver function tests

Suppl Table 3: Description of characteristics of study participants with liver function test (LFT) results from the Ugandan General Population Cohort (N=8,099)

Suppl Table 4: Median and inter-quartile range for each liver function test, with the population divided by risk factors

Suppl Fig 1: Distribution of liver function tests in Uganda General Population Cohort. Dashed vertical lines indicate upper limit of normal (ULN) based on American reference range, ARR (orange line is the ULN for female; blue line is the ULN for males) and local reference range, LRR (black), as shown in Suppl Table 1. Note no LRR for GGT. ULN for bilirubin using ARR is the same for both male and female, indicated by red dashed line. Data are shown for study participants aged ≥16 years, apart from ALP which is shown for participants aged ≥20 to exclude teenagers who may have elevated ALP as a normal physiological consequence of bone growth.

Suppl Fig 2: Odds ratio for deranged ALT, AST, APRI, GPR and AST/ALT among participants grouped by sex and age

For peer review only

Suppl Fig 3: Proportion of Uganda General Population cohort reporting alcohol consumption among individuals with and without AST/ALT ratio >2

Suppl Fig 4: Proportion of Uganda General Population Cohort with elevated GGT, according to AST/ALT ratio.

Suppl Fig 5: Proportion of Uganda General Population Cohort with blood borne virus (BBV) infection, according to GPR score.

For peer review only

TABLES

Table 1: Study participants from the Uganda General Population Cohort with abnormal LFT results and fibrosis scores based on upper limit of normal according to American reference range (ARR) and local reference ranges (LRR).

Enzyme Type	*Total* n / N (%)	*Male* n / N (%)	*Female* n / N (%)	*p value*[1]
***ALT*[2]**				
Abnormal ARR*	573 / 8,099 (7.1)	162 / 3,542 (4.6)	411 / 4,557 (9.0)	<0.001
Abnormal LRR**	209 / 8,099 (2.6)	87 / 3,542 (2.5)	122 / 4,557 (2.7)	0.53
***AST*[2]**				
Abnormal ARR*	1,011 / 8,099 (12.5)	434 / 3,542 (12.3)	577 / 4,557 (12.7)	0.58
Abnormal LRR**	241 / 8,099 (3.0)	123 / 3,542 (3.5)	118 / 4,557 (2.6)	0.02
***GGT*[2,3]**				
Abnormal ARR*	889 / 8,099 (11.0)	362 / 3,542 (10.2)	527 / 4,557 (11.6)	0.06
***BR*[2]**				
Abnormal ARR*	1,051 / 8,099 (13.0)	635 / 3,542 (18.0)	416 / 4,557 (9.1)	<0.001
Abnormal LRR**	497 / 8,099 (6.1)	214 / 3,542 (6.0)	283 / 4,557 (6.2)	0.75
***ALP*[2,4]**				
Abnormal ARR*	1,161 / 5,616 (20.7)	315 / 2,273 (13.9)	846 / 3,343 (25.3)	<0.001
Abnormal LRR**	139 / 5,616 (2.5)	60 / 2,273 (2.6)	79 / 2,273 (2.4)	0.513
***FIB-4*[2]**				
Abnormal***	99 / 1,877 (5.3)	54 / 824 (6.6)	45 / 1,053 (4.3)	0.03
***APRI*[2,5]**				
Abnormal ARR*,***	145 / 1,877 (7.7)	95 / 824 (11.5)	50 / 1,053 (4.8)	<0.001
Abnormal LRR*, ***	60 / 1,877 (3.2)	42 / 824 (5.1)	18 / 1,053 (1.7)	<0.001
***GPR*[2]**				
Abnormal***	441 / 1,877 (23.5)	185 / 824 (22.5)	256 / 1,053 (24.3)	0.35
***AST/ALT*[2]**				
Abnormal***	882 / 8,099 (10.9)	420 / 3,542 (11.9)	462 / 4,557 (10.1)	0.01
***S-Index*[2]**				
Abnormal***	73 / 1,877 (3.9)	50 / 824 (6.1)	23 / 1,053 (2.2)	<0.001

[1] p-value calculated to determine whether significant difference between males and females in each category using chi-square test. [2] ALT - Alanine Transminase, AST - Aspartate Transminase, GGT - Gamma-glutamyl transpeptidase, ALP - Alkaline Phosphatase, BR - Total Bilirubin, FIB-4 - fibrosis 4, APRI - AST to Platelet Ratio Index, GPR - GGT to platelet ratio, AST/ALT ratio - Aspartate/ Alanine ratio. [3] LRR for GGT not defined. [4] Individuals under the age of 19 were excluded.[5] APRI score calculated using ULN of AST using both the ARR and LRR.
* Abnormal LFTs, according to ARR, are defined as test results outside of the following ranges: ALT (Male: 10 – 55 U/L, Female: 7 – 30 U/L), AST (Male: 10 – 40 U/L, Female: 9 – 32 U/L), GGT (Male: 8 – 61 U/L, Female: 5 – 36 U/L), BR (0 – 17 mmol/L), ALP (Male: 45 – 115 U/L, Female: 30 – 100 U/L). ** Abnormal LFTs, according to LRR, are defined as test results outside of the following ranges: ALT (8 – 61 U/L), AST (14 – 60 U/L), BR (2.9 – 37 mmol/L), ALP (48 – 164 U/L). *** Threshold used to predict liver fibrosis: APRI > 0.7; FIB-4 >3.25; GPR >0.32; RPR >0.825; S-Index >0.3.

For peer review only - http://bmjopen.bmj.com/site/about/guidelines.xhtml

Table 2: Univariate and multivariate analysis for factors associated with abnormal liver function tests according to American reference ranges (ARR) for ALT, AST, ALP, GGT, and TB, and laboratory markers of fibrosis in adults in the Uganda General Population Cohort.

	ALT [1,6] *OR (95% CI)*	*AST* [1,6] *OR (95% CI)*	*ALP* [1,4,6,] *OR (95% CI)*	*GGT* [1,6] *OR (95% CI)*	*TB* [1,6] *OR (95% CI)*	*FIB-4* [1,7] *OR (95% CI)*	*APRI* [1,7,#] *OR (95% CI)*	*GPR* [1,7] *OR (95% CI)*	*AST/ALT* [1,7] *OR (95% CI)*	*S-Index* [3,7] *OR (95% CI)*
UNIVARIATE ANALYSIS										
Sex										
Male	*Ref*	*Ref*	*Ref*	*Ref*	*Ref*	*Ref*	*Ref*	*Ref*	*Ref*	*Ref*
Female	2.06 (1.71,2.49)***	1.04 (0.91,1.18)^{ns}	0.93 (0.84,1.01)^{ns}	1.15 (1.00,1.32)*	0.46 (0.20,0.24)***	0.64 (0.42,0.96)*	0.38 (0.27,0.55)***	1.10 (0.89,1.38)^{ns}	0.84 (0.73,0.96)*	0.35 (0.21,0.57)***
Age										
<19	*Ref*	*Ref*	-	*Ref*	*Ref*	*Ref* [5]	*Ref*	*Ref*	*Ref*	*Ref* [5]
20-29	1.33 (1.03,1.73)*	0.9 (0.73,1.11)^{ns}	*Ref* [4]	2.61 (1.92,3.56)***	1.46 (1.22,1.75)***		2.57 (1.41,4.71)**	2.63 (1.72,4.03)***	0.55 (0.43,0.70)***	
30-39	1.58 (1.22,2.04)***	1.17 (0.95,1.43)^{ns}	0.72 (0.60,087)***	6.59 (5.00,8.72)***	1.15 (0.94,1.39)^{ns}		3.15 (1.76,5.68)***	6.22 (4.21,9.18)***	0.67 (0.53,0.85)**	
40-49	1.41 (1.04,1.87)*	1.47 (1.12,1.80)***	0.48 (0.38,0.59)***	8.34 (6.29,11.07)***	1.02 (0.83,1.27)^{ns}	8.48 (3.95,18.18)***	4.00 (2.22,7.18)***	7.63 (5.12,11.36)***	0.83 (0.65,1.05)^{ns}	5.02 (2.79,9.68)***
50-59	1.38 (1.00,1.90)*	1.57 (1.25,2.00)***	0.82 (0.66,1.02)^{ns}	8.03 (5.93,10.86)***	0.92 (0.71,1.18)^{ns}	14.60 (9.86,31.03)***	3.50 (1.80,6.73)***	9.10 (5.91,14.0)***	1.11 (0.86,1.43)^{ns}	4.71 (2.31,9.59)***
>60	1.39 (1.03,1.88)*	1.24 (0.98,1.55) ^{ns}	1.28 (1.06,1.54)**	6.84 (5.09,9.20)***	0.56 (0.42,0.74)***	34.88 (17.80,68.39)***	3.68 (2.00,7.00)***	8.20 (5.42,12.41)***	2.23 (1.82,2.72)***	5.43 (2.84,10.39)***
Alcohol										
No	*Ref*	*Ref*	*Ref*	*Ref*	*Ref*	*Ref*	*Ref*	*Ref*	*Ref*	*Ref*
Yes	1.41 (1.16,1.70)***	1.57 (1.35,1.83)***	1.0 (0.86,1.13)***	2.14 (1.83,2.51)***	0.99 (0.85,1.15)^{ns}	2.02 (1.22,3.32)**	1.60 (1.04,2.31)*	2.10 (1.61,2.66)***	1.28 (1.08,1.50)**	6.09 (3.16,11.72)***
BMI [2]										
Normal	*Ref*	*Ref*	*Ref*	*Ref*	*Ref*	*Ref*	*Ref*	*Ref*	*Ref*	*Ref*
Under-weight	1.41 (1.12,1.77)**	1.45 (1.23,1.71)***	1.17 (0.96,1.44)^{ns}	1.42 (1.16,1.73)**	0.69 (0.57,0.83)***	1.78 (1.06,3.00)^{ns}	1.78 (1.10,2.60)*	1.07 (0.78,1.50)^{ns}	1.62 (1.37,1.92)***	1.87 (1.04,3.33)*
Over-weight	1.10 (0.85,1.41)^{ns}	0.73 (0.58,0.92)**	0.93 (0.77,1.13)^{ns}	1.36 (1.11,1.66)**	0.75 (0.59,0.95)*	0.74 (0.35,1.56)^{ns}	0.91(0.50,1.65)^{ns}	1.15 (0.82,1.60)^{ns}	0.57 (0.42,0.76)***	0.87 (0.38,2.03)^{ns}
HIV status										
Negative	*Ref*	*Ref*	*Ref*	*Ref*	*Ref*	*Ref*	*Ref*	*Ref*	*Ref*	*Ref*
Positive	1.63 (1.24,2.15)***	2.30 (1.87,2.83)***	1.47 (1.19,1.81)***	4.83 (3.98,5.85)***	0.21 (0.14,0.33)***	0.28 (0.07,1.20)^{ns}	1.30 (0.68,2.30)^{ns}	3.88 (2.62,5.73)***	1.06 (0.80,1.42)^{ns}	4.00 (2.08,7.69)***
HBV status										
Negative	*Ref*	*Ref*	*Ref*	*Ref*	*Ref*	*Ref*	*Ref*	*Ref*	*Ref*	*Ref*
Positive	2.61 (1.77,3.84)***	2.52 (1.84,3.44)***	1.07 (0.72,1.60)^{ns}	1.80 (1.24,2.60)***	1.10 (0.76,1.60)^{ns}	2.01 (0.62,6.50)^{ns}	3.56 (1.80,7.10)***	4.24 (2.27,7.93)***	0.98 (0.63,0.15)^{ns}	4.92 (2.07,11.69)***
MULTIVARIATE ANALYSIS										
Sex										
Male	*Ref*	*Ref*	*Ref*	*Ref*	*Ref*	*Ref*	*Ref*	*Ref*	*Ref*	*Ref*
Female	2.30 (1.89,2.81)***	1.20 (1.04,1.38)*	2.11 (1.83,2.44)***	1.01 (0.86,1.19)^{ns}	0.46 (0.40,0.53)***	0.62 (0.40,0.97)*	0.42 (0.30,0.62)***	1.11 (0.87,1.41)^{ns}	0.90 (0.78,1.06)^{ns}	0.37 (0.22,0.63)***
Age										
<19	*Ref*	*Ref*	-	*Ref*	*Ref*		*Ref*	*Ref*	*Ref*	
20-29	1.26 (0.95,1.68)^{ns}	0.89 (0.70,1.12)^{ns}	*Ref* [4]	1.69 (1.19,2.41)**	1.52 (1.25,1.84)***	*Ref* [5]	3.22 (1.66,6.22)**	1.86 (1.19,2.92)**	0.57 (0.44,0.75)***	*Ref* [5]
30-39	1.35 (1.00,1.80)*	1.00 (0.79,1.27)^{ns}	0.68 (0.56,0.82)***	3.96 (2.87,5.46)***	1.29 (1.02,1.59)*		3.55 (1.81,7.00)***	3.70 (2.43,5.66)***	0.72 (0.55,0.95)*	

40-49	1.13 (0.83,1.56)^{ns}	1.20 (0.95,1.52)^{ns}	0.46 (0.37,0.57)***	4.87 (3.54,6.70)***	1.17 (0.94,1.47)^{ns}	7.04 (3.19,15.52)***	4.00 (2.04,7.82)***	4.45 (2.88,6.87)***	0.93 (0.71,1.21)^{ns}	2.68 (1.37,5.26)**
50-59	1.09 (0.77,1.55)^{ns}	1.29 (0.99,1.67)^{ns}	0.82 (0.66,1.02)^{ns}	5.02 (3.58,7.02)***	1.01 (0.78,1.32)^{ns}	11.29 (5.13,24.80)***	3.45 (1.65,7.22)**	5.75 (3.61,9.15)***	1.22 (0.92,1.61)^{ns}	2.76 (1.29,5.90)**
>60	1.13 (0.81,1.57)^{ns}	1.00 (0.78,1.30)^{ns}	1.32 (1.09,1.59)**	4.98 (3.59,6.90)***	0.60 (0.45,0.80)***	25.15 (12.32,51.35)***	3.50 (1.73,7.11)**	5.39 (3.42,8.47)***	2.20 (1.74,2.77)***	3.34 (1.63,6.84)**
Alcohol										
No	*Ref*	*Ref*	-	*Ref*	-	*Ref*	*Ref*	*Ref*	*Ref*	*Ref*
Yes	1.33 (1.09,1.63)**	1.53 (1.30,1.78)***	-	2.00 (1.69,2.36)***	-	2.05 (1.24,3.40)**	1.51 (1.00,2.27)*	1.96 (1.52,2.54)***	1.26 (1.06,1.50)**	5.23 (2.72,10.04)***
BMI [2]										
Normal	*Ref*	*Ref*	-	*Ref*	*Ref*	-	*Ref*	-	*Ref*	-
Underweight	1.40 (1.11,1.75)**	1.44 (1.21,1.70)***	-	1.37 (1.11,1.68)**	0.70 (0.58,0.83)***	-	1.72 (1.11,2.65)*	-	1.61 (1.36,1.91)***	-
Overweight	1.12 (0.87,1.44)^{ns}	0.75 (0.60,0.95)*	-	1.47 (1.19,1.82)***	0.72 (0.57,0.92)**	-	0.95 (0.52,1.73)^{ns}	-	0.56 (0.42,0.76)***	-
HIV status										
Negative	*Ref*	*Ref*	*Ref*	*Ref*	*Ref*	-	-	*Ref*	-	*Ref*
Positive	1.59 (1.20,2.10)***	2.13 (1.72,2.63)***	1.47 (1.19,1.81)***	4.76 (3.89,5.82)***	0.22 (0.14,0.34)***	-	-	3.84 (2.58,5.70)***	-	3.58 (1.84,6.94)***
HBV status										
Negative	*Ref*	*Ref*	-	*Ref*	-	-	*Ref*	*Ref*	-	*Ref*
Positive	2.61 (1.76,3.86)***	2.40 (1.74,3.31)***	-	1.65 (1.11,2.45)*	-	-	3.60 (1.79,7.27)***	4.26 (2.23,8.12)***	-	4.37 (1.80,10.58)***

[1] ALT - Alanine Transminase, AST - Aspartate Transminase, GGT - Gamma-glutamyl transpeptidase, ALP - Alkaline Phosphatase, BR -Total Bilirubin, FIB-4 - fibrosis 4, APRI - AST to Platelet Ratio Index, GPR - GGT to plate Alanine ratio. OR - odds ratio.
[2] Body Mass Index (BMI) Classification according to WHO (weight/height^2: kg/m^2): Underweight (<18.5 kg/m^2), Normal weight (18.5 – 24.99 kg/m^2), Overweight (25.0 – 29.99 kg/m^2), Obese (>30.0 kg/m^2)
[3] An S-index score of >0.3 is suggestive of liver fibrosis
[4] Individuals under the age of 19 were excluded. Reference age group is 20 – 29
[5] Reference age group consists of all individuals under the age of 39
[6] Abnormal LFTs, according to ARR, are defined as test results outside of the following ranges: ALT (Male: 10 – 55 U/L, Female: 7 – 30 U/L), AST (Male: 10 – 40 U/L, Female: 9 – 32 U/L), GGT (Male: 8 – 61 U/L, Female: 5 – 36 U/L), BR (0 – 17 mmol/L), ALP (Male: 45 – 115 U/L, Female: 30 – 100 U/L)
[7] Threshold used to predict liver fibrosis: APRI > 0.7. FIB-4 >3.25. GPR >0.32. RPR >0.825. S-Index >0.3
[#] APRI score calculated using ULN of AST using African reference range
Significance level: * = (p<0.05), ** = (p<0.01), *** = (p<0.001), *ns* = (p>0.05)

For peer review only

For peer review only - http://bmjopen.bmj.com/site/about/guidelines.xhtml

Table 3: Relative risk, population attributable risk (PAR) percent, and the number of individuals with abnormal liver function tests in the Uganda General Population Cohort. Analysis according to American reference ranges (ARR for ALT, AST, ALP, GGT, and TB)

Variable	ALT [1,3]	AST [1,3]	ALP [1,3]	GGT [1,3]	TB [1,3]	FIB-4 [1,4]	APRI [1,4,#]	GPR [1,4]	AST/ALT [1,4]	S-Index [2,4]
**Alcohol+**										
Abnormal Result n (%)	248 (8.5)	467 (16.0)	533 (19.6)	555 (19)	381 (13.1)	72 (11.0)	80 (12.25)	260 (39.8)	379 (13.0)	60 (9.2)
RR (95% CI)[1]	1.4 (1.2 – 1.6)	1.5 (1.4 – 1.7)	1.2 (0.9 – 1.7)	2.9 (2.6 – 3.4)	1.0 (0.9 – 1.1)	5.0 (3.2 – 7.7)	2.3 (1.7 – 3.2)	2.7 (2.3 – 3.2)	1.3 (1.2 – 1.5)	8.7 (4.8 – 15.6)
PAR (%)[1,6]	11.3%	15.9%	0.6%	41.3%	0.3%	58.2%	31.3%	37.1%	10.8%	72.7%
Adj. PAR (%)[5,6]	10.0%	13.9%	-2.6%	26.7%	1.0%	32.4%	16.2%	19.4%	8.0%	64.0%
**HIV+**										
Abnormal Result n (%)	71 (11.7)	144 (23.7)	142 (24.8)	227 (37.3)	21 (3.5)	2 (1.6)	14 (11.0)	73 (57.5)	59 (9.7)	15 (11.8)
RR (95% CI)[1]	1.7 (1.4 – 2.2)	2.0 (1.8 – 2.4)	1.2 (1.1 – 1.4)	4.2 (3.7 – 4.8)	0.3 (0.2 – 0.4)	0.3 (0.1– 1.1)	1.5 (0.9 – 2.5)	2.7 (2.3 – 3.3)	0.9 (0.7 – 1.1)	3.6 (2.1 – 6.1)
PAR (%)[1,6]	5.3%	7.3%	2.2%	19.5%	-6.0%	-5.09%	3.1%	10.5%	-0.9%	14.7%
Adj. PAR (%)[5,6]	4.3%	6.5%	1.1%	17.6%	-6.0%	-4.6%	1.4%	8.3%	-0.1%	13.6%
**HBV+**										
Abnormal Result n (%)	33 (15.0)	56 (25.4)	32 (19.5)	39 (17.7)	35 (16)	4 (8.2)	13 (26.53)	25 (51.0)	22 (10.0)	8 (16.3)
RR (95% CI)[1]	2.2 (1.6 – 3.0)	2.1 (1.7 – 2.7)	0.9 (0.7 – 1.3)	1.6 (1.2 – 2.2)	1.2 (0.9 – 1.7)	1.6 (0.6 – 4.1)	1.5 (0.9 – 2.5)	2.2 (1.7 – 3.0)	0.9 (0.6 – 1.4)	4.6 (2.3 – 9.0)
PAR (%)[1,6]	3.1%	2.9%	-0.2%	1.7%	0.6%	1.5%	3.1%	3.1%	-0.2%	8.6%
Adj. PAR (%)[5,6]	3.3%	2.8%	0.02%	1.4%	0.2%	1.4%	5.7%	2.9%	-0.3%	7.6%

[1] ALT - Alanine Transminase, AST - Aspartate Transminase, GGT - Gamma-glutamyl transpeptidase, ALP - Alkaline Phosphatase, BR - Total Bilirubin, FIB-4 - fibrosis 4, APRI - AST to Platelet Ratio Index, GPR - GGT to platelet ratio, AST/ALT ratio - Aspartate/ Alanine ratio, RR - relative risk, PAR (%) - population attributable risk percent, 95% CI denotes 95% confidence interval
[2] An S-index score of >0.3 is suggestive of liver fibrosis
[3] Abnormal LFTs, according to ARR, are defined as test results outside of the following ranges: ALT (Male: 10 – 55 U/L, Female: 7 – 30 U/L), AST (Male: 10 – 40 U/L, Female: 9 – 32 U/L), GGT (Male: 8 – 61 U/L, Female: 5 – 36 U/L), BR (0 – 17 mmol/L), ALP (Male: 45 – 115 U/L, Female: 30 – 100 U/L)
[4] Threshold used to predict liver fibrosis: APRI > 0.7. FIB-4 >3.25. GPR >0.32. RPR >0.825. S-Index >0.3
[5] Adjusted for age, sex, alcohol consumption, HBV diagnosis, HIV status, and Body Mass Index.
[6] A measure of zero indicates of no association between the risk factor and abnormal liver function tests. A positive value indicates that the exposure to the risk factor is a risk factor, while a negative value indicates that it is a protective factor.
[#] APRI score calculated using ULN of AST using African reference range
+ number of abnormal result, RR and PAR (%) are based on individuals who were classified as positives within each variable (ie. Alcohol drinkers, HIV positive, HBV positive)

review only

For peer review only - http://bmjopen.bmj.com/site/about/guidelines.xhtml

For peer review only

For peer review only - http://bmjopen.bmj.com/site/about/guidelines.xhtml

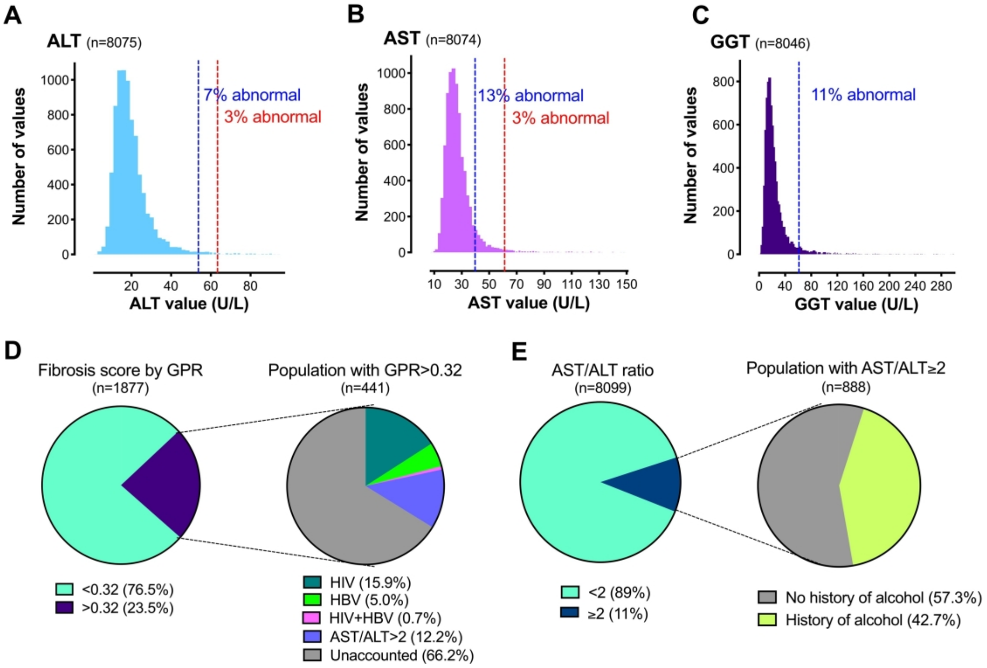

291x198mm (300 x 300 DPI)

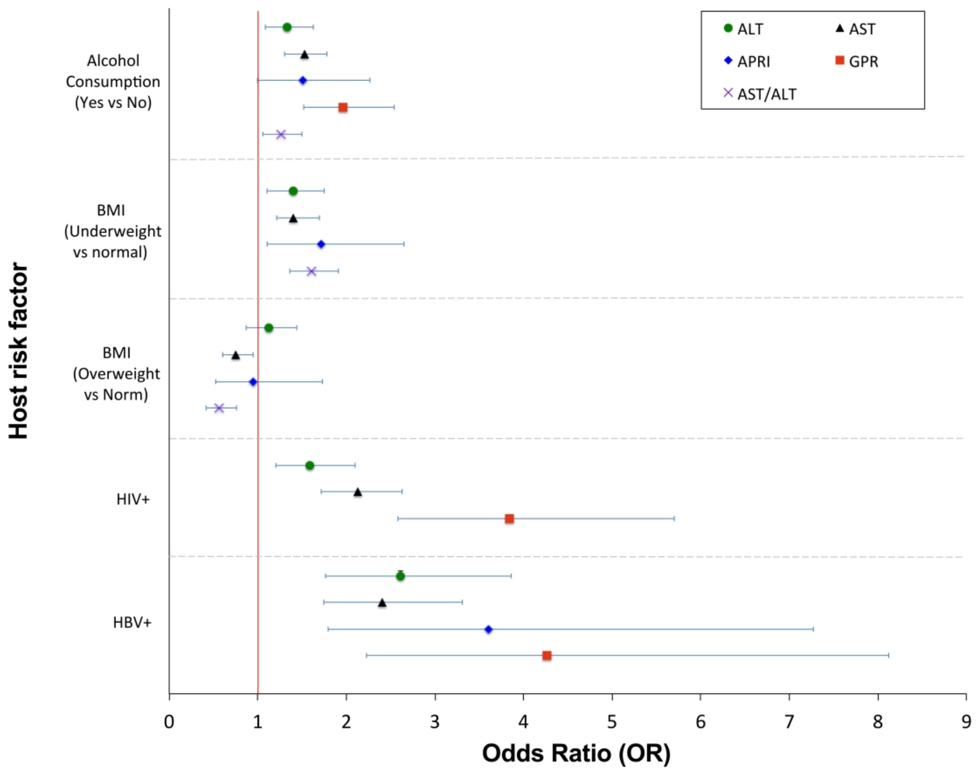

226x180mm (600 x 600 DPI)

STROBE Statement
checklist of items that should be included in reports of observational studies

Liver function tests and fibrosis scores in a rural population in Africa: a cross-sectional study to estimate the burden of disease and associated risk factors

	Item No	Recommendation	Location in manuscript
**Title and abstract**	1	(*a*) Indicate the study's design with a commonly used term in the title or the abstract	Title specifies a cross-sectional study, page 1
		(*b*) Provide in the abstract an informative and balanced summary of what was done and what was found	Provided in abstract, page 3
**Introduction**			
Background/rationale	2	Explain the scientific background and rationale for the investigation being reported	Included in introduction, page 5-6
Objectives	3	State specific objectives, including any prespecified hypotheses	Final paragraph of introduction, page 6
**Methods**			
Study design	4	Present key elements of study design early in the paper	First paragraph of methods, page 7
Setting	5	Describe the setting, locations, and relevant dates, including periods of recruitment, exposure, follow-up, and data collection	First two paragraphs of methods, page 7
Participants	6	(*a*) *Cohort study*—Give the eligibility criteria, and the sources and methods of selection of participants. Describe methods of follow-up<br>*Case-control study*—Give the eligibility criteria, and the sources and methods of case ascertainment and control selection. Give the rationale for the choice of cases and controls<br>*Cross-sectional study*—Give the eligibility criteria, and the sources and methods of selection of participants	Included in methods section, page 7
		(*b*) *Cohort study*—For matched studies, give matching criteria and number of exposed and unexposed<br>*Case-control study*—For matched studies, give matching criteria and the number of controls per case	Not applicable
Variables	7	Clearly define all outcomes, exposures, predictors, potential confounders, and effect modifiers. Give diagnostic criteria, if applicable	Included in methods page 7; further details of blood parameters provided in suppl data tables
Data sources/ measurement	8*	For each variable of interest, give sources of data and details of methods of assessment (measurement). Describe comparability of assessment methods if there is more than one	Included in methods, page 7 and suppl data table 1

For peer review only - http://bmjopen.bmj.com/site/about/guidelines.xhtml

		group	
Bias	9	Describe any efforts to address potential sources of bias	Included in 1st paragraph of discussion, page 12
Study size	10	Explain how the study size was arrived at	Pragmatic approach; data sources described in 1st paragraph of methods
Quantitative variables	11	Explain how quantitative variables were handled in the analyses. If applicable, describe which groupings were chosen and why	Described in methods page 8 and suppl table 2
Statistical methods	12	(*a*) Describe all statistical methods, including those used to control for confounding	Described in methods page 8
		(*b*) Describe any methods used to examine subgroups and interactions	Described in methods page 8
		(*c*) Explain how missing data were addressed	Described in methods
		(*d*) *Cohort study*—If applicable, explain how loss to follow-up was addressed<br>*Case-control study*—If applicable, explain how matching of cases and controls was addressed<br>*Cross-sectional study*—If applicable, describe analytical methods taking account of sampling strategy	Not applicable
		(*e*) Describe any sensitivity analyses	Not applicable

Results

Participants	13*	(a) Report numbers of individuals at each stage of study—eg numbers potentially eligible, examined for eligibility, confirmed eligible, included in the study, completing follow-up, and analysed	Provided in methods and denominators specified in tables
		(b) Give reasons for non-participation at each stage	Not applicable
		(c) Consider use of a flow diagram	Not applicable
Descriptive data	14*	(a) Give characteristics of study participants (eg demographic, clinical, social) and information on exposures and potential confounders	Suppl table 3
		(b) Indicate number of participants with missing data for each variable of interest	All denominators presented in tables
		(c) *Cohort study*—Summarise follow-up time (eg, average and total amount)	Not applicable
Outcome data	15*	*Cohort study*—Report numbers of outcome events or summary measures over time	Not applicable
		Case-control study—Report numbers in each exposure category, or summary measures of exposure	Not applicable
		Cross-sectional study—Report numbers of outcome events or summary measures	All denominators presented in tables
Main results	16	(*a*) Give unadjusted estimates and, if applicable, confounder-adjusted estimates and their precision (eg, 95% confidence interval). Make clear which confounders were adjusted for and why they were included	Tables
		(*b*) Report category boundaries when continuous variables were categorized	Boundaries listed in suppl table 1

For peer review only

		(*c*) If relevant, consider translating estimates of relative risk into absolute risk for a meaningful time period	Not applicable
Other analyses	17	Report other analyses done—eg analyses of subgroups and interactions, and sensitivity analyses	Not applicable
**Discussion**			
Key results	18	Summarise key results with reference to study objectives	First paragraph of discussion
Limitations	19	Discuss limitations of the study, taking into account sources of potential bias or imprecision. Discuss both direction and magnitude of any potential bias	Included in discussion page 12-13
Interpretation	20	Give a cautious overall interpretation of results considering objectives, limitations, multiplicity of analyses, results from similar studies, and other relevant evidence	Included in discussion
Generalisability	21	Discuss the generalisability (external validity) of the study results	Included in discussion
**Other information**			
Funding	22	Give the source of funding and the role of the funders for the present study and, if applicable, for the original study on which the present article is based	Financial support statement is included

*Give information separately for cases and controls in case-control studies and, if applicable, for exposed and unexposed groups in cohort and cross-sectional studies.

Note: An Explanation and Elaboration article discusses each checklist item and gives methodological background and published examples of transparent reporting. The STROBE checklist is best used in conjunction with this article (freely available on the Web sites of PLoS Medicine at http://www.plosmedicine.org/, Annals of Internal Medicine at http://www.annals.org/, and Epidemiology at http://www.epidem.com/). Information on the STROBE Initiative is available at www.strobe-statement.org.

For peer review only
